# Thermally driven CO_2_ adsorption/desorption and its effect on soil respiration

**DOI:** 10.1126/sciadv.aed9979

**Published:** 2026-09-23

**Authors:** Anna Abramova, Jennifer Mills, Gregory E. Maurer, Benjamin Gilbert, Melese Getenet, Laura Lammers, Shauna Zahabi, Maedeh Chitsaz, Marco Pfeiffer, Ronald Amundson

**Affiliations:** ^1^ Department of Environmental Science, Policy, and Management, University of California, Berkeley, Berkeley, CA 94720, USA.; ^2^ Jornada Basin LTER Program, New Mexico State University, Las Cruces, NM 88003, USA.; ^3^ Earth and Environmental Sciences, Energy Geosciences, Lawrence Berkeley National Laboratory, Berkeley, CA 94720 USA.

## Abstract

Soil respiration counterbalances global photosynthesis, yet several aspects of this process remain unexplained despite decades of research: (i) nighttime negative soil carbon flux in drylands and (ii) diurnal temperature-dependent hysteresis patterns. We propose that abiotic CO_2_ adsorption-desorption in the soil is the cause or contributor to these phenomena. Laboratory isotherms were conducted on desert soil at various diurnal temperatures and CO_2_ partial pressure levels. These results, combined with field data from the Mojave Desert, demonstrate that adsorption causes negative nocturnal CO_2_ fluxes, while desorption during the day leads to positive fluxes. In addition, by accounting for thermally driven physical CO_2_ exchange, the apparent temperature hysteresis commonly observed is reversed. The annual CO_2_ adsorption in the thermally active soil layer ranges from 16.7 to 19.3 grams of CO_2_-C per square meter per year, indicating a possible abiotic exchange of up to 2.2 to 2.5 billion tonnes of CO_2_-C with the atmosphere globally. Our findings suggest that gaseous adsorption should be included in the global carbon cycle to more accurately represent land-atmosphere CO_2_ exchange.

## INTRODUCTION

Soil respiration—the net flux (release) of CO_2_ from soils to the atmosphere (*1*) (fig. S1)—is the annual transfer of between 68 and 101 billion tonnes (Gt) CO_2_-C year^−1^ to the atmosphere from the soil environment (*2*). This large flux consists of the combined contributions of plant root respiration and the respiration of heterotrophic microbes that oxidize soil organic carbon (C). Soil respiration counterbalances net photosynthetic inputs to global soils. Advances in the past two decades that allow near-continuous measurements of soil respiration rates over daily to annual cycles have revealed two puzzling phenomena: (i) a diurnal, and seasonal, hysteresis of respiration rate versus soil temperature (*3*–*5*) and (ii) nighttime negative soil flux rates in arid to semiarid regions, especially during dry periods (*6*–*8*). Here, we propose that these two seemingly different phenomena are linked and driven by the same process: temperature-dependent physical adsorption of CO_2_ onto soil minerals and organic matter.

Physical scientists demonstrated more than a century ago that common soil minerals can adsorb substantial quantities of CO_2_ and that this is strongly temperature dependent (*9*). Langmuir (*9*) in 1918 found that muscovite, a nonexpanding phyllosilicate mineral, adsorbed 0.2 μmol of CO_2_ per gram of mineral under ambient conditions and that the quantity adsorbed varied positively with CO_2_ pressure and inversely with temperature. More recently, mineralogists have used CO_2_ adsorption isotherms, along with the more standard gas N_2_, to probe the nature of mineral surfaces and their molecular characteristics (*10*). Today, considerable effort is devoted to understanding CO_2_ adsorption behavior on various minerals at the highly elevated pressures associated with subsurface injection of CO_2_ into underground reservoirs (*11*). Yet, a linkage between these fields of study and the soil C flux community appears largely absent. Only one paper has attempted a back-of-the-envelope estimate of the possible amount of CO_2_ adsorbed on global soils (*12*).

One of the important gaps in CO_2_ adsorption studies has been (with some exceptions) experiments on either natural soil or on common soil mineral types, at ambient conditions, and across temperature ranges observed in field conditions, where negative CO_2_ fluxes have been observed. Here, we conducted CO_2_ adsorption isotherm experiments for natural Mojave Desert soil and combined these with observations of nocturnal negative CO_2_ fluxes at a low-elevation Mojave Desert site using continuous in situ soil monitoring data collected over a 2-year period. Using measurements of temperature (*T*), CO_2_ concentrations, and soil water content (θ) from four depths, we (i) calculated surface CO_2_ fluxes, (ii) used laboratory isotherm experiments and modeling, to demonstrate that thermally driven adsorption of CO_2_ is a more likely driver of observed negative fluxes than aqueous physiochemical or biotic processes, and (iii) examined whether thermally driven physical adsorption, when combined with soil respiration measurements, can help explain the apparent temperature hysteresis of soil respiration rates. We conclude that the thermal adsorption/desorption of CO_2_ onto mineral surfaces is a notable unrecognized process in land-air CO_2_ exchange, particularly in areas of low biotic activity and large temperature fluctuations.

## RESULTS

### Negative soil carbon flux rates

The diffusional flux of CO_2_ across the soil-air interface (defined here as positive if the net flux is into the atmosphere or negative if the reverse) is driven by the concentration gradient, meaning that for positive fluxes, CO_2_ concentrations in soil are higher than those in the atmosphere (fig. S1). A substantial number of studies have identified negative nocturnal soil CO_2_ fluxes in desert soils, driven by soil CO_2_ concentrations below local tropospheric levels (Fig. 1 and data S1). The negative flux follows the pattern of the diurnal soil temperature cycle, reaching positive values during the warming phase, and becoming negative during the cooling phase. The negative flux rates typically range from −0.02 to −0.1 μmol m^−2^ s^−1^, reaching −1.5 to −1.64 μmol m^−2^ s^−1^ in some locations (Fig. 1 and data S1). Despite its widespread occurrence, the underlying cause of this anomaly remains uncertain (*13*–*16*). The annual integrated values of observed negative CO_2_ fluxes range from −4 to −65 g C m^−2^ year^−1^ (*13*, *14*), which approach the rates of biologically driven soil CO_2_ effluxes: 10^1^ to 10^3^ g C m^−2^ year^−1^ (*17*). While in situ soil CO_2_ production from autotrophic and heterotrophic soil respiration is almost always occurring, in dryland soils where biological production is limited by available C and/or water, its magnitude is smaller than the puzzling consumption processes, leading to net negative fluxes. There is no apparent biological process that should cause net consumption of CO_2_ under these well-aerated conditions. Chemoautotrophic carbon fixation may contribute to carbon cycling in soils (*18*, *19*), but its role in desert soil systems has not yet been evaluated (*20*), and it is uncertain why it would track inversely the diurnal temperature fluctuations. The unlikelihood of this mechanism is supported by several studies that have observed negative soil flux rates in sterilized soils (*21*–*23*). Thus, the prevailing explanations center on abiotic mechanisms: CO_2_ dissolution into soil water, calcite dissolution (*13*, *16*, *17*, *24*–*26*), and soil water vapor adsorption (*15*). Here, we address the hypothesis that thermally driven adsorption/desorption of CO_2_ onto mineral surfaces is the underlying mechanism for not only negative adsorption in low-respiration drylands but also for temperature-driven impacts on diurnal cycles of soil respiration in more mesic environments.

**Fig. 1.**
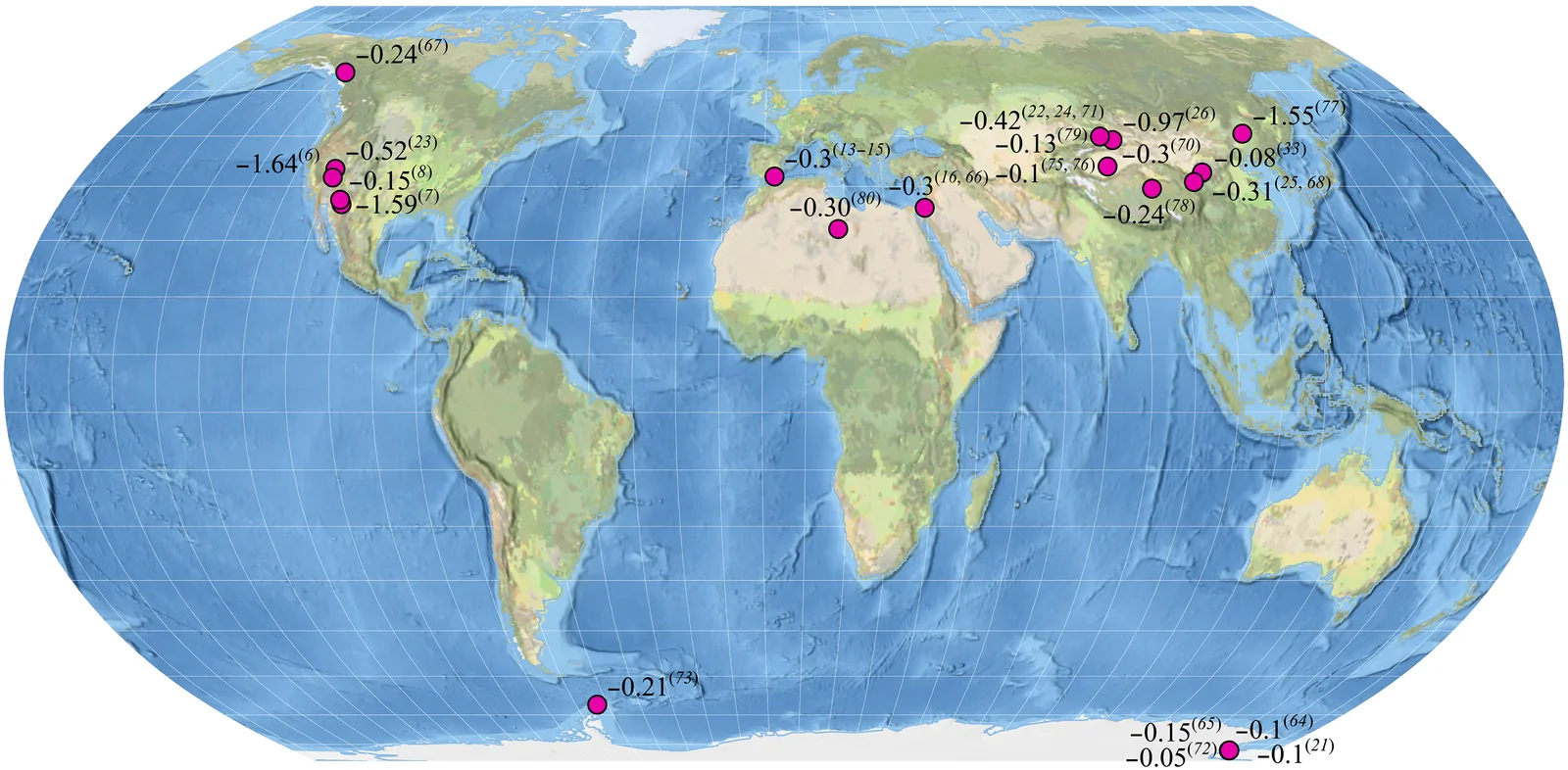
Reported negative nighttime soil surface CO_2_ fluxes across global sites. The soil surface CO_2_ flux rates (micromoles per square meter per second) are in large font, and the references for the observations are in parentheses (*6*, *7*, *8*, *13–16*, *21–26*, *33*, *64–80*). Values represent mean fluxes where summary statistics were available in the original publications (data S1); otherwise, individual reported negative nighttime flux values are shown. The map was created in ArcGIS Pro.

Our gaseous adsorption investigation was motivated by field observations. Between 2017 and 2019, we continuously monitored soil CO_2_ (fig. S2), temperature (*T*) (fig. S3), and volumetric water content (VWC) (θ) (fig. S4) in a characteristic low-elevation biotic zone (*Larrea tridentata* community) in the Mojave Desert National Preserve (*27*) (Materials and Methods). From the measured soil parameters (data S2) (and diffusion modeling described in Materials and Methods), net soil CO_2_ fluxes (fig. S5) were calculated over 10-minute intervals and aggregated to 1-hour intervals. During the hot, dry summer months, when net soil CO_2_ flux rates were low (−0.07 to 0.35 μmol m^−2^ s^−1^), negative surface fluxes were commonly observed at night (Fig. 2 and table S1). To test the hypothesis that this phenomenon could be explained, at least in part, by CO_2_ adsorption onto soil minerals, we subsequently measured CO_2_ adsorption on the same soil across the range of observed soil temperatures (2° to 70°C) and partial pressures of CO_2_ (*P*co_2_) with depth [0 to 0.5 kPa, corresponding to 0– to 5000–parts per million (ppm) CO_2_ levels] (Fig. 3A and figs. S6 to S8) (*28*). We also measured adsorption-desorption cycles and found minimal hysteresis (less than 4%), indicating that CO_2_ desorption is nearly equal to adsorption under diel soil CO_2_ conditions. Both processes occur rapidly, reaching equilibrium within seconds to minutes under typical soil conditions. Notably, after a rain event, negative nighttime soil CO_2_ fluxes often disappeared, replaced by pronounced CO_2_ pulses from soil to atmosphere (fig. S4), which are the result of moisture-driven increases in soil heterotrophic respiration and related phenomena (a.k.a. the Birch Effect) (*29*). In this study, we do not examine adsorption dynamics during or after rain events. Under high soil moisture, adsorption is likely negligible, as water occupies most available sorption sites and limits the soil’s capacity to adsorb CO_2_.

**Fig. 2.**
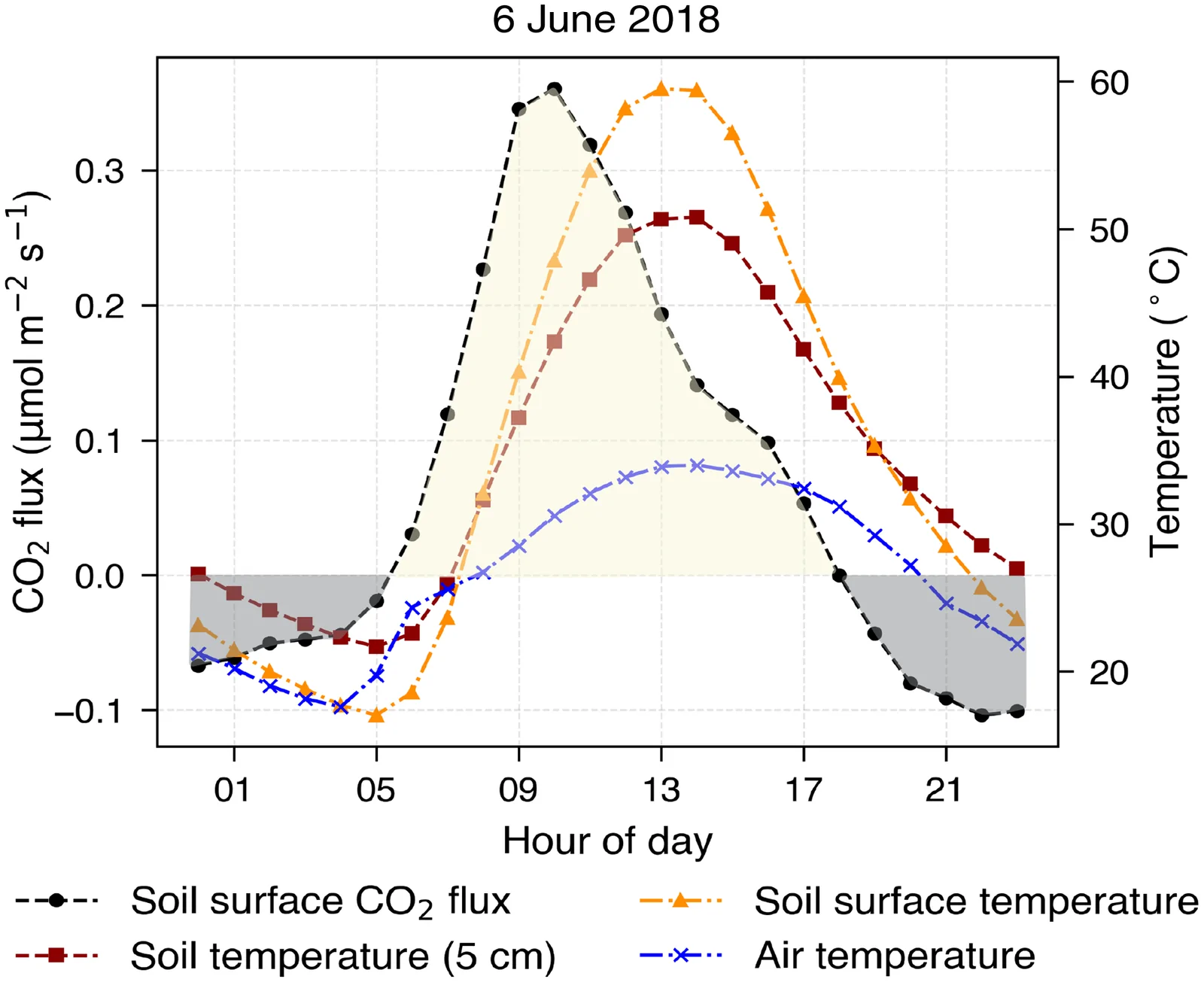
Diurnal variation in soil surface CO_2_ flux and temperature on 6 June 2018. Soil surface CO_2_ flux (left axis) is shown alongside air temperature, soil surface temperature, and soil temperature at 5-cm depth (right axis). Gray shading denotes periods of negative CO_2_ flux, and yellow shading highlights daytime hours. Data were recorded at 10-min intervals by meteorological and in situ soil sensors and aggregated to hourly means. Some components of this figure were created in BioRender. A, A. (2026) https://BioRender.com/fl1e6fc.

**Fig. 3.**
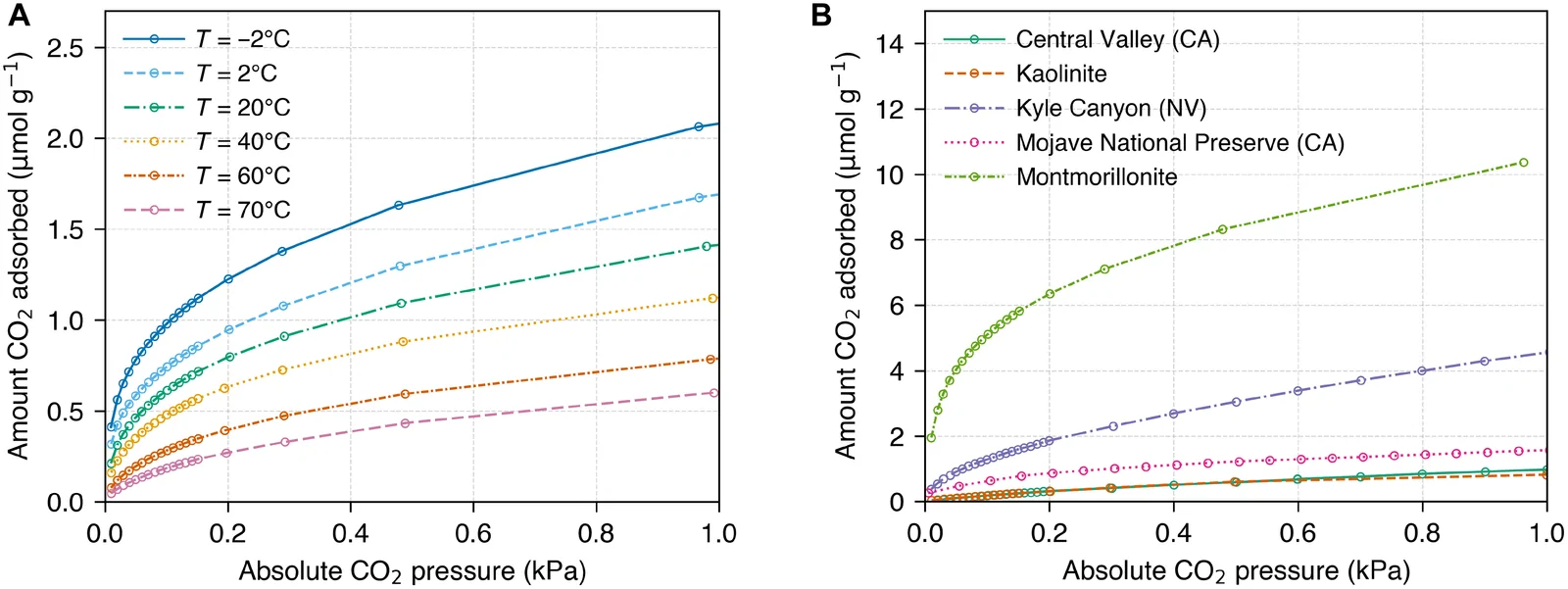
CO_2_ adsorption isotherms for several soils and clay minerals. (**A**) Temperature dependence of CO_2_ adsorption on Mojave Desert soil (Arizo soil series, Mojave National Preserve, CA) at temperatures ranging from −2° to 70°C. (**B**) Comparison of CO_2_ adsorption isotherms at ambient temperature (*T* = 20°C) for three soils—Snelling soil series (Central Valley, CA), carbonatic Typic Torriorthents (Kyle Canyon, NV), and Mojave Desert soil (Mojave National Preserve, CA)—and two clay minerals (kaolinite, KGa-1b, and montmorillonite, SWy-3). In both panels, CO_2_ adsorption (micromoles per gram) is plotted as a function of CO_2_ partial pressure (0 to 1 kPa, corresponding to 0- to 10,000-ppm CO_2_ levels). Some components of this figure were created in BioRender. A, A. (2026) https://BioRender.com/jzght33.

The Mojave soils (Mojave National Preserve, CA) formed in Late-Pleistocene granitic alluvium, classified as sandy-skeletal with minor pedogenic clay development, Typic Torriorthents (Arizo series), dominated by quartz (30%), Na/Ca feldspar (39.4%), K feldspar (23.3%), chlorite (5.1%), and borate (2%), with largely noncarbonate surface horizons (data S2). Despite the relatively low surface area per mass [Brunauer-Emmett-Teller (BET) surface area 3.3 to 6.5 m^2^ g^−1^], the soil adsorbs 1.2 μmol CO_2_ g^−1^ at partial pressures of 2000 ppm (equivalent to ∼0.2 kPa) and can adsorb-desorb 1 μmol CO_2_ g^−1^ across a 70°C temperature range (Fig. 3A and figs. S6 to S8) (*28*). In laboratory experiments, we observed that samples not subjected to heating and degassing adsorbed less CO_2_ than treated samples, which we expected, with the difference varying by CO_2_ partial pressure (fig. S9). At low pressures, adsorption on nondegassed samples was about ^1^/_10_ of the degassed values, while at higher pressures, it approached ^7^/_10_ of the degassed values. For our estimates of thermally driven adsorption-desorption in the field, we derived a nonlinear empirical relationship between the fully degassed (corresponding to the maximum CO_2_ adsorption capacity) and nondegassed (corresponding to natural occupancy of adsorption sites in soils) conditions (fig. S10). With these data, we applied a monotonic cubic Hermite interpolation (PCHIP) method to construct a three-dimensional (3D) matrix of CO_2_ adsorption as a function of both *T* and CO_2_ concentration (figs. S11 and S12 and data S3). This was combined with observed *T* and CO_2_ variations with depth (below), to calculate the total adsorption-desorption of the soil profile for a given time interval (Fig. 4).

**Fig. 4.**
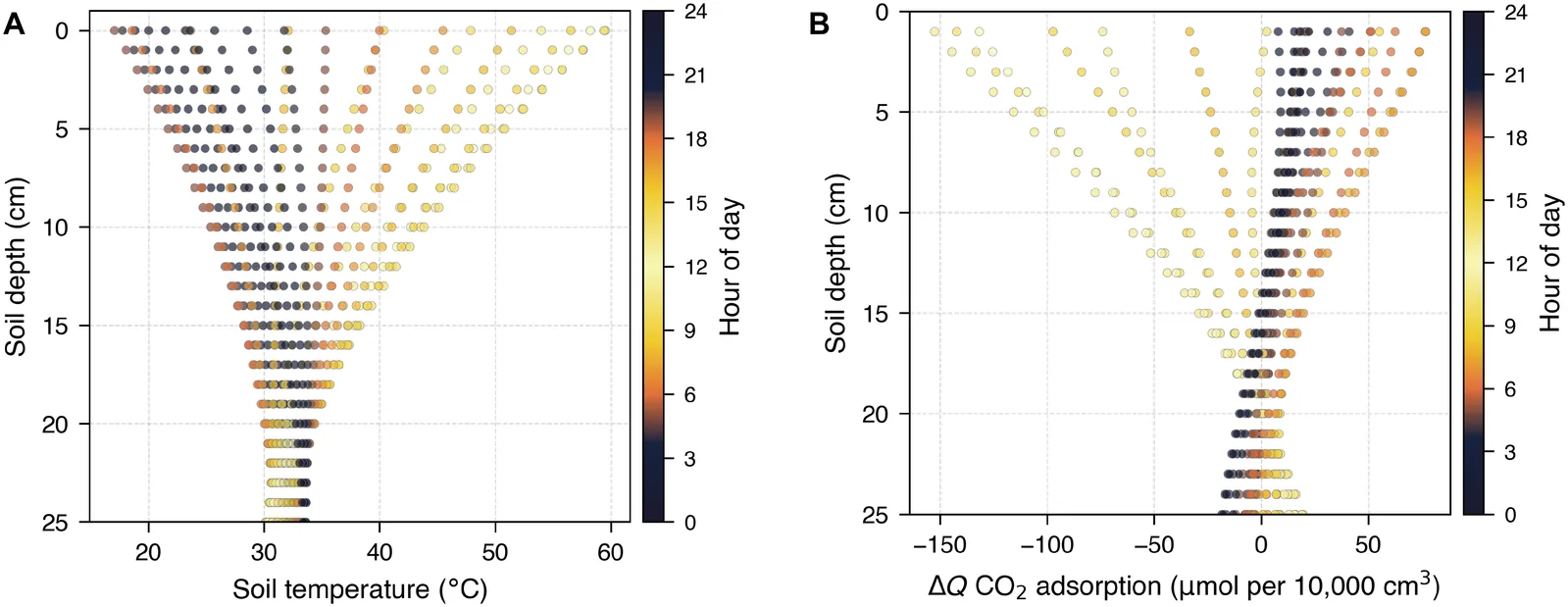
Depth profiles of soil temperature and CO_2_ adsorption change on 6 June 2018. (**A**) Soil temperature profiles were recorded at depths of 5 and 25 cm and then interpolated for each centimeter. (**B**) Hourly change in CO_2_ adsorption (Δ*Q*) at corresponding depths. Some components of this figure were created in BioRender. A, A. (2026) https://BioRender.com/bpzndt8.

To assess first-order variability in CO_2_ adsorption in different soils, we measured CO_2_ adsorption in several additional natural soils and reference clays at 20°C (Fig. 3B and Materials and Methods). The phyllosilicate clay montmorillonite has relatively high adsorption capacities, reaching 6 μmol CO_2_ g^−1^ at 0.2 kPa (corresponding to ∼2000-ppmv CO_2_), whereas kaolinite has low CO_2_ adsorption values. Soils vary in their adsorption capacities on the basis of their properties and environmental conditions. Soils with high carbonate content from Kyle Canyon near Las Vegas had adsorption values of 2 μmol CO_2_ g^−1^ at 0.2 kPa. The adsorption capacity of Mojave Desert soil lies at the lower end of the observed range among our tested soils and clays (1 μmol CO_2_ g^−1^ at 0.2 kPa). Thus, the quantities of thermally adsorbed CO_2_, which we calculate for this site, are conservative, and for other locations, the magnitude of diurnal adsorption/desorption may be much higher.

In arid regions, sparsely vegetated landscapes in the summer and/or dry periods experience a very large diurnal temperature range. At our Mojave site, diurnal amplitudes of ∼40°C are common (Fig. 2 and fig. S3). Soil temperature (due to heat flow) undergoes a diurnal pattern with depth and time of day. We used measured soil temperatures (5, 25, 50, and 125 cm) (fig. S3) and a cubic polynomial fitting to determine the depth at which diurnal temperature changes are effectively damped (Fig. 4A). We consider the layer above this depth to be the thermally active layer in the soil, and we replicated the observed quasi-periodical pattern of soil temperature on a daily cycle (Materials and Methods). Similarly, soil CO_2_ concentrations at any depth experience diurnal changes (fig. S2), and we fit depth-dependent models to the observed soil CO_2_ concentrations (fig. S13). From the temporal and depth patterns in both temperature and CO_2_ concentrations, we used a 3D surface matrix (fig. S11) to calculate depth-resolved *T* and CO_2_ concentration–driven changes in CO_2_ adsorption (Fig. 5A).

**Fig. 5.**
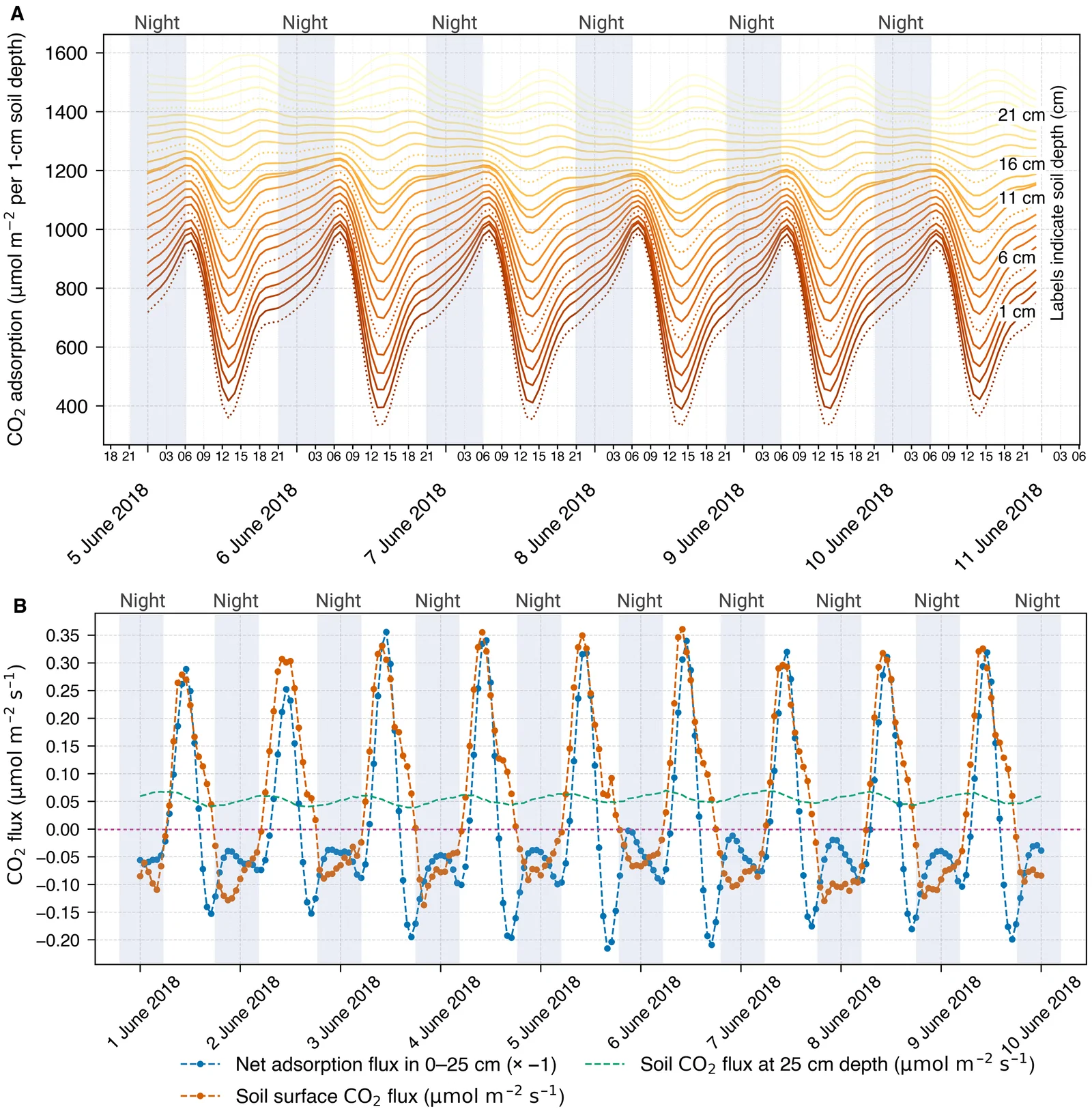
Diurnal dynamics of soil CO_2_ adsorption and fluxes. (**A**) Depth-resolved CO_2_ adsorption at 1-cm intervals across the 0- to 25-cm soil profile, expressed per unit soil area (1 m^2^) and per 1-cm soil depth. Labels indicate selected soil depths (centimeters); data are shown for 5 to 10 June 2018. (**B**) CO_2_ fluxes: (i) net CO_2_ adsorption flux integrated over the 0- to 25-cm depth [shown as × (−1) for visual comparison], (ii) soil surface CO_2_ flux, and (iii) soil CO_2_ flux at the 25-cm depth. All fluxes are in micromoles per square meter per second; data are shown for 1 to 10 June 2018. Some components of this figure were created in BioRender. A, A. (2026) https://BioRender.com/q68c760.

The calculated temporal changes in the quantity of CO_2_ adsorption/desorption varied greatly with depth because of the reduction in daily temperature ranges with depth (Fig. 4B). The calculated adsorbed CO_2_ at any given time of day was converted to rates of adsorption or desorption by evaluating depth-dependent changes over 1-hour time intervals (Fig. 5B).

There are important insights embedded in Fig. 5. First, there is a strong positive relationship between the observed soil surface CO_2_ flux and the calculated temperature-driven rates of physical adsorption/desorption between 0 and 25 cm. In this depth interval, rapid temperature fluctuations strongly influence CO_2_ adsorption and desorption onto and from soil particles. With increasing depth, the adsorption-desorption dynamics are damped, while the total amount of CO_2_ adsorbed increases because of higher *P*co_2_ concentrations. Second, the calculated physical adsorption-desorption rates, while inversely mirroring soil surface CO_2_ flux rates, at night (when negative fluxes occur) sometimes exceed the negative surface flux. This is because the net physical adsorption includes not only the inward flux of CO_2_ from the atmosphere into the soil (which has a lower CO_2_ concentration) but also the upward flux of CO_2_ from deeper depths into the upper 25 cm (fig. S14). When net adsorption minus the upward flux from deeper in the soil across the 25-cm interface is calculated, the difference is numerically very close to that of the observed negative soil surface CO_2_ flux.

We find strong evidence that physical adsorption drives the negative fluxes, and that, additionally, the daily desorption is a strong contributor to the positive soil CO_2_ fluxes that occur during the day, but with some interesting complexities. The diurnal cycle shows a clear phase shift between air and soil temperatures at various depths, as well as between CO_2_ concentrations at different depths. As a result, CO_2_ flux and net adsorption do not respond to temperature alone but reflect the combined, time-integrated effects of temperature and CO_2_ pressure. Consequently, adsorption represents a cumulative thermodynamic response to asynchronous variations in thermal and concentration gradients within the soil profile (fig. S15). Despite the complexity of integrating field observations with laboratory-obtained parameters, the agreement between adsorption and flux divergence is strong [coefficient of determination (*R*^2^) = 0.78] in dry hot months, for example, in August 2017, June 2018, and August 2019 (fig. S16).

Undoubtedly, thermally driven adsorption-desorption on soils and other solid surfaces is a global phenomenon, but its presence is obscured in soils with high biological CO_2_ production. In addition, soil relative humidity and water content likely affect CO_2_ adsorption capacity. However, studies of CO_2_ adsorption under environmentally relevant conditions, where both CO_2_ and water vapor are present, are experimentally difficult and, therefore, remain limited. Experiments on pure minerals have shown that increasing relative humidity reduces CO_2_ adsorption by competing for adsorption sites (*30*). In contrast, studies of polymer-based sorbents have reported enhanced CO_2_ adsorption under humid conditions due to water-induced pore opening and sorbent swelling (*31*).

We also considered the role of CO_2_ partitioning into soil water driven by temperature-dependent carbonic acid speciation and carbonate mineral dissolution/precipitation (fig. S17 and data S4). We found that because of very low soil water contents at the time negative fluxes are observed, the role of CO_2_ consumption by aqueous phase carbonate system reactions is small relative to the amount and rates calculated for physical adsorption. It was at least an order of magnitude lower than physical gaseous adsorption during the dry season (fig. S17). The relative impact of aqueous CO_2_ processes may be greater during different seasons of the year in the Mojave or in more mesic environments.

### Hysteresis of soil respiration

As in many studies (*3*, *7*, *13*, *32*), we observed a temperature-dependent diurnal hysteresis on soil respiration rates (Fig. 6A). The pattern is that soil respiration increases with increasing temperature, but the observed respiration rate during the morning warming phase is higher than at the same temperature during the evening cooling phase. A number of mechanisms have been proposed for this phenomenon, including lag times between measured temperature and the observed flux at the soil surface, temperature effects on diffusion-related parameters, and/or temperature (and time) lags in plant substrates available for respiration (*3*, *4*).

**Fig. 6.**
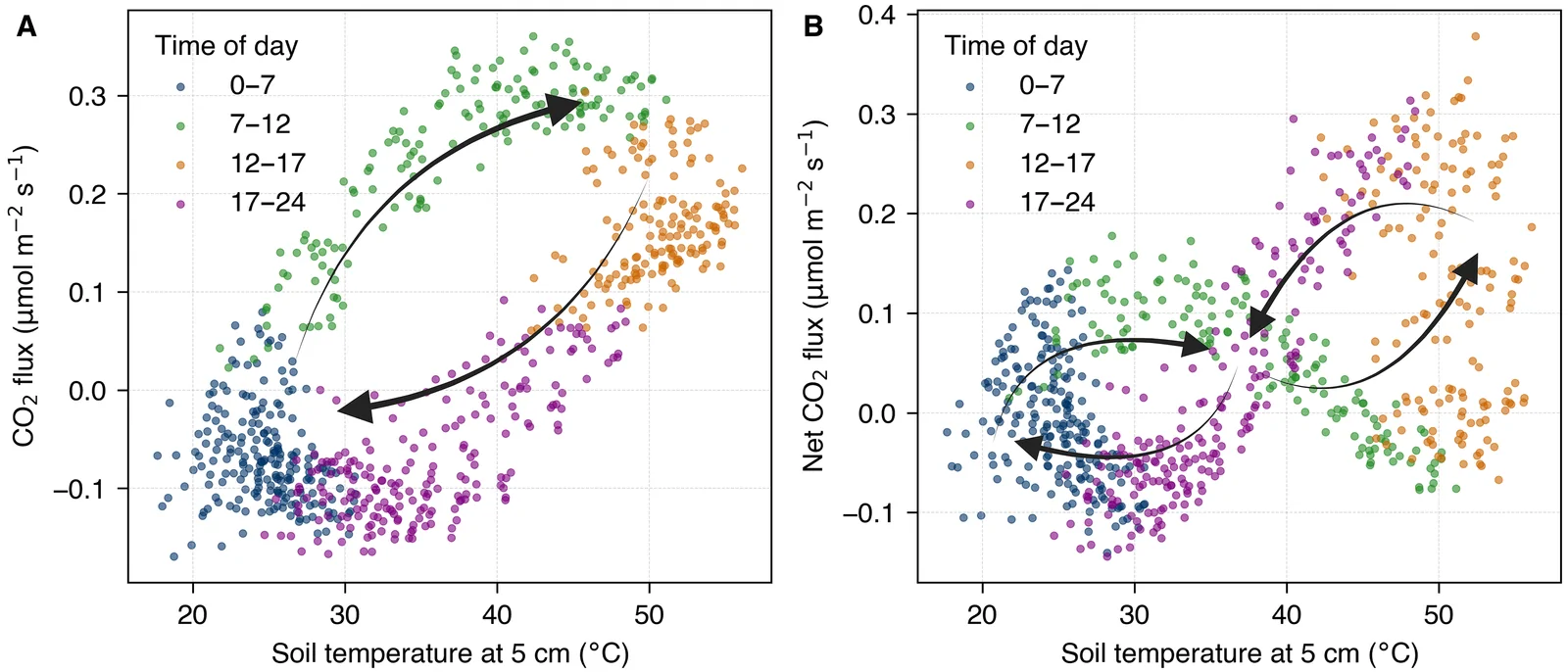
Diurnal hysteresis between soil surface CO_2_ flux and soil temperature at 5-cm depth during June 2018. (**A**) Soil surface CO_2_ flux as a function of soil temperature, showing clockwise hysteresis. (**B**) Net CO_2_ flux (surface flux minus adsorption flux) as a function of soil temperature, illustrating the contribution of CO_2_ adsorption-desorption to the observed hysteresis. Some components of this figure were created in BioRender. A, A. (2026) https://BioRender.com/uxrb375.

Here, we test the hypothesis that thermally driven adsorption-desorption is a contributor to this phenomenon. For the month of June 2018, we plot the soil respiration rates for all days for hourly intervals. The clockwise temporal trajectory of the respiration reflects the commonly observed pattern described above (*3*). We then subtracted from the fluxes the calculated net thermally driven adsorption-desorption for the time interval (Fig. 6B). Once we have subtracted physical adsorption/desorption from the June flux data, the CO_2_ flux data still maintain an apparent temperature dependence, but at higher temperatures the hysteresis becomes counterclockwise.

This comparison has several important implications. First, the observation of higher CO_2_ fluxes during the daily warming phase is frequently attributed to thermal acceleration of microbial respiration. However, this work indicates that a substantial contributor to this behavior is the thermal desorption of CO_2_ sorbed to soil particle surfaces during the previous night. Second, similarly, the apparent lower temperature sensitivity of respiration during the daily cooling phase could be explained by the physical adsorption of CO_2_ during this interval, which counters the biological flux. A third feature is that once the physical movement of CO_2_ is removed from the net fluxes, the hysteresis takes on a new, counterclockwise pattern. This suggests that respiration is not as sensitive to temperature during the morning warmup as it is during the evening cooldown. The adsorption-corrected pattern is consistent with recent research suggesting that plant respiration is substrate dependent (*2*) and that after a nighttime dark period, plants will be more deficient in respirable substrates than after the daytime photosynthetic period. Thus, from theory, plant respiration should follow the pattern of the adsorption-corrected flux patterns in Fig. 6B. Also, thermal convection may play a role in the observed hysteretic behavior (*33*).

## DISCUSSION

Thermally driven gaseous adsorption onto synthetic materials deployed in desert environments has been used for atmospheric water harvesting (*34*), but the adsorption of other gases onto natural soils has received little attention. Davidson *et al.* (*12*) estimated that adsorption contributes only marginally to CO_2_ exchange, leading Schlesinger (*35*) to conclude that it accounts for a minor fraction of the apparent CO_2_ sink. However, Davidson *et al.* (*12*) did not address the temperature-driven dynamics of adsorption, focusing instead on total amounts potentially adsorbed. Here, we focus on the magnitude of gross fluxes driven by diurnal temperature ranges. We used our calculated annual average values of the total amount of CO_2_ thermally adsorbed/desorbed per day from the upper 25 cm of the Mojave soil (16.7 to 19.3 g CO_2_-C m^−2^ year^−1^) and multiplied this by the global desert area (17.64 × 10^6^ km^2^) (*36*) and the ice-free land area of the planet (130 × 10^6^ km^2^) (*37*). This extrapolated estimate suggests that from 0.3–0.34 to 2.2–2.5 Gt CO_2_-C may be exchanged annually through purely physical processes across the land-air interface. Although our global estimate is very uncertain, it indicates that the daily amounts of CO_2_ adsorbed and released by surface soils are substantial, contributing substantially to CO_2_ cycling, particularly in arid ecosystems. This estimate is based on measurements from Mojave Desert soils, which represent the lower bound of the gaseous adsorption potential. Soils richer in organic matter, clay, and carbonates likely exhibit even greater adsorption capacity, highlighting the need for studies across diverse geographic areas. Furthermore, the addition of biochar and other adsorptive amendments to arid soils has been proposed as a nature-based strategy for enhancing carbon capture (*38*–*40*).

Desert ecosystems with strong daily temperature variability are likely to exhibit the most pronounced CO_2_ adsorption-desorption rates. Consequently, along a climatic gradient from dry to humid ecosystems, the magnitude of physical CO_2_ adsorption likely decreases while biological soil respiration generally increases. Under a warming climate, when the entire diurnal temperature cycle shifts toward warmer conditions (assuming the same day-night temperature amplitudes), CO_2_ adsorption capacity will decrease (fig. S12). However, the net effect of this on the annual atmospheric CO_2_ budget is near zero (assuming that the adsorbed CO_2_ is not involved in some subsequent chemical reaction).

In addition, CO_2_ adsorption may have impacts on the stable isotope composition of atmospheric CO_2_. Physical adsorption of CO_2_ onto mineral surfaces may place it in more direct contact with mineral-associated soil water and contribute to the well-studied effect of soil water on the stable O isotope composition of atmospheric CO_2_ (*41*, *42*). In addition, there are a few experiments that suggest that adsorbed CO_2_ on minerals may induce a unique C isotope effect on the remaining CO_2_, with the present data suggesting that ^12^CO_2_ may be preferentially adsorbed onto basalt (*43*) and onto clay (*44*) minerals. This process (whatever the sign of the isotope effect) may have impacts on the measured δ^13^C value of soil respiration over diurnal time courses. These potential isotope effects require much further study, but recognizing that physical processes occur in concert with biologically driven CO_2_ fluxes is important as land-air C exchange studies expand in complexity and spatial scope. Thermally driven atmospheric CO_2_ adsorption has long been considered an important process on Mars (*45*–*47*), and our study identifies it as a notable process in arid environments and likely globally.

## MATERIALS AND METHODS

### Laboratory CO_2_ (and N_2_) adsorption isotherm measurements

We conducted main CO_2_ adsorption isotherm experiments on soils from the Mojave Desert (CA), and additionally on soils from Kyle Canyon (NV) and the Central Valley (CA), and on the clay minerals Na-montmorillonite (SWy-3) and kaolinite (KGa-1b). The gas adsorption isotherms were measured using a three-port Micromeritics 3Flex gas adsorption analyzer via a manometric method. This instrument allowed us to measure at very low gas pressures and featured a high-sensitivity pressure transducer, enabling a measurement range from 1.3 × 10^−9^ to 1.0 P/P_0_. This capability allowed us to accurately replicate ambient atmospheric and soil CO_2_ partial pressures, spanning from 0.04 to 0.5 kPa (∼400- to 5000-ppmv CO_2_ partial pressure in soil air). For Mojave Desert soils, we conducted experiments at eight temperatures to obtain CO_2_ adsorption isotherms that reflect seasonal and diurnal soil surface changes: −2°, 2°, 5°, 10°, 20°, 40°, 60°, and 70°C. For other soils and clays, we obtained the isotherms at *T* = 20°C. The temperature was controlled and stabilized using a Micromeritics Iso-Controller unit. The full dataset is available in a Zenodo repository (*28*).

The average soil sample for the adsorption isotherm experiments was ∼3 to 4 g. Before measurement, each sample was purged with high-purity He or N_2_ gas (at 1-atm pressure) at 90°C for 1 hour, followed by at least 4 hours at 350°C. This process, performed with a Micromeritics Flow Prep 060 unit, ensured consistent background measurements.

To ensure that the sample preparation process (degassing and heating) did not change soil properties related to adsorption, we also performed thermogravimetric analysis (TGA) on the Mojave Desert soil using the METTLER TOLEDO Thermal Analysis System TGA/DSC ^3+^ under an inert Ar atmosphere. This analysis allowed us to measure the weight loss caused by heating. The initial sample weighed 78.1020 mg, and it was placed on a Platinum 70-μl holder with a mass of 278.4460 mg. Results showed a very low weight loss (∼2%), primarily due to water evaporation (fig. S18).

After degassing, the sample tubes were moved to the instrument analysis ports, and the analysis runs were conducted. The initial and final absolute pressures, equilibration intervals, and gas dosage amounts were set manually, on the basis of the soil CO_2_ partial pressure range identified during field observations. The typical starting pressure was about 0.001 kPa, and the equilibration interval at this pressure was 10 seconds. The amounts of gas absorbed are reported in millimoles per gram of soil; measurement details are included in the measurement reports (*28*).

We used the same set of soil samples to measure adsorption isotherms at various temperatures. Each experiment was performed in triplicate; however, only Port 2 had an advanced transducer capable of detecting CO_2_ adsorption at very low pressures (below 1.3 kPa). For Mojave Desert soils, we repeated the experiments at different temperatures to generate a second set of triplicates and assess measurement uncertainties. These replicates were conducted at four temperatures (*T* = −2°, 20°, 40°, and 60°C). The pressure points during adsorption measurements varied slightly across replicates, leading to nonaligned data. To compute the SD between replicates, we interpolated the adsorption values onto a common pressure grid using spline interpolation. We then calculated the mean, SD, and 95% confidence intervals (fig. S6, A to D, and data S5). The adsorption isotherms from replicates generally follow consistent patterns and are well aligned, though some variability in the absolute CO_2_ amounts was observed—likely because of soil heterogeneity. The soil surface areas ranged from 3.3 to 6.5 m^2^s.a. g^−1^, and this variation in sorption sites contributed to the spread in CO_2_ adsorption values among replicates (*28*).

For Mojave Desert soils, adsorption isotherms at the other four temperatures studied (*T* = 2°, 5°, 10°, and 70°C) were measured in triplicate, with no repeats (fig. S7, A to D). Consequently, only a single set of data was available at very low pressures, obtained with the advanced transducer (Port 2). Calculating average values above 1.3 kPa and extrapolating to lower pressures introduced minor distortions. Therefore, we relied on the high-resolution data from the advanced pressure transducer (Port 2) for these temperatures, as it offers the most precise measurements in the low-pressure range. The isotherms at *T* = 5° and 10°C exhibited unexpectedly higher adsorption than at −2°C, especially at low pressures (fig. S8). We repeated the adsorption analysis at *T* = −2°C, which confirmed strong reproducibility (fig. S6A), supporting the reliability of the −2°C isotherm. Because of physical inconsistencies, the isotherms at *T* = 5° and 10°C were excluded from further analysis. The final dataset included mean isotherm values for −2°, 20°, 40°, and 60°C, along with high-resolution data from the advanced transducer at 2° and 70°C.

To measure hysteresis behavior between adsorption and desorption at soil-relevant CO_2_ concentrations, we conducted an experiment with several adsorption-desorption subcycles across pressure ranges of 500 to 1500 ppm (0.05 to 0.15 kPa) and 5000 to 6500 ppm (0.5 to 0.6 kPa), then reached 10 kPa and repeated the first two pressure loops. The measured diurnal adsorption-desorption hysteresis was very small, ranging from ∼0.3 to 4%. After equilibration at 10-kPa CO_2,_ the diurnal hysteresis remained similarly low, at ∼0.3 to 3% of the adsorbed amount. In contrast, the comparison of the adsorption and desorption branches at 10-kPa CO_2_ showed a relatively larger hysteresis, ranging from 8 to 14%. However, such pressures substantially exceed those typically observed in the soils investigated in this study (fig. S19 and data S6). Therefore, we assume that at soil-relevant CO_2_ diurnal cycles, adsorption and desorption are symmetrical.

Adsorption time depends on the material’s surface complexity, specific surface area, and temperature and pressure conditions. The soil texture in the Mojave Desert is sandy loam, which has a relatively low surface area (BET surface area 3.3 to 6.5 m^2^ g^−1^). Consequently, adsorption is assumed to occur very quickly. On the basis of the adsorption isotherm measurements, we estimated that CO_2_ adsorption takes from seconds to minutes (fig. S20). Specifically, achieving adsorption equilibrium at up to 10 kPa at *T* = 2°C took around 100 min, indicating that the system reaches equilibrium within roughly 1 min for a pressure increase of 0.1 kPa (±1000-ppm CO_2_). This includes the adsorption process itself and equilibration-sensing measurements, which likely take up most of this time. The equilibration interval (the time over which pressure must remain stable within a very small range) was set to 10 s, and the pressure tolerance (how closely the actual pressure must match the target pressure to be acceptable) was below 0.01% of the weighted average. After each dose, the instrument took 11 pressure readings and calculated the weighted-average pressure drop to track the rate of change in pressure, which must be below 0.01% of the weighted average to make a measurement. CO_2_ adsorption rates decrease at lower temperatures, although the total amount adsorbed is greater. The instrument finished the adsorption isotherm measurement at *T* = 60°C more quickly than at *T* = 2°C; however, the time intervals were quite similar (fig. S20). The comparison of the adsorption and desorption time increments showed that they were approximately equal.

Adsorption under laboratory conditions (*Q_lab*) was measured for samples degassed to remove water vapor and other gases from the absorbent surfaces. To investigate the natural occupancy of adsorption sites in soils (*Q_nat*), we performed a series of isotherm measurements on nondegassed samples equilibrated at various relative humidity levels, controlled by saturated salt solutions of lithium chloride (LiCl for ∼15% RH) and potassium chloride (KCl for ∼86% RH), following the method in (*48*), and on uncontrolled samples stored under ambient laboratory conditions (*28*). In addition, adsorption isotherms were measured for soil samples heated and nitrogen purged at 150°C to partially degas and 350°C to fully degas, thereby removing most of the adsorbed water vapor. The adsorption capacities of the nondegassed soils were compared with measurements on degassed samples (fig. S9). This relationship was numerically represented by a transformation equation (*Q_nat* = 452.06 × *Q_lab*^1.9644) (fig. S10).

### Site description and soil sampling

The field site (geographic coordinates: 35.10613, −115.55074; elevation, 935 m) is situated in the Mojave National Preserve in California, within a creosote shrubland ecosystem typical of low-elevation Mojave Desert environments. The soil sampling and site were obtained under the National Park Service Research Permit MOJA-2024-SCI-0034. The climate is arid: The mean annual temperature is ∼18.3°C, and the mean annual precipitation is ∼156 mm (*49*). The vegetation composition at the site is dominated by creosote (*L. tridentata*) and burroweed (*Ambrosia dumosa*) shrubs (*50*). The soils have formed in Late-Pleistocene granitic alluvium (*51*) and are mapped as the Arizo series, a sandy-skeletal, mixed, thermic Typic Torriorthents with modest carbonate contents and minor pedogenic clay accumulation. Two soil profiles were excavated to a depth of 125 cm. The first soil profile was located on a patch of intercanopy bare ground; the second was under the canopy of a creosote (*Larrea tridentata*) bush (*50*). Soils were sampled at the surface and at four depths (5, 25, 50, and 125 cm) for laboratory analyses, and instrumentation was installed at the same depths to continuously monitor soil conditions (fig. S21).

In addition, for gas adsorption isotherm analysis, we used archived soil samples from Kyle Canyon (NV) and the Central Valley (CA), as well as common clay minerals, including well-crystallized kaolin (GA) (KGa-1b) (*52*) and Na-montmorillonite (WY) (SWy-3), purchased from the Clay Minerals Society. Kyle Canyon carbonate soils (NV) developed on a limestone-dominated alluvial fan, where 90 to 95% of clasts are derived from Paleozoic-Mesozoic limestones and dolomites. These sandy-skeletal, carbonatic Typic Torriorthents contain 10 to 25% CaCO_3_ in the A horizon (*53*). The Snelling soil (Central Valley, CA) is a noncalcareous soil from granitic Sierra Nevada glacial outwash (∼200 thousand years ago). The A horizon contains moderate clay (8 to 10%), near-neutral pH (6.7), and low organic matter (∼0.75%) (*54*). Na-montmorillonite (SWy-3) is a pure smectite clay mineral, characterized by a 2:1 phyllosilicate structure, high cation exchange capacity (76.4 mequiv 100 g^−1^), and elevated surface area (31.8 m^2^ g^−1^). Kaolinite (KGa-1b) is a 1:1 phyllosilicate, with a low cation exchange capacity (2.0 mequiv 100 g^−1^) and a modest surface area (10.05 m^2^ g^−1^).

### Field measurements

At the Mojave National Preserve site, each soil profile was equipped with in situ sensors to measure temperature, VWC, and electrical conductivity (CS615, Campbell Scientific, Logan, UT, USA) at four depths (fig. S21). The sensors were installed directly into the exposed wall of the soil excavation before back-filling. Soil CO_2_ sensors were also placed at the same depths. A cylindrical cavity was dug 20 cm into the exposed pit face to hold a 1–inch (2.54-cm)–diameter PVC tube, which was connected to a vertical PVC tube running to the soil surface via a 90° elbow. The pits were then refilled with soil, and solid-state soil CO_2_ sensors (GMP 252, Vaisala Inc., Vantaa, Finland) were lowered into the PVC elbow. The vertical PVC tubing was capped and sealed with silicone sealant above the soil surface. The VWC sensors detected changes in soil dielectric permittivity, mainly affected by water content. However, water’s permittivity depends on temperature, making VWC measurements sensitive to temperature. Following (*55*), we used periods with minimal VWC change to estimate the temperature sensitivity (*dVWC/dT*) on the basis of diurnal fluctuations in temperature and VWC, then corrected the data by subtracting *dVWC/dT*(*VWC*) *×* (*T − 25*) from the raw VWC measurements. Meteorological and in situ soil sensor data were recorded at 10-min intervals using a Campbell Scientific data logger (CR1000, Campbell Scientific Inc., Logan, UT, USA). The full dataset is available on a HydroShare repository (*27*).

### Determining the thermally active soil layer

In desert environments, large daily fluctuations in air temperature result in large daily temperature changes in shallow soil depths. The soil temperature record shows that this effect is strongest at soil depths above 20 to 25 cm (Fig. 4A). Below 20 to 25 cm, the temperature remains stable and does not fluctuate with day/night cycles. Therefore, we defined the thermally active layer as the soil layer above 25 cm, which we used as the lower boundary for studying adsorption depth profiles, with the upper boundary at the land surface (fig. S22).

### Soil analysis

Soil samples were dried in a 50°C oven and sieved to separate the gravel-sized fraction from the <2-mm-size fraction. Soil physical properties—gravimetric water content, percent gravel by weight, and bulk density—were measured following published methods (*56*) for both profiles. Particle size distribution and bulk mineralogy were analyzed using powder x-ray diffraction (*57*). The pH was determined with a saturated paste method using water at the UC Davis Analytical Laboratory. Organic carbon content was measured through continuous-flow dual-isotope analysis with a CHNOS Elemental Analyzer connected to an IsoPrime 100 mass spectrometer at the Center for Stable Isotope Biogeochemistry at UC Berkeley. The full dataset is available in the Supplementary Materials (data S2).

### Calculation of CO_2_ flux in soil

Soil CO_2_ fluxes across different depths, including soil respiration—the flux of CO_2_ across the soil-air interface (fig. S1), were calculated using the flux gradient method, making the explicit assumption that CO_2_ production (and consumption, where it occurs) and transport via molecular diffusion are the dominant processes controlling CO_2_ concentrations in the soil profile (*58*, *59*). Soil CO_2_ diffusivity, *D*_s_ (square meters per second), was calculated at each depth as a function of soil texture, temperature, pressure, and water content, following (*60*). The CO_2_ flux across various layers in the soil profile, including the surface, was then calculated using flux gradient theory and Fick’s first law of diffusion

$$F_{\left(z\right)}=-D_{s}\;\frac{\partial C}{\partial z}$$ (1)

where *F* is the soil CO_2_ flux (micromoles per square meter per second), *Ds* is soil CO_2_ diffusivity (square meters per second), *z* is soil depth (0, 5, 25, 50, and 125 cm), and *C* is the molar CO_2_ concentration (micromoles per cubic meter).

$$Ds=\mathrm{Da}\;\left(\phi^{2}\right){\left(\frac{\varepsilon }{\phi }\right)}^{\beta \times F_{\mathrm{cp}}}$$ (2)

where ϕ is the porosity, ε is the air-filled porosity (ϕ−VWC), β is an empirically fitted parameter taken to be 2.9, $F_{\mathrm{cp}}$ is the sand and silt content of the <2-mm-size fraction $\left(0\leq F_{\mathrm{cp}}\leq 1\right)$, and $D_{a}$ is the diffusivity of the CO_2_ in the air, corrected for temperature and pressure

$$D_{a}=D_{a0}\;{\left(\frac{T}{293.15}\right)}^{1.75.}\;\left(\frac{1.013\times 10^{5}}{P}\right)$$ (3)

where $D_{a0}$ is 1.47 × 10^−5^ m^2^ s^−1^ (*60*), *T* is the temperature, measured in kelvins, and *P* is the pressure in pascal.

### Calculation of soil CO_2_ flux divergence

Soil CO_2_ flux divergence, FD (figs. S1 and S14), in the entire thermally active soil layer (in the 0 to 25 cm) (not normalized to horizon thickness) was calculated from the difference between the CO_2_ fluxes across the 25-cm layer and across the soil-air interface (*z* = 0 cm) (*61*)

$$\text{FD}=F_{\left(z=25\;\text{cm}\right)}-F_{\left(z=o\;\text{cm}\right)}$$ (4)

### Calculation of the mass balance model of net CO_2_ adsorption/desorption in soil

To understand how CO_2_ adsorption corresponds to soil CO_2_ fluxes, we formulated and applied a mass balance model. Our experimental results showed that adsorption is a very temperature-sensitive process. As the temperature drops, the amount of CO_2_ adsorption onto the soil particles increases. We can define the rate of CO_2_ adsorbed/desorbed by a soil volume of a thermally active layer as the difference of CO_2_ adsorbed amounts between two observation time points [*t_n_*, *t*_(*n*−1)_]

$$\Delta Q_{d}^{t_{n},t_{n-1}}=Q_{d}^{t_{n}}-Q_{d}^{t_{n-1}}$$ (5)

The net CO_2_ adsorbed or desorbed in the thermally active soil layer per time increment is equal to the CO_2_ flux divergence

$$\Delta Q^{t_{n},t_{n-1}}\approx \left(F_{\text{in}}-F_{\text{out}}\right)\times \Delta t$$ (6)

where ΔQ is the amount of adsorbed/desorbed CO_2_ in the thermally active soil layer per time increment,$F_{\text{in}}$ is soil-atmosphere CO_2_ flux (micromoles per square meter per second), calculated using the gradient method in the 0- to 5-cm soil layer, $F_{\text{out}}$ is soil CO_2_ flux soil layer (micromoles per square meter per second), calculated using the gradient method in the 25- to 50-cm soil layer, and ∆t is the time increment (for example, 1 hour).

### Calculation of the dissolution of CO_2_ into soil water

The amount of dissolved inorganic carbon (DIC) in the soil water at each 1-hour time step was determined through carbonic acid system speciation calculations (*62*). For these calculations, temperature, CO_2_ partial pressure, and VWC were measured at 5- and 25-cm soil depths and then interpolated to 1-cm depth. Measurements were collected at a 10-min time resolution and averaged to a 1-hour resolution. We measured the soil pH at each soil horizon in the studied soil profile and then interpolated it at 1-cm intervals. The soil pH changed from 7.2 in the topsoil to 8.4 at a depth of 25 cm. We assumed that pH was at a steady state.

The carbonate chemistry is as follows

$${\text{CO}}_{2}\left(g\right)\;k_{H}↔CO_{2}\left(\text{aq}\right)$$ (7)

where $k_{H}$ is the Henry equilibrium coefficient and ${\text{CO}}_{2}\left(g\right)$ is the partial pressure of CO_2._CO_2_ (aq) represents the combination of dissolved CO_2_ and H_2_CO_3_

$${\text{CO}}_{2}\left(\text{aq}\right)\;k_{1}↔H^{+}\left(\text{aq}\right)+\text{HC}{O_{3}}^{-}\left(\text{aq}\right)$$ (8)

$$\text{HC}{O_{3}}^{-}\left(\text{aq}\right)\;k_{2}↔H^{+}\left(\text{aq}\right)+{{\text{CO}}_{3}}^{2-}\left(\text{aq}\right)$$ (9)

For the calculations, we applied the Henry’s law constant ( *K*_H_) and equilibrium constants (*K*_1_ and *K*_2_), which were calculated according to (*63*)

$$\begin{array}{c} \text{log }K_{H}=108.3865+0.01985076\times \\ T-\frac{6919.53}{T}-40.45154\times \text{log}\;T+\frac{669365}{T^{2}} \end{array}$$ (10)

$$\begin{array}{c} \text{log}\;K_{1}=-356.3094-0.06091964\times T\\ +\frac{21834.37}{T}+126.8339\times \text{log}\;T-\frac{1684915}{T^{2}} \end{array}$$ (11)

$$\begin{array}{c} \text{log}\;K_{2}=-107.8871-0.03252849\times T\\ +\frac{5151.79}{T}+38.92561\times \text{log}\;T-\frac{563713.9}{T^{2}} \end{array}$$ (12)

The total DIC is the sum of the concentrations of the carbonate ions (*62*)

$$\text{DIC}=\text{pC}O_{2}*K_{H}*\left(1+\frac{K_{1}}{\left[H^{+}\right]}+\frac{K_{1}\times K_{2}}{{\left[H^{+}\right]}^{2}}\right)$$ (13)

where *P*co_2_ is the partial pressure of CO_2_ in equilibrium with the solution (atmosphere), *K*_H_ is the Henry equilibrium coefficient, *K*_1_ and *K*_2_ are the first and second dissociation constants of carbonic acids, and [H^+^] is the hydrogen ion concentration (moles per liter). These calculations are available in the Supplementary Materials (data S4).

### Data processing

Laboratory-derived adsorption data were compiled into a standardized format to create a temperature- and pressure-dependent CO_2_ adsorption matrix (fig. S11). The calculated adsorption values were interpolated across a regular grid using monotonic cubic Hermite interpolation (PCHIP) to construct a 2D adsorption surface, *Q* (*T*, *P*), for each temperature-pressure pair. The interpolation was carried out in two steps: First, CO_2_ adsorption (*Q*) was interpolated across pressure steps (1-Pa resolution) for each temperature; second, the resulting interpolated adsorption values were used to generate a temperature-smoothed matrix at 1-K intervals from 271 to 343 K. A 3D wireframe plot of the adsorption matrix was created to visualize trends across the full temperature and pressure space (fig. S11). This dataset serves as input for further modeling (data S3).

To characterize vertical soil temperature and CO_2_ concentration profiles at high temporal resolution, the field-measured temperature and *P*co_2_ data were first aggregated to hourly means to reduce noise while preserving natural variability. For each timestamp, temperature values measured at discrete soil depths (5, 25, 50, and 125 cm) were used to fit a cubic polynomial curve. The fitted curve was then used to interpolate continuous temperature estimates with soil depths at 1-cm intervals, generating synthetic soil temperature profiles.

The laboratory-derived adsorption *Q* (*T*, *P*) matrix was used to determine the amount of CO_2_ adsorbed (*Q*, millimoles per gram) within the vertical soil profiles. For each hourly timestamp in the field dataset, CO_2_ concentrations and temperatures at the soil surface and at depth intervals were used as inputs to a bilinear interpolation function, which returned the corresponding adsorption value from the laboratory matrix.

The resulting gravimetric *Q* values (millimoles per gram) were further converted to volumetric units (mmol 10,000 cm^−3^) using depth-resolved soil bulk density and gravel content obtained from core samples. Gravel corrections accounted for the reduced adsorption volume based on the known gravel density (2.6 g cm^−3^). Final volumetric CO_2_ adsorption profiles were generated for each site and depth interval.

## Supplementary Materials

### The PDF file includes:

Figs. S1 to S22 Table S1 Legends for data S1 to S6 References

## References

[1] B. Bond-Lamberty, A. Ballantyne, E. Berryman, E. Fluet-Chouinard, J. Jian, K. A. Morris, A. Rey, R. Vargas, Twenty years of progress, challenges, and opportunities in measuring and understanding soil respiration. J.Geophys.Res. Biogeosci. 129, e2023JG007637 (2024).

[2] S. Hashimoto, A. Ito, K. Nishina, Divergent data-driven estimates of global soil respiration. Commun. Earth Environ. 4, 460 (2023).

[3] W. Song, S. Chen, Y. Zhou, B. Wu, Y. Zhu, Q. Lu, G. Lin, Contrasting diel hysteresis between soil autotrophic and heterotrophic respiration in a desert ecosystem under different rainfall scenarios. Sci. Rep. 5, 16779 (2015). 10.1038/srep16779 PMC4663751 26615895

[4] Y. Dusza, E. P. Sanchez-Cañete, J.-F. L. Galliard, R. Ferrière, S. Chollet, F. Massol, A. Hansart, S. Juarez, K. Dontsova, J. van Haren, Biotic soil-plant interaction processes explain most of hysteretic soil CO_2_ efflux response to temperature in cross-factorial mesocosm experiment. Sci. Rep. 10, 905 (2020). 10.1038/s41598-019-55390-6 PMC6976568 31969580

[5] Q. Zhang, G. G. Katul, R. Oren, E. Daly, S. Manzoni, D. Yang, The hysteresis response of soil CO_2_ concentration and soil respiration to soil temperature. J. Geophys. Res. Biogeo. 120, 1605–1618 (2015).

[6] G. Wohlfahrt, L. F. Fenstermaker, J. A. Arnone Iii, Large annual net ecosystem CO_2_ uptake of a Mojave Desert ecosystem. Glob. Chang. Biol. 14, 1475–1487 (2008).

[7] E. P. Hamerlynck, R. L. Scott, E. P. Sánchez-Cañete, G. A. Barron-Gafford, Nocturnal soil CO_2_ uptake and its relationship to subsurface soil and ecosystem carbon fluxes in a Chihuahuan Desert shrubland. J. Geophys. Res. Biogeo. 118, 1593–1603 (2013).

[8] A. Cueva, T. H. M. Volkmann, J. van Haren, P. A. Troch, L. K. Meredith, Reconciling negative soil CO_2_ fluxes: Insights from a large-scale experimental hillslope. Soil Syst. 3, 10 (2019).

[9] I. Langmuir, The adsorption of gases on plane surfaces of glass, mica and platinum. J. Am. Chem. Soc. 40, 1361–1403 (1918).

[10] K. Kaiser, G. Guggenberger, Mineral surfaces and soil organic matter. Eur. J. Soil Sci. 54, 219–236 (2003).

[11] I. C. Bourg, L. E. Beckingham, D. J. DePaolo, The nanoscale basis of CO_2_ trapping for geologic storage. Environ. Sci. Technol. 49, 10265–10284 (2015). 10.1021/acs.est.5b03003 26266820

[12] G. R. Davidson, A. Phillips-Housley, M. T. Stevens, Soil-zone adsorption of atmospheric CO_2_ as a terrestrial carbon sink. Geochim. Cosmochim. Acta 106, 44–50 (2013).

[13] M. Kim, C. Lopez-Canfin, R. Lázaro, E. P. Sánchez-Cañete, B. Weber, Unravelling the main mechanism responsible for nocturnal CO_2_ uptake by dryland soils. Sci. Total Environ. 926, 171751 (2024). 10.1016/j.scitotenv.2024.171751 38503391

[14] C. Lopez-Canfin, R. Lázaro, E. P. Sánchez-Cañete, Disparate responses of soil-atmosphere CO_2_ exchange to biophysical and geochemical factors over a biocrust ecological succession in the Tabernas Desert. Geoderma 425, 116067 (2022).

[15] C. Lopez-Canfin, R. Lázaro, E. P. Sánchez-Cañete, Water vapor adsorption by dry soils: A potential link between the water and carbon cycles. Sci. Total Environ. 824, 153746 (2022). 10.1016/j.scitotenv.2022.153746 35150687

[16] N. Sagi, M. Zaguri, D. Hawlena, Soil CO_2_ influx in drylands: A conceptual framework and empirical examination. Soil Biol. Biochem. 156, 108209 (2021).

[17] J. W. Raich, W. H. Schlesinger, The global carbon dioxide flux in soil respiration and its relationship to vegetation and climate. Tellus B 44, 81–99 (1992).

[18] C. León-Sobrino, J.-B. Ramond, G. Maggs-Kölling, D. A. Cowan, Nutrient acquisition, rather than stress response over diel cycles, drives microbial transcription in a hyper-arid Namib Desert soil. Front. Microbiol. 10, 1054 (2019). 10.3389/fmicb.2019.01054 PMC6527771 31139170

[19] M. Spohn, K. Müller, C. Höschen, C. W. Mueller, S. Marhan, Dark microbial CO_2_ fixation in temperate forest soils increases with CO_2_ concentration. Glob. Chang. Biol. 26, 1926–1935 (2020). 10.1111/gcb.14937 31774225

[20] Z. Liu, Y. Sun, Y. Zhang, W. Feng, Z. Lai, S. Qin, Soil microbes transform inorganic carbon into organic carbon by dark fixation pathways in desert soil. JGR Biogeosci. 126, e2020JG006047 (2021).

[21] A. N. Parsons, J. E. Barrett, D. H. Wall, R. A. Virginia, Soil carbon dioxide flux in Antarctic dry valley ecosystems. Ecosystems 7, 286–295 (2004).

[22] J. Xie, Y. Li, C. Zhai, C. Li, Z. Lan, CO_2_ absorption by alkaline soils and its implication to the global carbon cycle. Environ. Geol. 56, 953–961 (2009).

[23] E. L. Yates, A. M. Detweiler, L. T. Iraci, B. M. Bebout, C. P. McKay, K. Schiro, E. J. Sheffner, C. A. Kelley, J. M. Tadić, M. Loewenstein, Assessing the role of alkaline soils on the carbon cycle at a playa site. Environ. Earth Sci. 70, 1047–1056 (2013).

[24] J. Ma, Z.-Y. Wang, B. A. Stevenson, X.-J. Zheng, Y. Li, An inorganic CO_2_ diffusion and dissolution process explains negative CO_2_ fluxes in saline/alkaline soils. Sci. Rep. 3, 2025 (2013). 10.1038/srep02025 PMC3685845 23778238

[25] K. Fa, Z. Liu, Y. Zhang, S. Qin, B. Wu, J. Liu, Abiotic carbonate dissolution traps carbon in a semiarid desert. Sci. Rep. 6, 23570 (2016). 10.1038/srep23570 PMC4810371 27020762

[26] Z.-Y. Wang, J.-B. Xie, Y.-G. Wang, Y. Li, Biotic and abiotic contribution to diurnal soil CO_2_ fluxes from saline/alkaline soils. Sci. Rep. 10, 5396 (2020). 10.1038/s41598-020-62209-2 PMC7096481 32214162

[27] G. E. Maurer, L. N. Lammers, R. G. Amundson, MojaveCarbon Climosequence, HYDROSHARE (2019); www.hydroshare.org/resource/d01662d827f34170a5fd3589e468d06b.

[28] A. Abramova, M. Getenet, G. Benjamin, R. Amundson, Gas Adsorption Isotherms and BET Surface Area Data for the Mojave Desert Soils and Clays Minerals, Zenodo (2026); 10.5281/zenodo.19226405.

[29] F. E. Moyano, S. Manzoni, C. Chenu, Responses of soil heterotrophic respiration to moisture availability: An exploration of processes and models. Soil Biol. Biochem. 59, 72–85 (2013).

[30] C. T. Johnston, T. S. Zwier, T. A. Ho, G. Xu, Y. Wang, S. B. Rempe, N. Dasgupta, M. Mills, R. Welsh, D. L. Bish, Competitive sorption of H_2_ O and CO_2_ in clay mineral interlayers. J. Phys. Chem. C 129, 7832–7845 (2025).

[31] A.-Y. Song, J. Young, J. Wang, S. N. Fricke, K. Piscina, R. Giovine, S. Garcia, M. van der Spek, J. A. Reimer, Discerning molecular-level CO_2_ adsorption behavior in amine-modified sorbents within a controlled CO_2_/H_2_O environment towards direct air capture. J. Mater. Chem. A 12, 25875–25886 (2024).

[32] C. Guan, N. Chen, L. Qiao, C. Zhao, Photosynthesis regulates the diel hysteresis pattern between soil respiration and soil temperature in a steppe grassland. Geoderma 408, 115561 (2022).

[33] Y. Gao, Z. Zhao, Y. Zhang, J. Liu, Response of abiotic soil CO_2_ flux to the difference in air-soil temperature in a desert. Sci. Total Environ. 785, 147377 (2021). 10.1016/j.scitotenv.2021.147377 33932680

[34] H. Kim, S. R. Rao, E. A. Kapustin, L. Zhao, S. Yang, O. M. Yaghi, E. N. Wang, Adsorption-based atmospheric water harvesting device for arid climates. Nat. Commun. 9, 1191 (2018). 10.1038/s41467-018-03162-7 PMC5864962 29568033

[35] W. H. Schlesinger, An evaluation of abiotic carbon sinks in deserts. Glob. Chang. Biol. 23, 25–27 (2017). 10.1111/gcb.13336 27135436

[36] Y. Chen, H. Lu, H. Wu, J. Wang, N. Lyu, Global desert variation under climatic impact during 1982–2020. Sci. China Earth Sci. 66, 1062–1071 (2023).

[37] P. R. Shukla, J. Skeg, E. C. Buendia, V. Masson-Delmotte, H.-O. Pörtner, D. C. Roberts, P. Zhai, R. Slade, S. Connors, S. Van Diemen, “Climate Change and Land: An IPCC special report on climate change, desertification, land degradation, sustainable land management, food security, and greenhouse gas fluxes in terrestrial ecosystems” (IPCC, 2019).

[38] A. J. Ringsby, C. M. Ross, K. Maher, Sorption of soil carbon dioxide by biochar and engineered porous carbons. Environ. Sci. Technol. 58, 8313–8325 (2024). 10.1021/acs.est.4c02015 PMC11097398 38689207

[39] X. Gui, X. Xu, Z. Zhang, L. Hu, W. Huang, L. Zhao, X. Cao, Biochar-amended soil can further sorb atmospheric CO_2_ for more carbon sequestration. Commun. Earth Environ. 6, 5 (2025).

[40] S. Helmrich, A. J. Ringsby, K. Maher, Reactive transport simulation of organic and inorganic carbon cycling following carbon dioxide sorption onto soil amendments in drylands. Front. Clim. 7, 1505472 (2025).

[41] R. Hesterberg, U. Siegenthaler, Production and stable isotopic composition of CO_2_ in a soil near Bern, Switzerland. Tellus B 43, 197–205 (1991).

[42] G. D. Farquhar, J. Lloyd, J. A. Taylor, L. B. Flanagan, J. P. Syvertsen, K. T. Hubick, S. C. Wong, J. R. Ehleringer, Vegetation effects on the isotope composition of oxygen in atmospheric CO_2_. Nature 363, 439–443 (1993).

[43] T. Rahn, J. M. Eiler, Experimental constraints on the fractionation of ^13^C/^12^C and ^18^O/^16^O ratios due to adsorption of CO_2_ on mineral substrates at conditions relevant to the surface of Mars. Geochim. Cosmochim. Acta 65, 839–846 (2001).

[44] T. E. Larson, D. O. Breecker, Adsorption isotope effects for carbon dioxide from illite- and quartz-packed column experiments. Chem. Geol. 370, 58–68 (2014).

[45] F. P. Fanale, W. A. Cannon, Adsorption on the Martian regolith. Nature 230, 502–504 (1971).

[46] B. M. Jakosky, C. S. Edwards, Inventory of CO_2_ available for terraforming Mars. Nat Astron 2, 634–639 (2018).

[47] P. B. Buhler, S. Piqueux, Obliquity-driven CO_2_ exchange between Mars’ atmosphere, regolith, and polar cap. J. Geophys. Res. Planets 126, e2020JE006759 (2021).

[48] L. Greenspan, Humidity fixed points of binary saturated aqueous solutions. J. Res. Natl. Bur. Stand. A Phys. Chem. 81A, 89–96 (1977).

[49] T. Wang, A. Hamann, D. Spittlehouse, C. Carroll, Locally downscaled and spatially customizable climate data for historical and future periods for North America. PLOS ONE 11, e0156720 (2016). 10.1371/journal.pone.0156720 PMC4898765 27275583

[50] J. V. Mills, Controls on Inorganic Calcite Growth From the Nano to Field Scale, Proquest (2021); www.proquest.com/openview/e1477467f0fa3a7ccf263e0fb5cc1c21/1?pq-origsite=gscholar&cbl=18750&diss=y.

[51] B. F. Cox, J. W. Hillhouse, L. A. Owen, “Pliocene and Pleistocene Evolution of the Mojave River, and Associated Tectonic Development of the Transverse Ranges and Mojave Desert, Based on Borehole Stratigraphy Studies and Mapping of Landforms and Sediments near Victorville, California” in *Paleoenvironments and Paleohydrology of the Mojave and Southern Great Basin Deserts*, Y. Enzel, S. G. Wells, N. Lancaster, Eds. (Geological Society of America, 2003), pp. 1–42.

[52] R. J. Pruett, H. L. Webb, Sampling and analysis of KGa-1B well-crystallized kaolin source clay. Clays Clay Miner. 41, 514–519 (1993).

[53] R. G. Amundson, O. A. Chadwick, J. M. Sowers, H. E. Doner, Soil evolution along an altitudinal transect in the eastern Mojave Desert of Nevada, USA. Geoderma 43, 349–371 (1989).

[54] J. W. Harden, *Soils Developed in Granitic Alluvium near Merced, California* (Government Printing Office, 1987).

[55] T. Saito, H. Fujimaki, H. Yasuda, M. Inoue, Empirical temperature calibration of capacitance probes to measure soil water. Soil Sci. Soc. Am. J. 73, 1931–1937 (2009).

[56] G. R. Blake, K. H. Hartge, “Bulk density” in *Methods of Soil Analysis*, A. Klute, Ed. (John Wiley & Sons, Ltd, 1986), pp. 363–375.

[57] B. H. Sheldrick, C. Wang, “Particle size distribution” in *Soil Sampling and Methods of Analysis*, M. R. Carter, Ed. (CRC Press, 1993), pp. 499–511.

[58] J. Tang, L. Misson, A. Gershenson, W. Cheng, A. H. Goldstein, Continuous measurements of soil respiration with and without roots in a ponderosa pine plantation in the Sierra Nevada Mountains. Agric. For. Meteorol. 132, 212–227 (2005).

[59] R. Vargas, M. F. Allen, Dynamics of fine root, fungal rhizomorphs, and soil respiration in a mixed temperate forest: integrating sensors and observations. Vadose Zone J. 7, 1055–1064 (2008).

[60] P. Moldrup, T. Olesen, T. Yamaguchi, P. Schjønning, D. E. Rolston, Modeling diffusion and reaction in soils: IX. The Buckingham-Burdine-Campbell equation for gas diffusivity in undisturbed soil. Soil Sci. 164, 542–551 (1999).

[61] R. Vargas, M. Detto, D. D. Baldocchi, M. F. Allen, Multiscale analysis of temporal variability of soil CO_2_ production as influenced by weather and vegetation. Glob. Chang. Biol. 16, 1589–1605 (2010).

[62] T. J. Kessler, C. F. Harvey, The global flux of carbon dioxide into groundwater. Geophys. Res. Lett. 28, 279–282 (2001).

[63] L. N. Plummer, E. Busenberg, The solubilities of calcite, aragonite and vaterite in CO_2_-H_2_O solutions between 0 and 90°C, and an evaluation of the aqueous model for the system CaCO_3_-CO_2_-H_2_O. Geochim. Cosmochim. Acta 46, 1011–1040 (1982).

[64] B. A. Ball, R. A. Virginia, J. E. Barrett, A. N. Parsons, D. H. Wall, Interactions between physical and biotic factors influence CO_2_ flux in Antarctic dry valley soils. Soil Biol. Biochem. 41, 1510–1517 (2009).

[65] B. A. Ball, R. A. Virginia, Controls on diel soil CO_2_ flux across moisture gradients in a polar desert. Antarct. Sci. 27, 527–534 (2015).

[66] N. Bekin, N. Agam, Rethinking the deployment of static chambers for CO_2_ flux measurement in dry desert soils. Biogeosciences 20, 3791–3802 (2023).

[67] A.-M. Doucet, F. Jones, K. E. Raymond, G. Dipple, T. A. Black, B. Ladd, K. U. Mayer, Quantitative analysis of diurnal CO_2_ flux variations above an alkaline playa. Vadose Zone J. 22, e20292 (2023).

[68] K. Fa, Y. Zhang, G. Lei, B. Wu, S. Qin, J. Liu, W. Feng, Z. Lai, Underestimation of soil respiration in a desert ecosystem. Catena 162, 23–28 (2018).

[69] X. Li, D. Guo, C. Zhang, D. Niu, H. Fu, C. Wan, Contribution of root respiration to total soil respiration in a semi-arid grassland on the Loess Plateau, China. Sci. Total Environ. 627, 1209–1217 (2018). 10.1016/j.scitotenv.2018.01.313 30857085

[70] J. Liu, W. Feng, Y. Zhang, X. Jia, B. Wu, S. Qin, K. Fa, Z. Lai, Abiotic CO_2_ exchange between soil and atmosphere and its response to temperature. Environ. Earth Sci. 73, 2463–2471 (2015).

[71] J. Ma, R. Liu, Y. Li, Abiotic contribution to total soil CO_2_ flux across a broad range of land-cover types in a desert region. J. Arid. Land 9, 13–26 (2017).

[72] F. L. Shanhun, P. C. Almond, T. J. Clough, C. M. S. Smith, Abiotic processes dominate CO_2_ fluxes in Antarctic soils. Soil Biol. Biochem. 53, 99–111 (2012).

[73] A. Thomazini, M. R. Francelino, A. B. Pereira, A. L. Schünemann, E. S. Mendonça, P. H. A. Almeida, C. E. G. R. Schaefer, Geospatial variability of soil CO_2_–C exchange in the main terrestrial ecosystems of Keller Peninsula, Maritime Antarctica. Sci. Total Environ. 562, 802–811 (2016). 10.1016/j.scitotenv.2016.04.043 27110991

[74] F. Yang, M. Ali, X. Zheng, Q. He, X. Yang, W. Huo, F. Liang, S. Wang, Diurnal dynamics of soil respiration and the influencing factors for three land-cover types in the hinterland of the Taklimakan Desert, China. J. Arid Land 9, 568–579 (2017).

[75] F. Yang, J. Huang, Q. He, X. Zheng, C. Zhou, H. Pan, W. Huo, H. Yu, X. Liu, L. Meng, Impact of differences in soil temperature on the desert carbon sink. Geoderma 379, 114636 (2020).

[76] F. Yang, J. Huang, C. Zhou, X. Yang, A. Mamtimin, X. Zheng, W. Huo, F. Ji, D. Han, L. Meng, J. Gao, M. Song, Y. Wang, C. Zhu, Desert abiotic carbon sequestration weakening by precipitation. Environ. Sci. Technol. 57, 7174–7184 (2023). 10.1021/acs.est.2c09470 37079659

[77] X. Yu, X. Li, L. Xu, M. Wang, J. Zhang, M. Jiang, G. Wang, Diurnal variation of soil CO_2_ efflux and its optimal measuring time-window of temperate meadow steppes in western Songnen Plain, China. Chin. Geogr. Sci. 26, 518–526 (2016).

[78] T. Zhang, G. Wang, Y. Yang, T. Mao, X. Chen, Non-growing season soil CO_2_ flux and its contribution to annual soil CO_2_ emissions in two typical grasslands in the permafrost region of the Qinghai-Tibet Plateau. Eur. J. Soil Biol. 71, 45–52 (2015).

[79] X. Zhou, Y. Zhang, Seasonal pattern of soil respiration and gradual changing effects of nitrogen addition in a soil of the Gurbantunggut Desert, northwestern China. Atmos. Environ. 85, 187–194 (2014).

[80] N. Bekin, D. S. Ashilenje, A. Chehbouni, L. Bouchaou, D. Kool, N. Agam, Pioneering evidence of the dynamics of water vapor and CO_2_ fluxes in Sahara Desert soils. J. Geophys. Res. Biogeosci. 130, e2025JG009193 (2025).

