# Supplementary material for: Thermally driven CO2 adsorption/desorption and its effect on soil respiration
Source: Sci Adv. 2026 Sep 23;12(39):eaed9979. doi: 10.1126/sciadv.aed9979 (PMC13600216; doi:10.1126/sciadv.aed9979)
Supplement: Supplementary file 1 [file sciadv.aed9979_sm.pdf]

Science Advances
AAAS

Supplementary Materials for

Thermally driven CO_2 adsorption/desorption and its effect on soil respiration

Anna Abramova *et al.*

Corresponding author: Anna Abramova, anna_abramova@berkeley.edu; Ronald Amundson, earthy@berkeley.edu

Sci. Adv. **12**, eaed9979 (2026)
DOI: 10.1126/sciadv.aed9979

The PDF file includes:

Figs. S1 to S22
Table S1
Legends for data S1 to S6
References

Other Supplementary Material for this manuscript includes the following:

Data S1 to S6

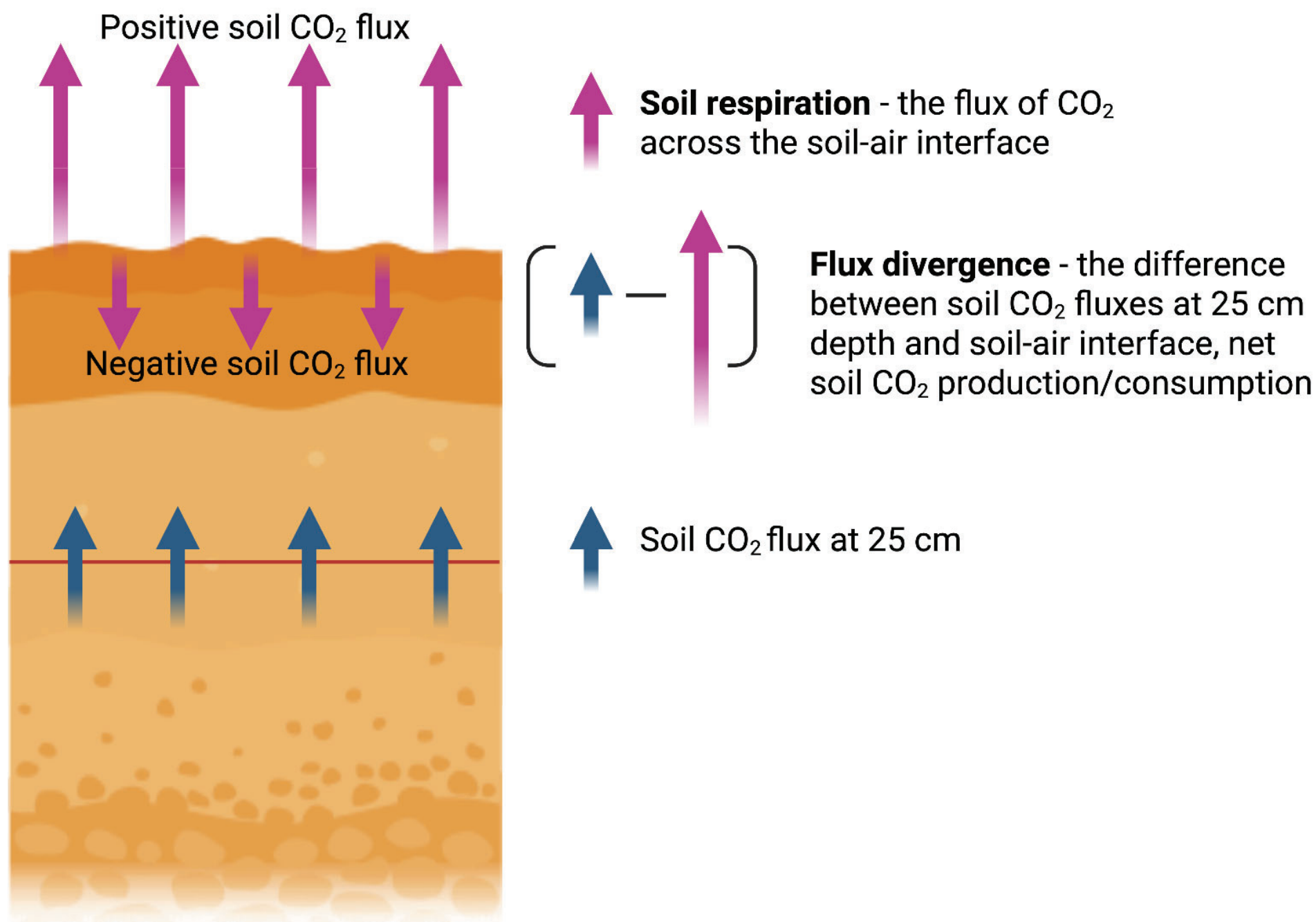

Fig. S1. Schematic representation of soil respiration, soil CO_2 fluxes, and flux divergence. Soil respiration (pink arrows) represents the net flux of CO_2 across the soil-air interface, integrating both positive (upward, efflux) and negative (downward, influx) components. Soil CO_2 flux at 25 cm depth (blue arrows) represents the upward diffusive flux at that depth. Flux divergence is calculated as the difference between the soil CO_2 flux at 25 cm depth and soil respiration at the surface, providing a measure of net CO_2 production or consumption within the upper 25 cm of the soil profile. Created in BioRender. A, A. (2026) https://BioRender.com/t5979jt

A

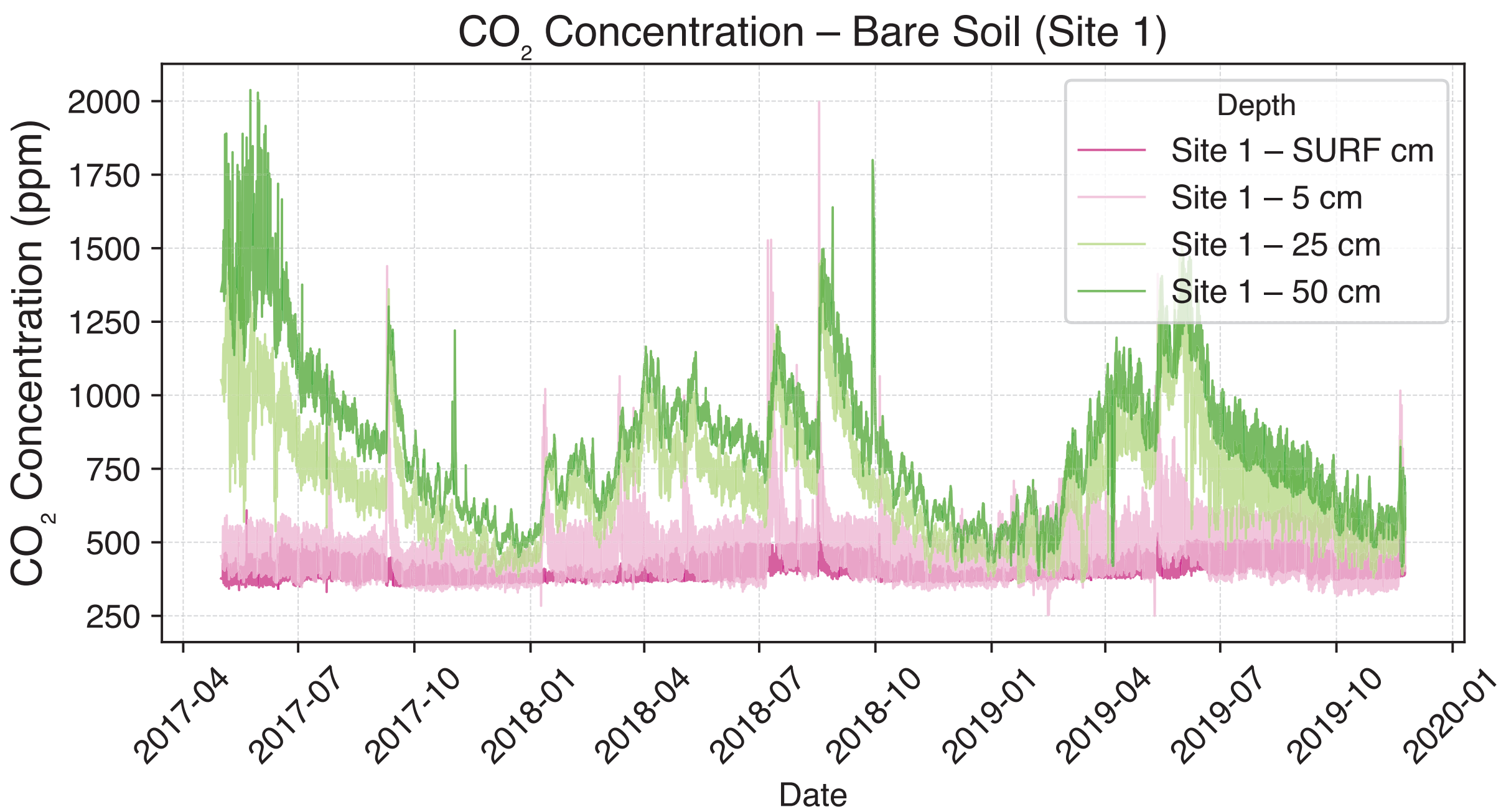

B

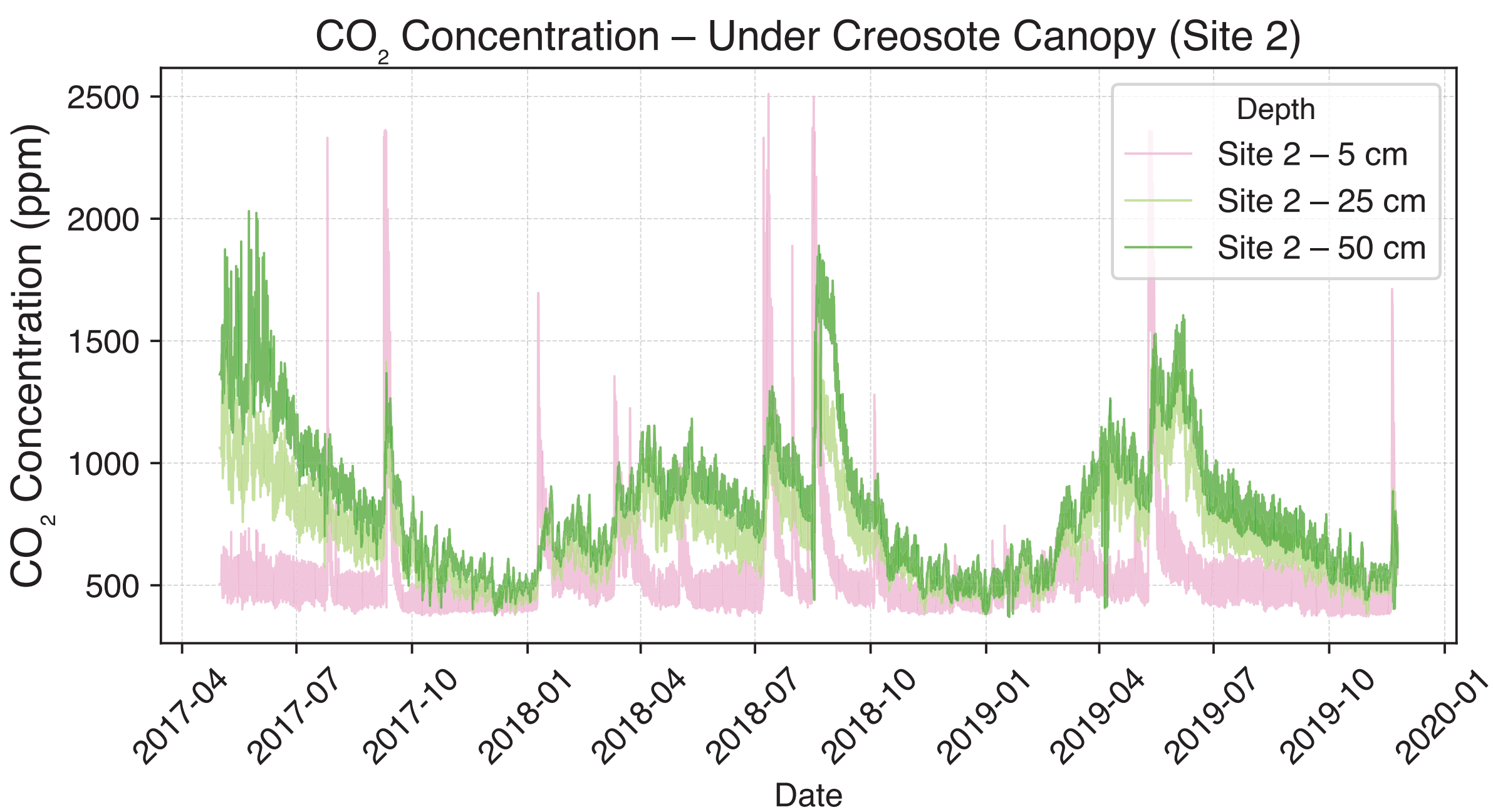

Fig. S2. Time series of soil CO_2 concentrations at various depths in Mojave Desert soils from May 2017 to October 2019. (A) Bare soil (site 1) and **(B)** under creosote canopy (site 2). Over the 2.5-year recording period, soil CO_2 concentrations varied across the soil profile between approximately 250 and 2500 ppm (corresponding to 0.025 - 0.25 kPa). In the shallowest soil layer (0 - 5 cm depth), CO_2 concentrations at the bare soil site dropped as low as 250 ppm (0.025 kPa) on 10 May 2019 at 02:00, while minimum concentrations under the canopy reached 283 ppm (corresponding to 0.028 kPa) on 17 March 2017 at 06:40. Peak shallow-layer concentrations reached approximately 2000 ppm (corresponding to 0.2 kPa) on 17 August 2018

at 10:30 at the bare soil site and approximately 2500 ppm (corresponding to 0.25 kPa) on 11 July 2018 at 18:40 under the canopy. The highest concentration recorded across all depths and sites occurred under the creosote canopy at 50 cm depth, reaching approximately 3000 ppm (corresponding to 0.3 kPa) on 16 March 2017 at 10:30. At the bare soil site, CO_2 concentrations were measured at four depths (surface, 5, 25, and 50 cm), whereas under the canopy, concentrations were measured at three depths (5, 25, and 50 cm).

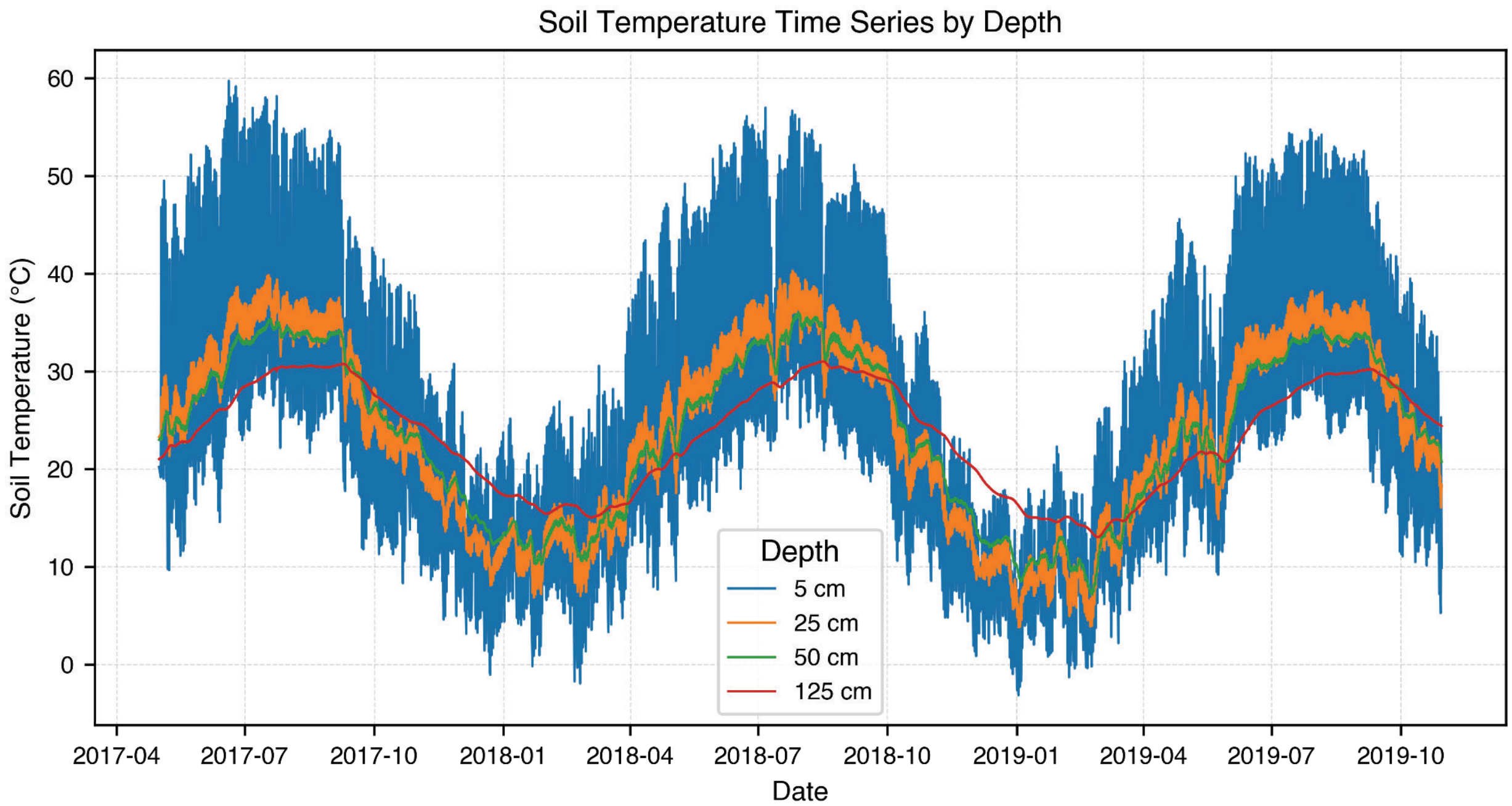

Fig. S3. Time series of soil temperatures at various depths in Mojave Desert soils from May 2017 to October 2019. Shallow soils (5 cm) showed large diurnal temperature fluctuations, ranging from about 30-60°C during summer to 0-20°C in winter. Temperature variation decreased substantially with depth; at 25 cm (orange) and 50 cm (green), diurnal ranges were noticeably reduced.

A

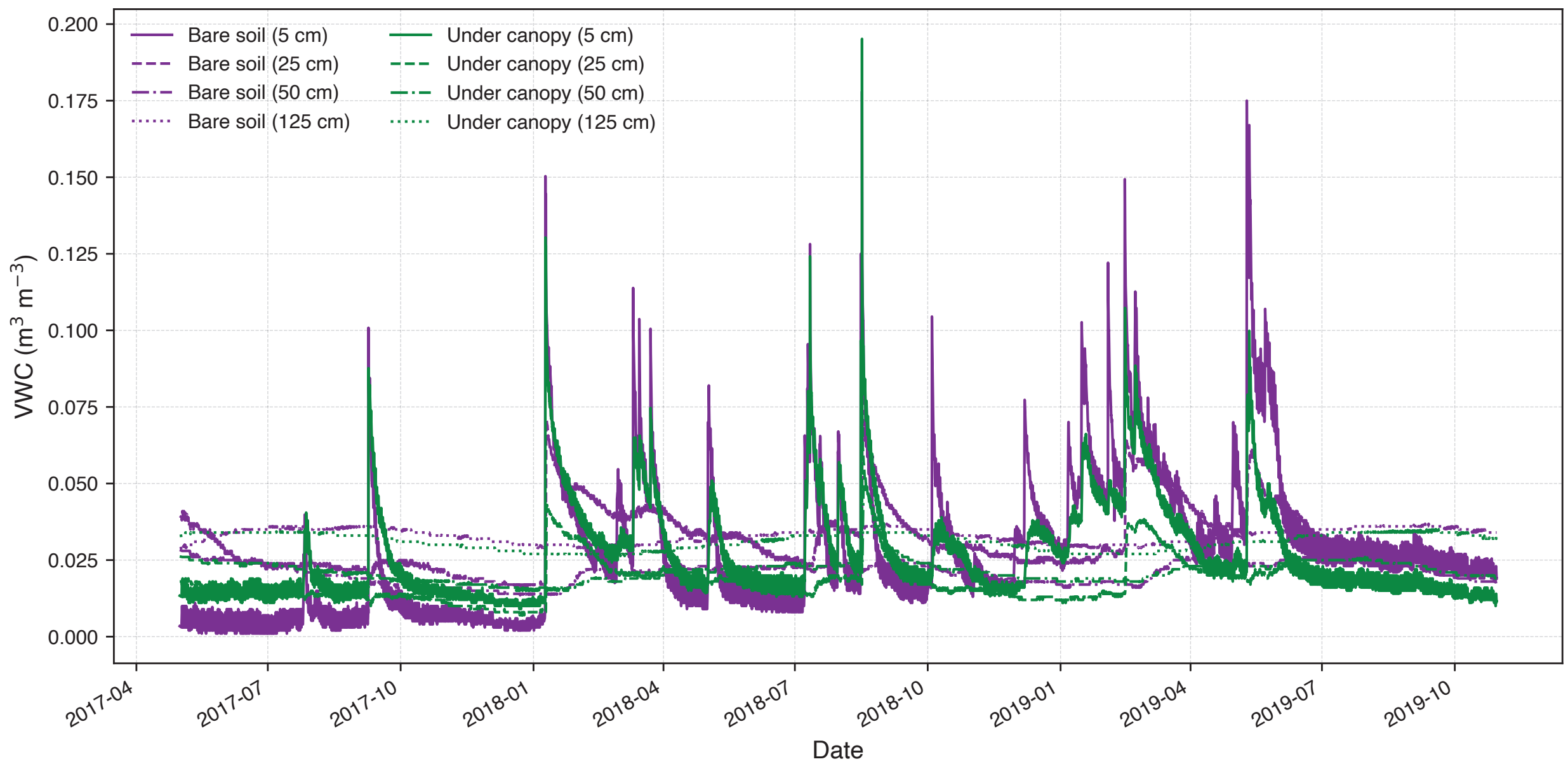

B

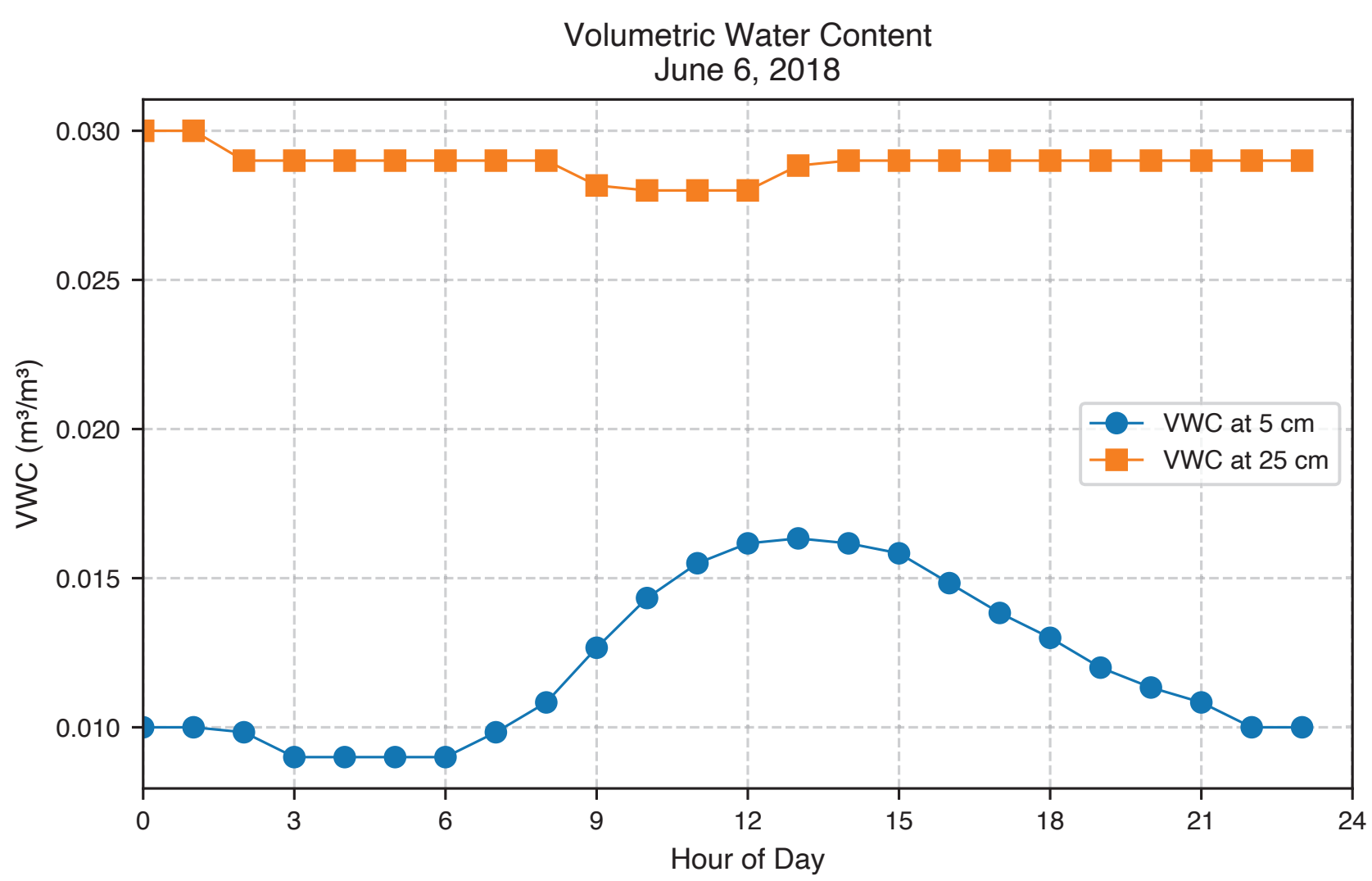

Fig. S4. Volumetric water content (VWC) at various depths in Mojave Desert soils: (A) from May 2017 to October 2019, (B) diurnal cycle on June 6, 2018. Both sites showed low baseline moisture levels, generally remaining below 0.025 (m^3 m^{-3}) at 5 cm depth and 0.03 (m^3 m^{-3}) at 25 cm depth. Precipitation events produced sharp, episodic spikes in VWC. During observed negative nighttime soil CO_2 fluxes (**Fig. S5**), the soil's volumetric water content was very low, not exceeding 0.025-0.03 ($cm^3 cm^{-3}$) at 5 cm depth and 0.03 ($cm^3 cm^{-3}$) at 25 cm depth. In contrast to dry months, prominent CO_2 pulses from the soil to the atmosphere were observed periodically after rain events, particularly following peaks in volumetric water content (VWC) at 5 cm soil depth, which reached 0.15-0.2 ($cm^3 cm^{-3}$).

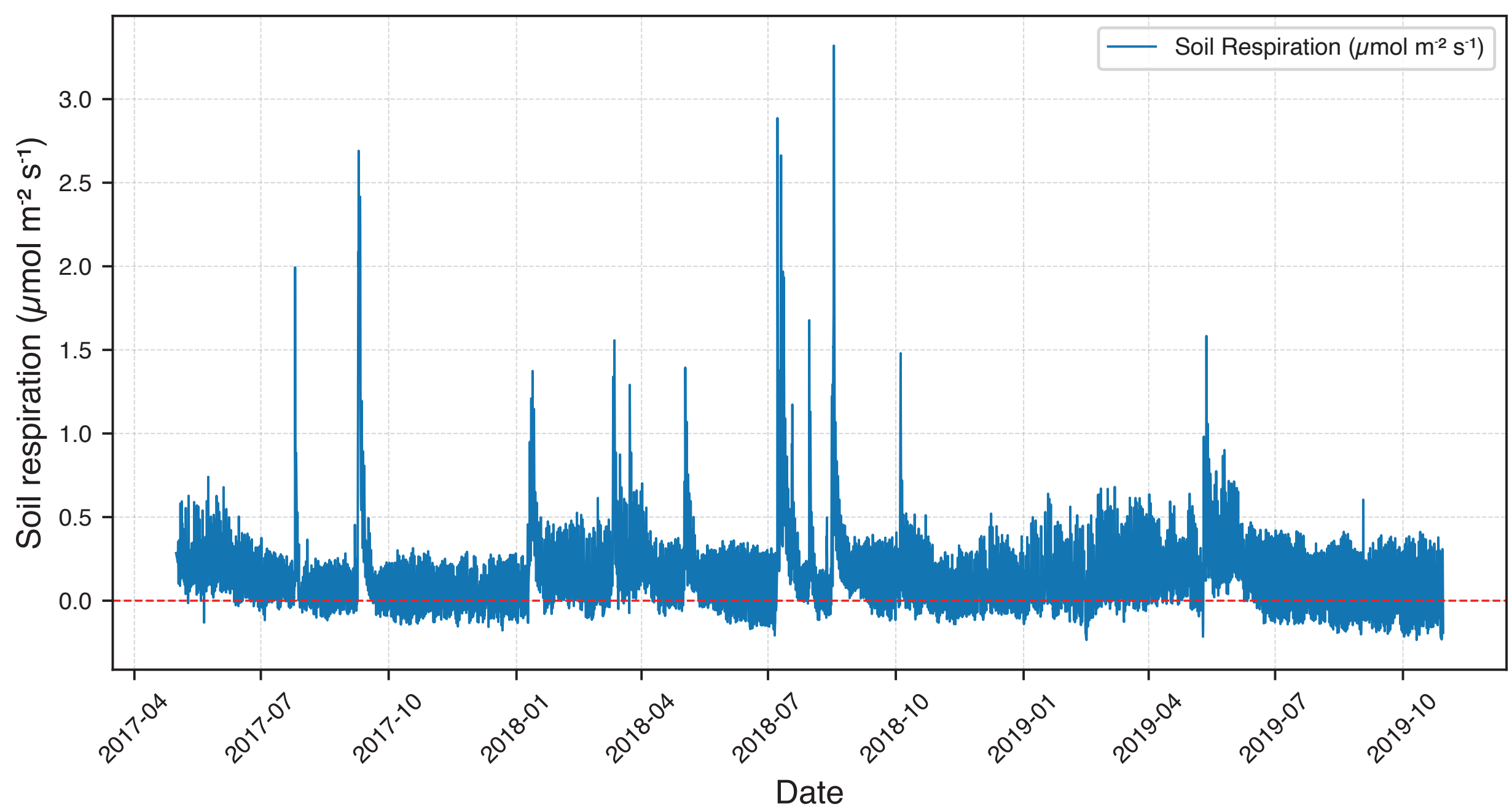

Fig. S5. Time series of calculated soil respiration in Mojave Desert soils from May 2017 to October 2019. Soil respiration (μmol m^{-2} s^{-1}) was calculated as the CO_2 flux across the soil-air interface (Methods). In the Mojave Desert site, consistent occurrences of negative soil respiration were observed across all months, with monthly average negative soil respiration flux values ranging from -0.01 to -0.12 μmol m^{-2} s^{-1}. The most pronounced negative flux was recorded in June 2018 (-0.09 ± 0.04 μmol m^{-2} s^{-1}), August 2019 (-0.1 ± 0.04 μmol m^{-2} s^{-1}), September 2019 (-0.1 ± 0.05 μmol m^{-2} s^{-1}), and October 2019 (-0.12 ± 0.05 μmol m^{-2} s^{-1}). The highest magnitudes of negative fluxes generally occurred during the summer months (June through August), particularly in 2019, when values reached as low as -0.10 μmol m^{-2} s^{-1} or lower. Winter months (January, February, and December) typically showed smaller negative values, around -0.05 ± 0.03 μmol m^{-2} s^{-1}.

A.

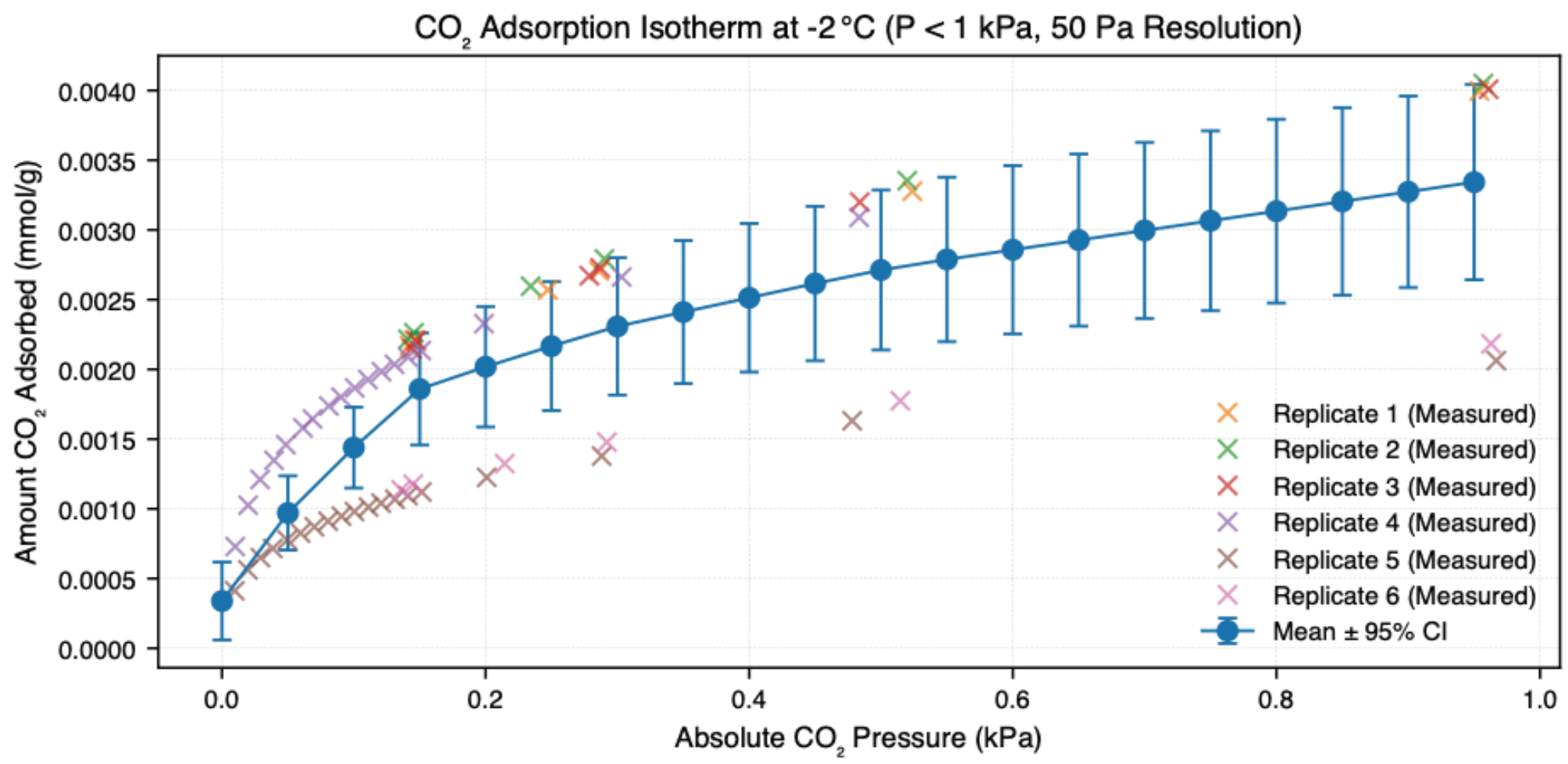

CO_2 Adsorption Isotherm at -2 °C (P < 1 kPa, 50 Pa Resolution)
Amount CO_2 Adsorbed (mmol/g)
Absolute CO_2 Pressure (kPa)
0.0000
0.0005
0.0010
0.0015
0.0020
0.0025
0.0030
0.0035
0.0040
0.0
0.2
0.4
0.6
0.8
1.0
Replicate 1 (Measured)
Replicate 2 (Measured)
Replicate 3 (Measured)
Replicate 4 (Measured)
Replicate 5 (Measured)
Replicate 6 (Measured)
Mean ± 95% CI

B.

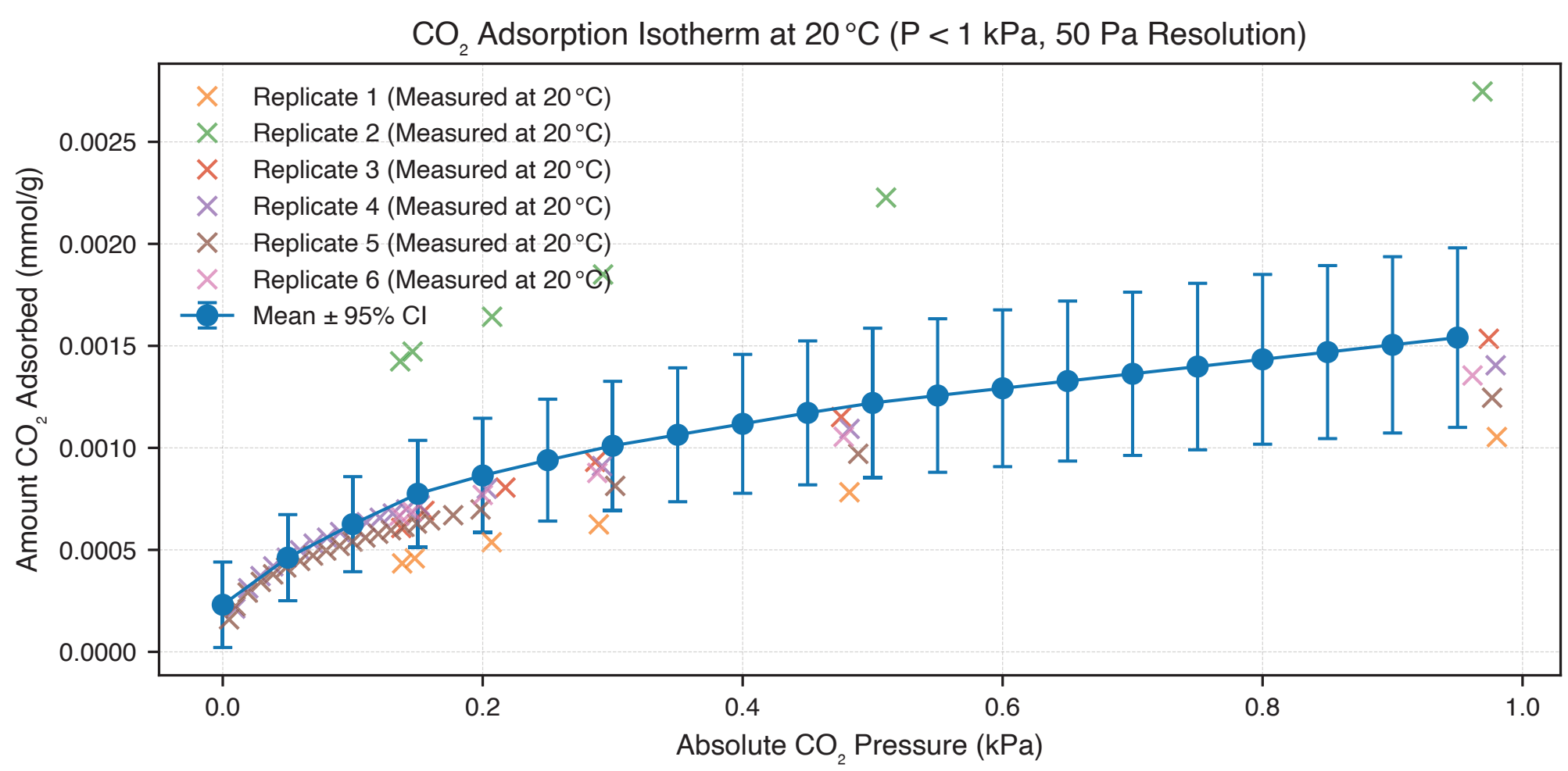

CO_2 Adsorption Isotherm at 20 °C (P < 1 kPa, 50 Pa Resolution)
Amount CO_2 Adsorbed (mmol/g)
Absolute CO_2 Pressure (kPa)
0.0000
0.0005
0.0010
0.0015
0.0020
0.0025
0.0
0.2
0.4
0.6
0.8
1.0
Replicate 1 (Measured at 20 °C)
Replicate 2 (Measured at 20 °C)
Replicate 3 (Measured at 20 °C)
Replicate 4 (Measured at 20 °C)
Replicate 5 (Measured at 20 °C)
Replicate 6 (Measured at 20 °C)
Mean ± 95% CI

C.

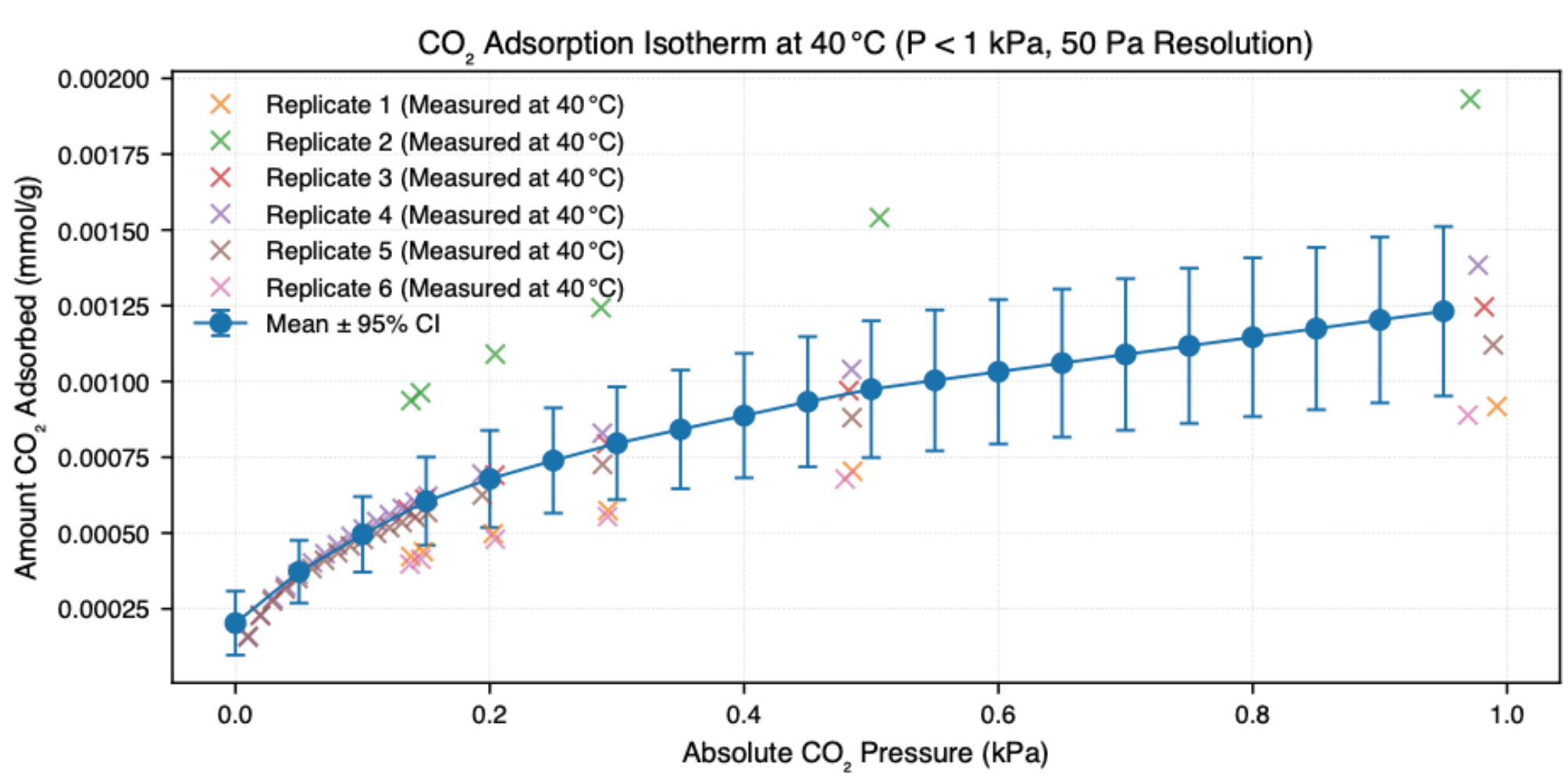

CO_2 Adsorption Isotherm at 40 °C (P < 1 kPa, 50 Pa Resolution)
Amount CO_2 Adsorbed (mmol/g)
Absolute CO_2 Pressure (kPa)
0.00025
0.00050
0.00075
0.00100
0.00125
0.00150
0.00175
0.00200
0.0
0.2
0.4
0.6
0.8
1.0
Replicate 1 (Measured at 40 °C)
Replicate 2 (Measured at 40 °C)
Replicate 3 (Measured at 40 °C)
Replicate 4 (Measured at 40 °C)
Replicate 5 (Measured at 40 °C)
Replicate 6 (Measured at 40 °C)
Mean ± 95% CI

D.

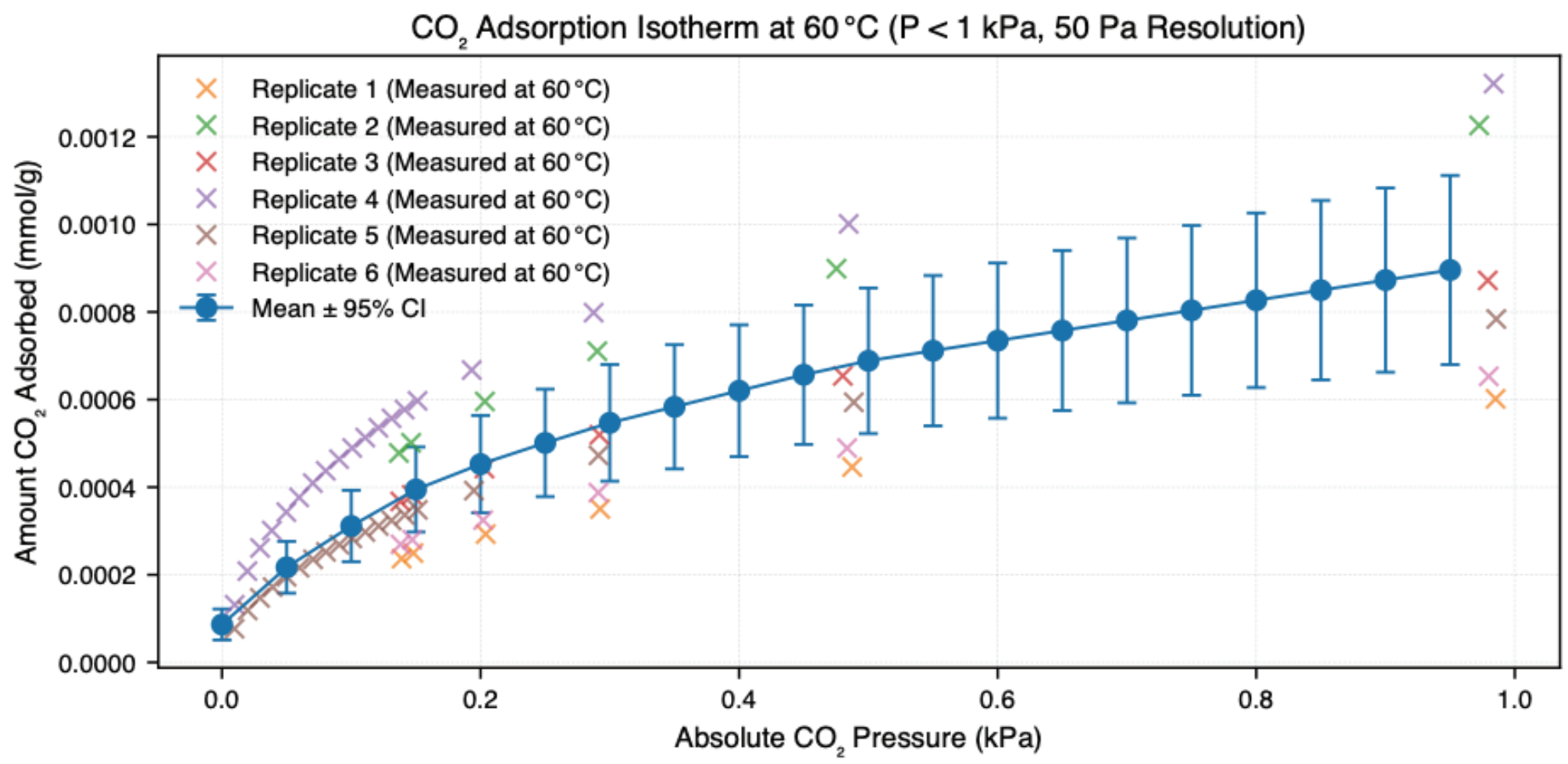

Fig. S6. CO_2 adsorption isotherms measured in triplicate at four different temperatures. (**A**) at -2 °C, (**B**) at 20°C, (**C**) at 40 °C, and (**D**) at 60 °C. Each temperature included six replicates (two sets of triplicates). Calculated and interpolated isotherm values, along with error bars, are shown in blue; individual replicate measurements are represented by colored points.

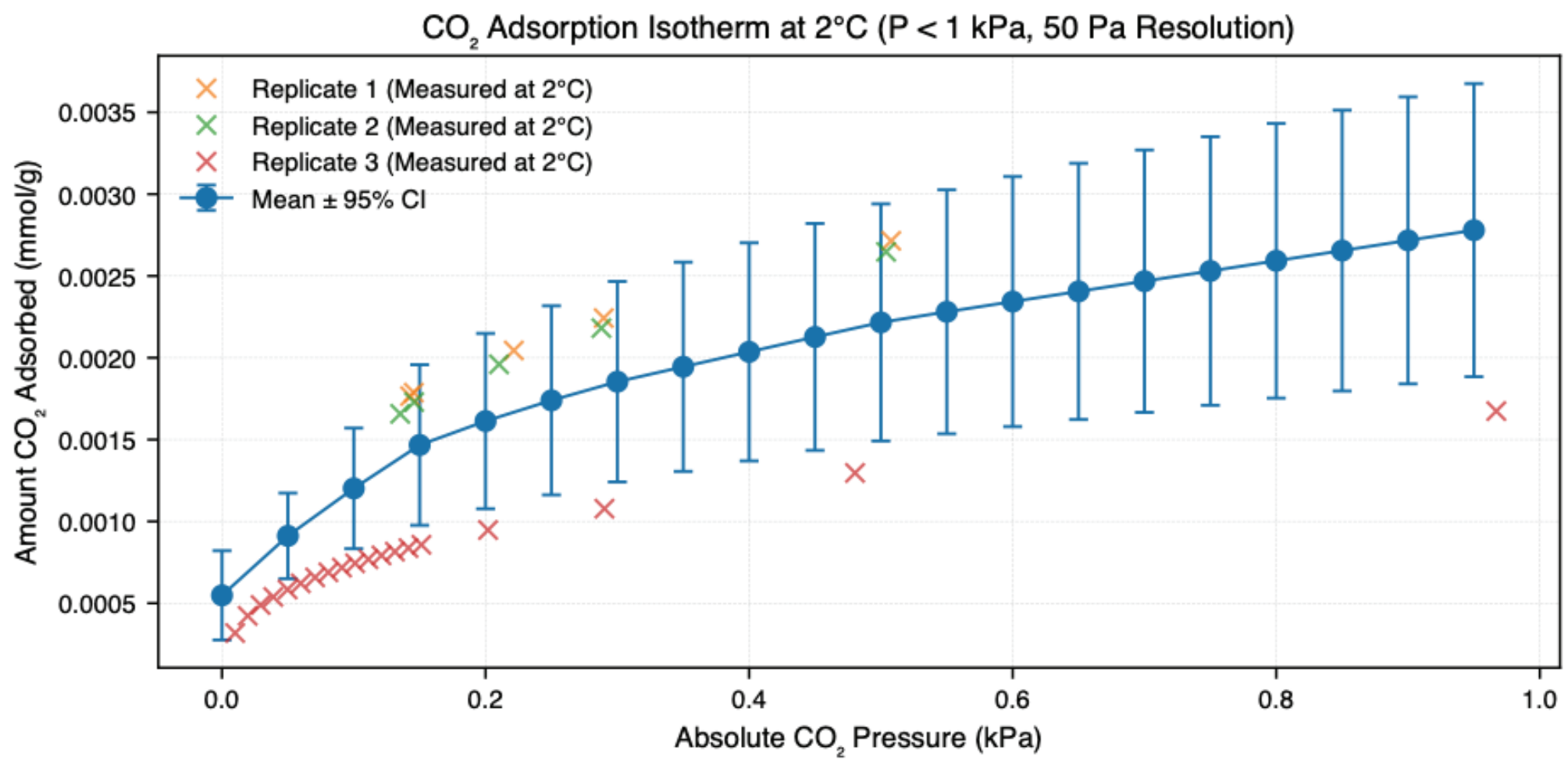

CO2 Adsorption Isotherm at 2°C (P < 1 kPa, 50 Pa Resolution)
Replicate 1 (Measured at 2°C)
Replicate 2 (Measured at 2°C)
Replicate 3 (Measured at 2°C)
Mean ± 95% CI
Amount CO2 Adsorbed (mmol/g)
Absolute CO2 Pressure (kPa)

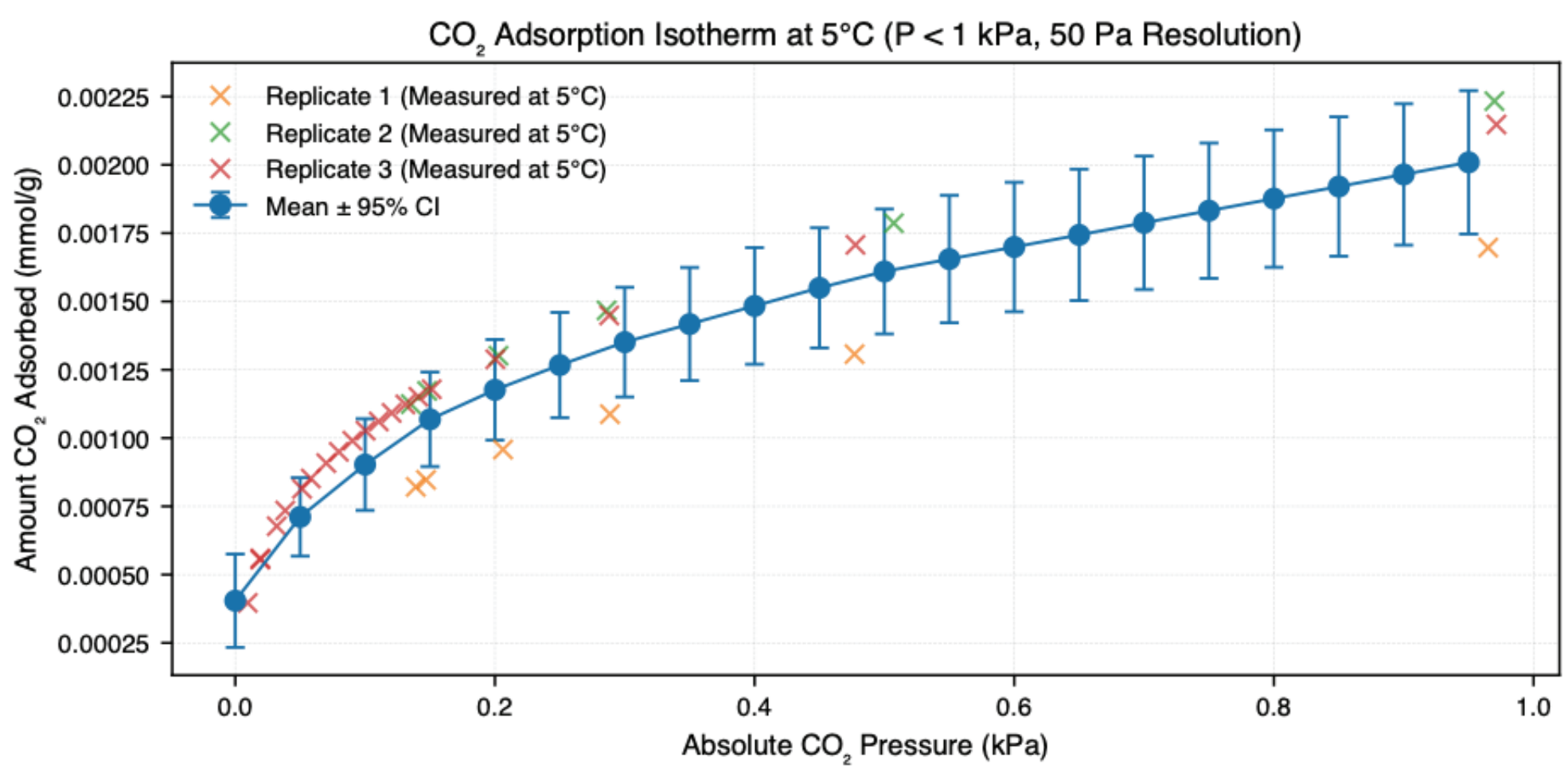

CO2 Adsorption Isotherm at 5°C (P < 1 kPa, 50 Pa Resolution)
Replicate 1 (Measured at 5°C)
Replicate 2 (Measured at 5°C)
Replicate 3 (Measured at 5°C)
Mean ± 95% CI
Amount CO2 Adsorbed (mmol/g)
Absolute CO2 Pressure (kPa)

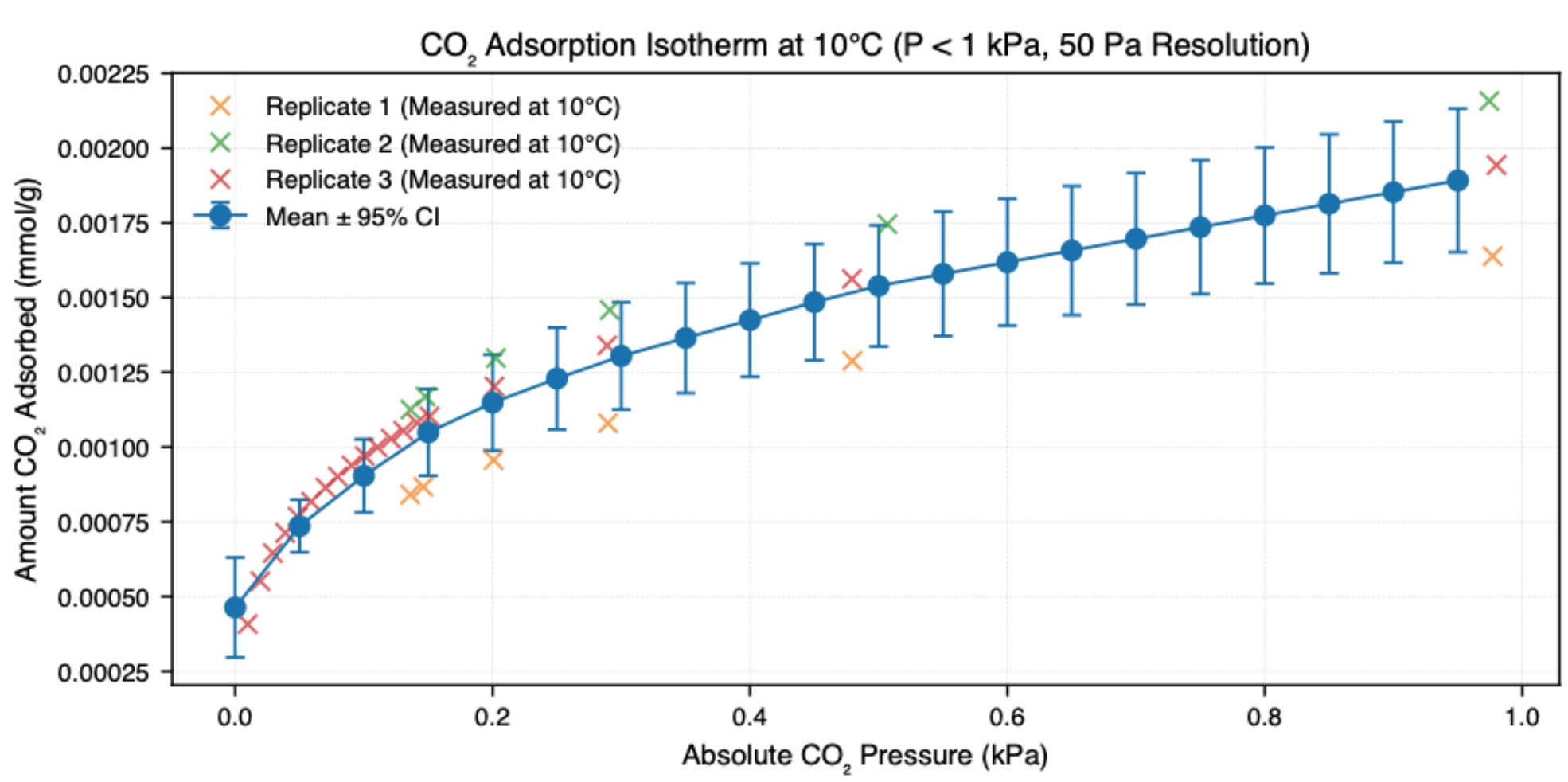

CO2 Adsorption Isotherm at 10°C (P < 1 kPa, 50 Pa Resolution)
Replicate 1 (Measured at 10°C)
Replicate 2 (Measured at 10°C)
Replicate 3 (Measured at 10°C)
Mean ± 95% CI
Amount CO2 Adsorbed (mmol/g)
Absolute CO2 Pressure (kPa)

D.

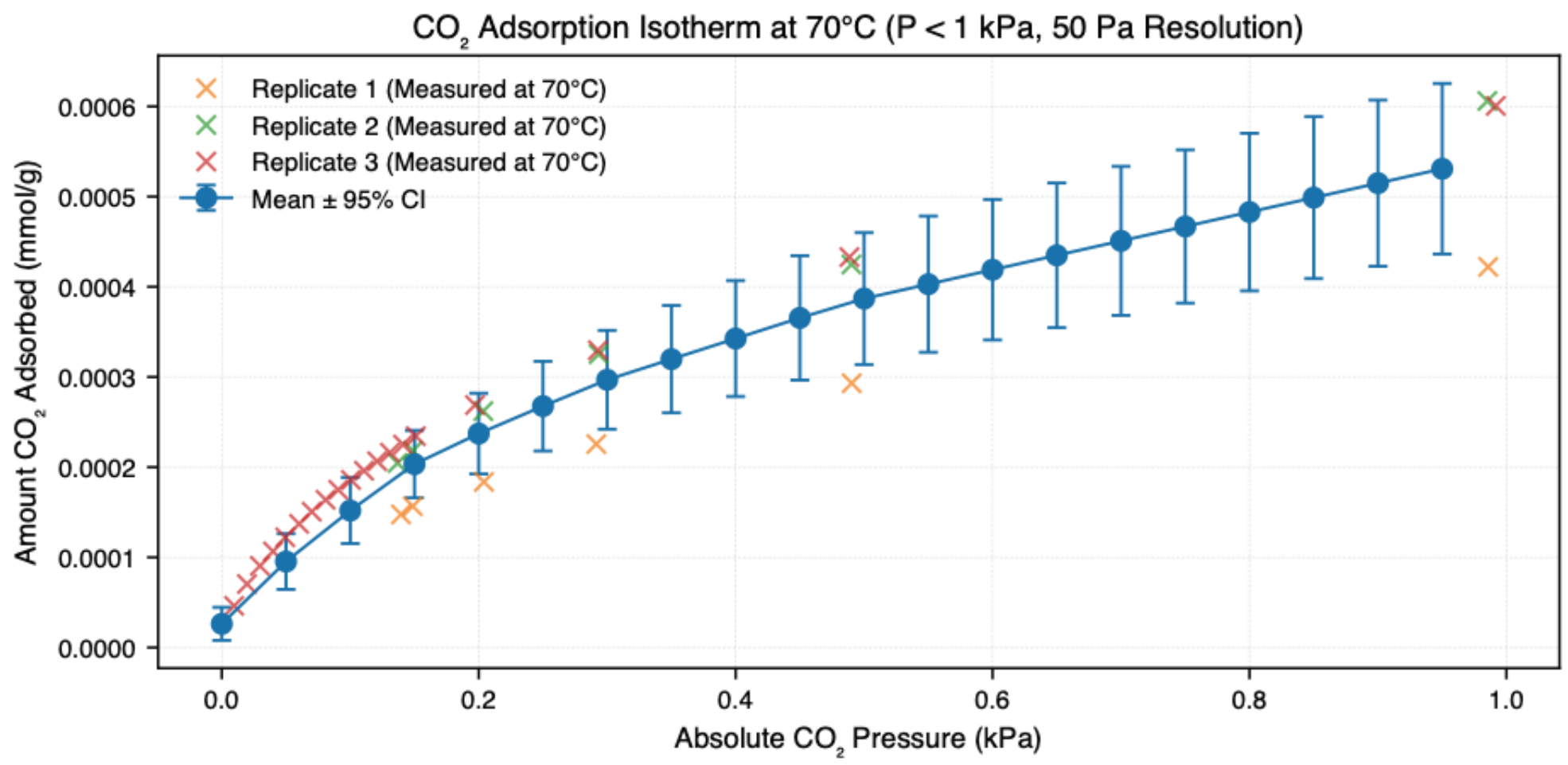

Fig. S7. CO2 adsorption isotherms measured in triplicate at four different temperatures. **(A)** at 2 °C, **(B)** at 5 °C, **(C)** at 10 °C, and **(D)** at 70 °C. Each temperature included three replicates above 0.1 kPa and one replicate below 0.1 kPa; the calculated and interpolated isotherm values and error bars are shown in blue; individual replicate measurements are represented by colored points.

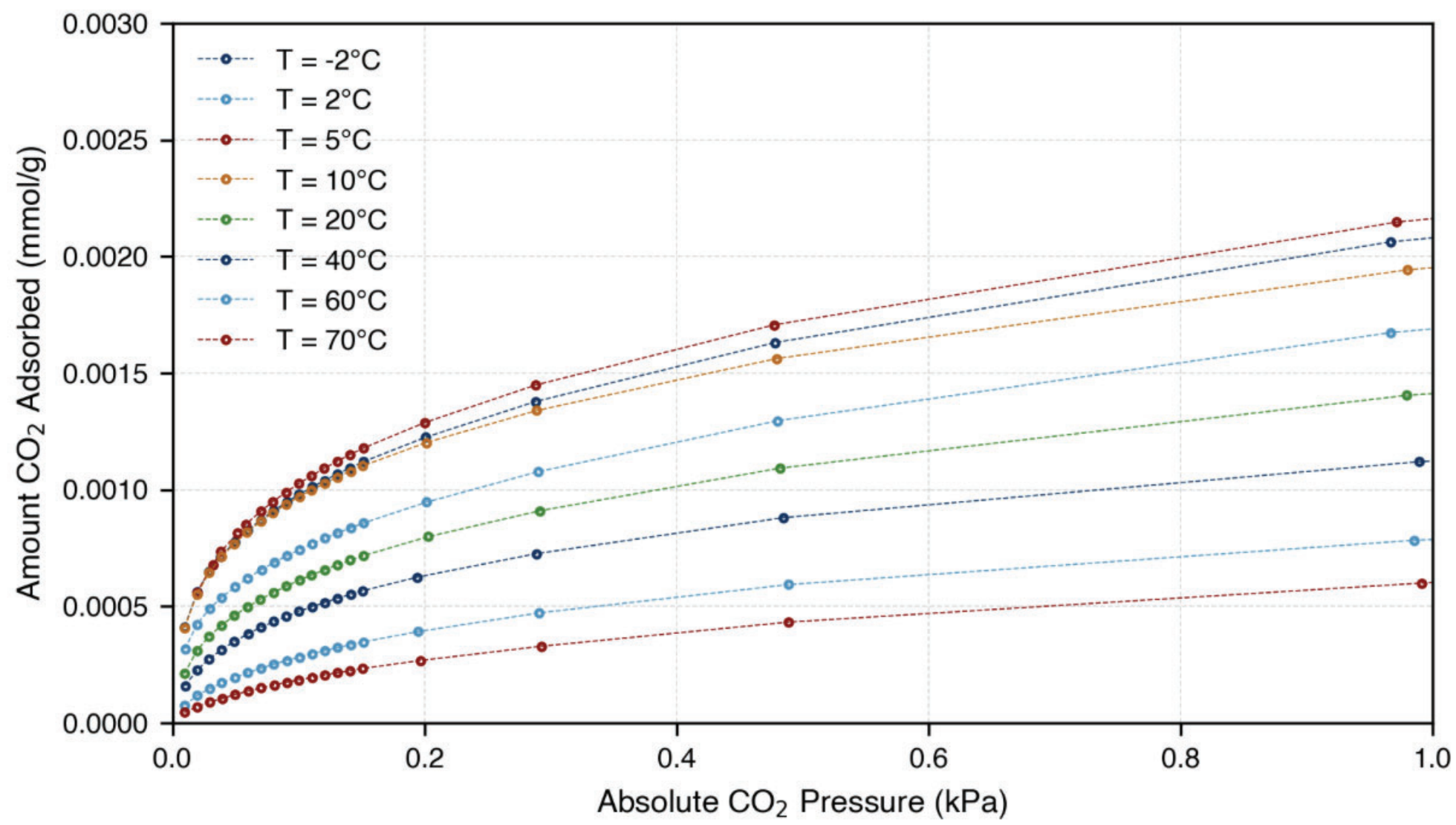

Fig. S8. CO_2 adsorption isotherms for the Mojave Desert soil at temperatures ranging from -2°C to 70°C. All measured isotherms, including those at 5 °C and 10 °C, exhibited high adsorption at low pressures.

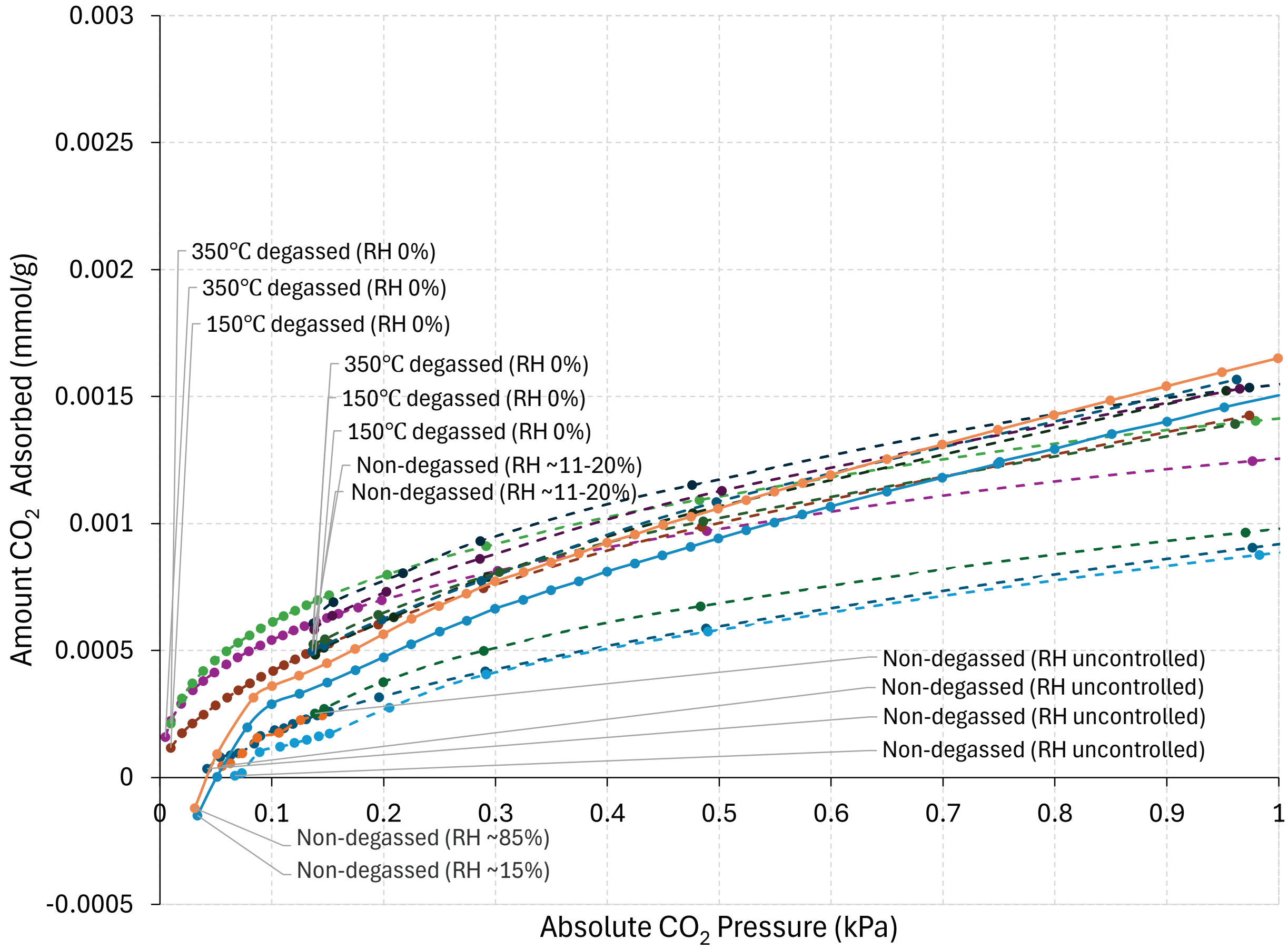

Fig. S9. CO_2 adsorption isotherms at 20°C for soils subjected to different surface preparation conditions: (1) fully or partially degassed and (2) non-degassed (natural conditions). Laboratory conditions correspond to the maximum CO_2 adsorption capacity, whereas field-representative conditions correspond to a fraction of that maximum, estimated from adsorption measurements under various degassing conditions. To examine the natural occupancy of adsorption sites in soils, we conducted a series of adsorption isotherm measurements on samples subjected to different degassing treatments. Specifically, we conducted CO_2 adsorption isotherms at 20°C on soils that were (1) non-degassed (neither heated nor purged) and stored at controlled relative humidity (~15% and 86%), and (2) non-degassed and stored under uncontrolled ambient laboratory conditions, representing conditions in which some adsorption sites are occupied by gases, including water vapor. We compared these results to those for fully degassed (heated and purged with inert gases) soils, which represent the maximum adsorption capacity.

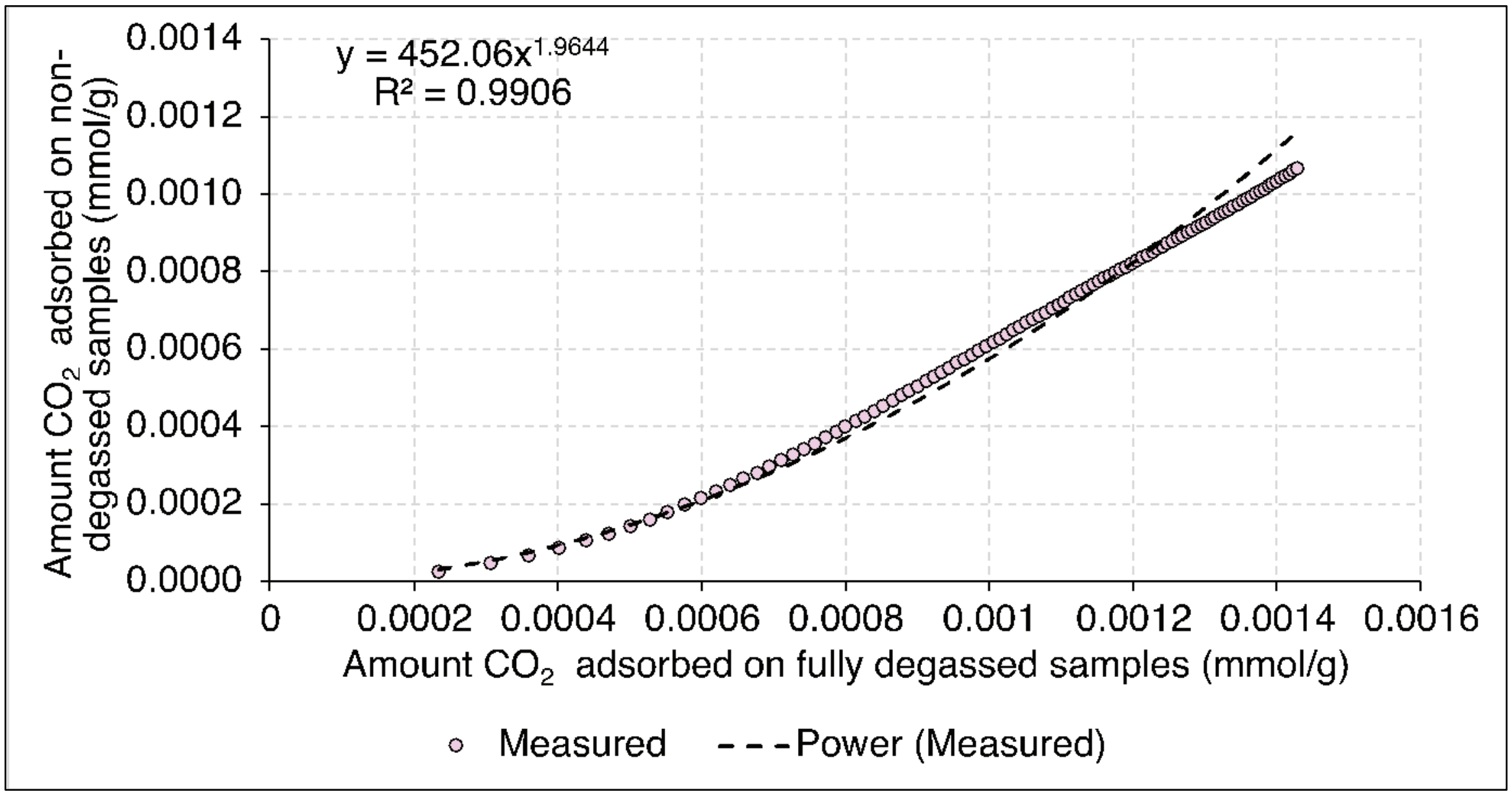

Fig. S10. Laboratory parameter (calibration) conversion. A non-linear empirical relationship was derived between the fully degassed (*Q_lab*, the maximum CO_2 adsorption capacity) and non-degassed conditions with the natural occupancy of adsorption sites in soils (*Q_nat*, a fraction of that maximum): (*Q_nat = 452.06 × Q_lab^1.9644*) This relationship reflects the cumulative effects of water vapor and other gases present in the system, which influence CO_2 adsorption by occupying surface sites and correspond to natural occupancy of adsorption sites in soils. The relationship between degassed and non-degassed conditions varies with the pressure of CO_2 and can be described by a power law model. At the low edge of CO_2 partial pressures (<0.05 kPa CO_2), the reduction in adsorption due to water and other gases is substantial; thus, natural soils may adsorb about 15 % of the CO_2 that soils prepared under ideal experimental conditions can adsorb. As the partial pressure of CO_2 rises, this difference between treatments becomes smaller, and at high CO_2 partial pressures (>0.5 kPa CO_2), natural soils may adsorb about 65-70% (reaching 85-90% at very high CO_2 partial pressures) of the CO_2 that soils prepared under ideal experimental conditions can adsorb.

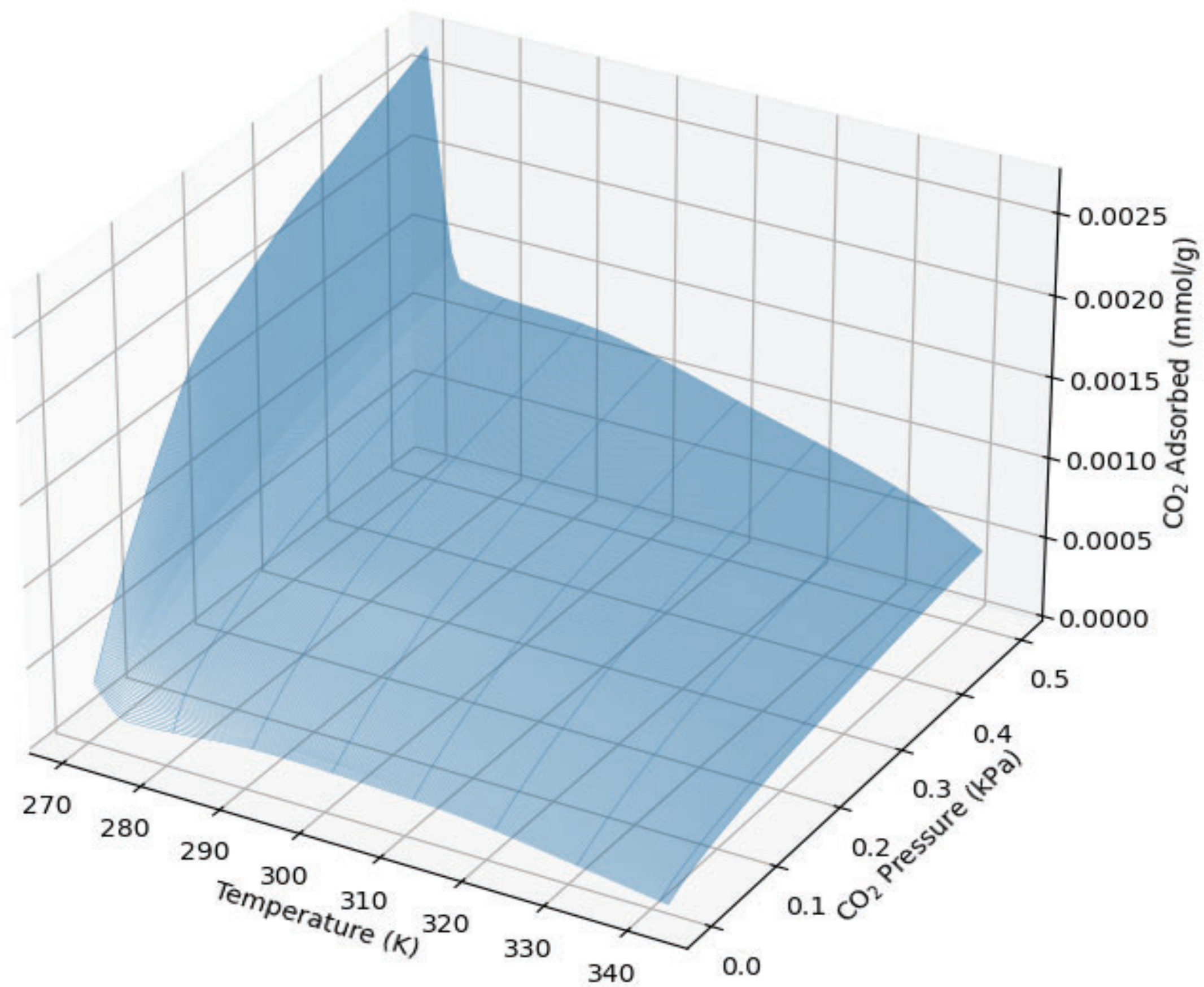

Fig. S11. **Coupling soil CO_2 fluxes with CO_2 adsorption**: **3D surface matrix of temperature, CO_2 partial pressure, and experimentally obtained amounts of CO_2 adsorbed.** Based on the CO_2 adsorption isotherms, we developed a 3D surface matrix that links the experimentally determined amounts of adsorbed CO_2 with the associated temperature and CO_2 partial pressure pair conditions. This matrix was generated using a multiple linear regression method (MLR) with 1°C and 1 ppm steps for temperature and partial pressure parameters. For the calculation of the CO_2 adsorption *Q (T, P)* matrix, we used the isotherms for six temperatures (**Fig. 3**), which showed the following trends: (1) minimum adsorption occurs under conditions of low pressure and high temperature, and (2) maximum adsorption is observed at low temperatures and high pressures.

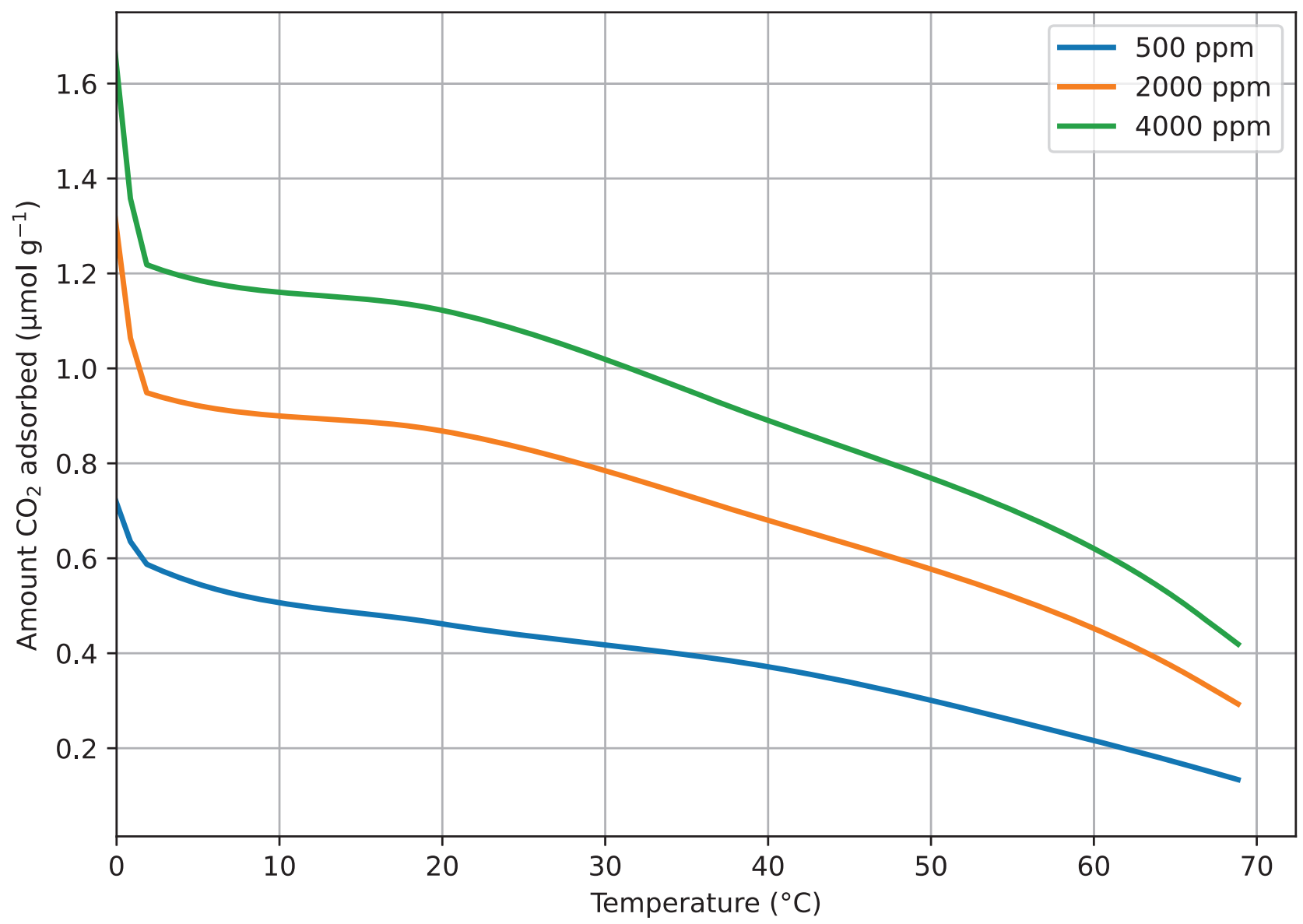

Fig. S12. Temperature dependence of CO_2 adsorption capacity onto the Mojave soils for three CO_2 concentrations (500, 2000, and 4000 ppm). The amount of CO_2 adsorbed μmol g^{-1}) decreases with increasing temperature, while higher CO_2 concentrations result in greater adsorption. Based on the matrix, we extracted CO_2 adsorption capacities as a function of temperature at three different pCO_2 (ppm) (**Fig. S11**).

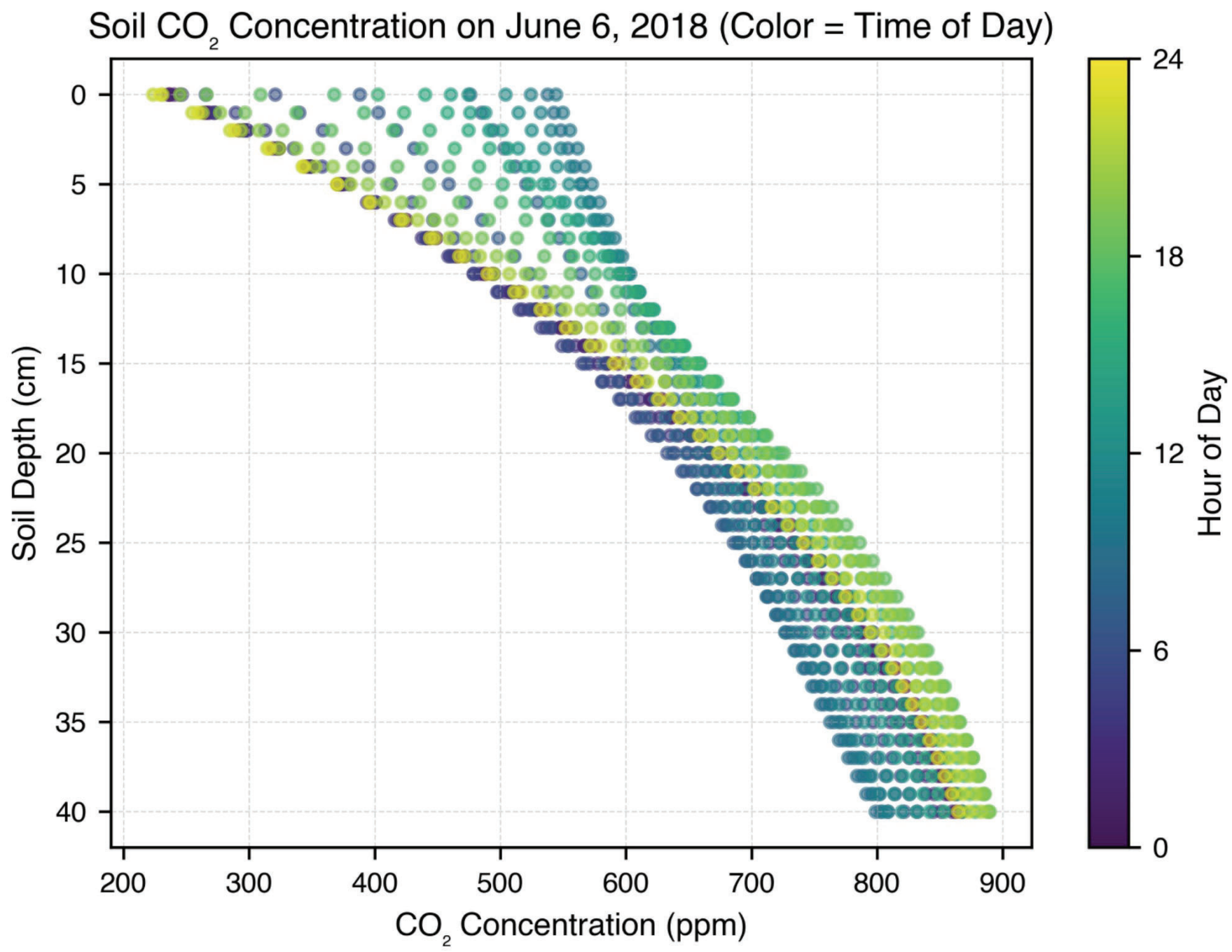

Fig. S13. Soil CO_2 concentration profile on June 6, 2018, colored by time of day. CO_2 concentration increased with soil depth; shallow layers exhibited strong diurnal variability, with CO_2 concentrations fluctuating between ~250 and 600 ppm. In contrast, the deep soil layers (>25 cm) maintained stable and elevated CO_2 levels with minimal variation throughout the day. CO_2 concentrations were recorded at four depths (5 cm, 25 cm, 50 cm, and 125 cm) and the soil surface, and then interpolated at 1 cm intervals throughout the soil profile. Similar to soil temperature, daily fluctuations in CO_2 concentrations decreased with depth, becoming nearly stable below 15-20 cm.

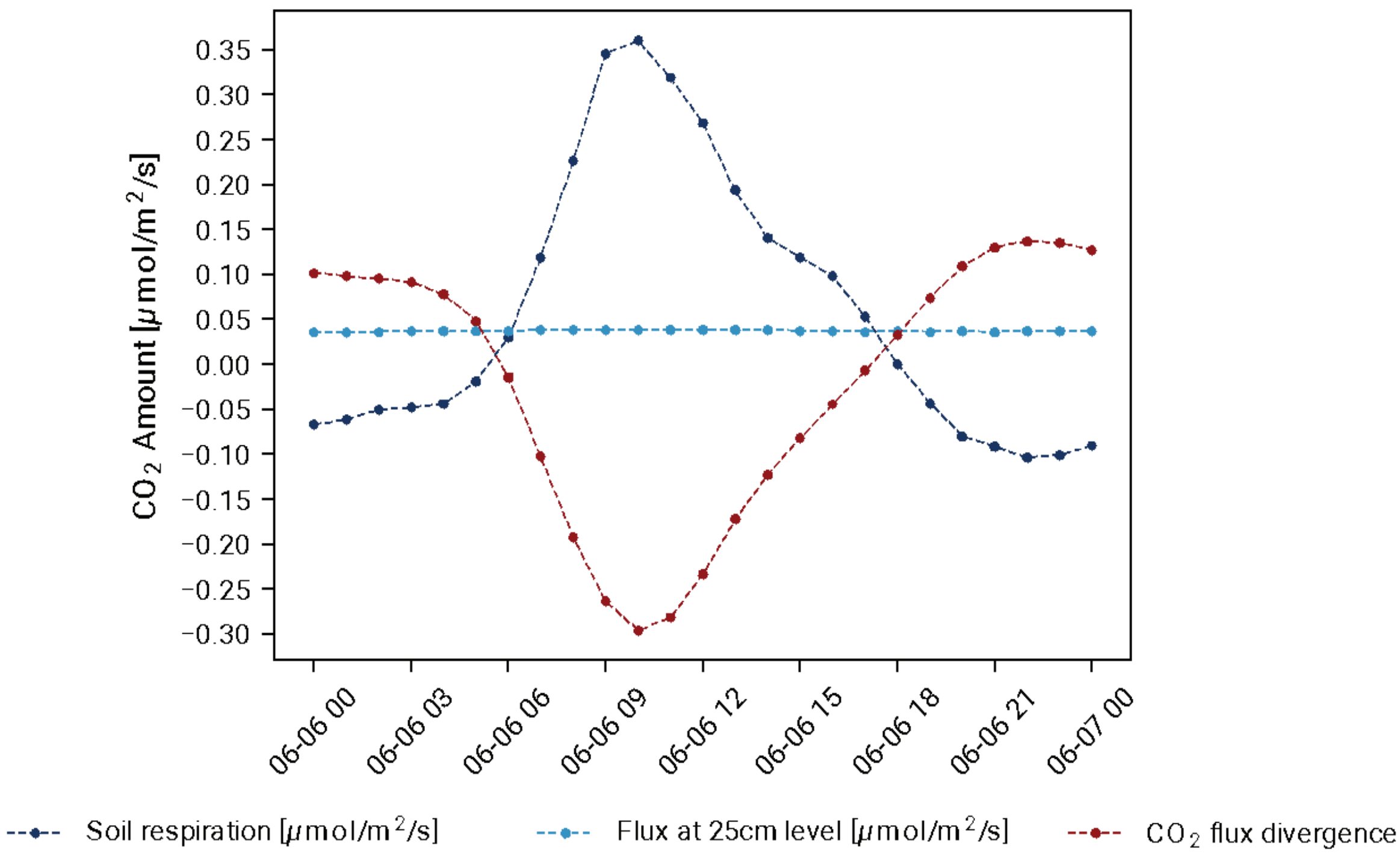

Fig. S14. Diurnal cycle of soil respiration (dark blue), flux at 25 cm level (light blue), and CO_2 flux divergence (red) on June 6, 2018. Unlike soil respiration (CO_2 flux at the soil-air interface), the CO_2 flux below the thermally active layer remained stable throughout day and night. On June 6, 2018, at a depth of 25 cm of the thermally active layer, the soil CO_2 flux was 200 µmol m^{-2} h^{-1} (0.055 µmol $m^{-2}s^{-1}$) and remained steady, indicating diffusive CO_2 transport from deeper soil layers. The flux divergence, the flux at 25 cm depth minus flux at the soil surface, delta $F = F(in) – F(out)$, was positive (~400 µmol m^{-2} h^{-1} (0.1 µmol $m^{-2}s^{-1}$) at night, demonstrating accumulation of CO_2 in the thermally active layer during the nighttime. During the day, the flux divergence had a negative value that reached approximately -1000 µmol m^{-2} h^{-1} (0.28 µmol $m^{-2}s^{-1}$) by 9 AM, indicating the release of CO_2 from the thermally active layer and additional CO_2 production, which then gradually declined throughout the day.

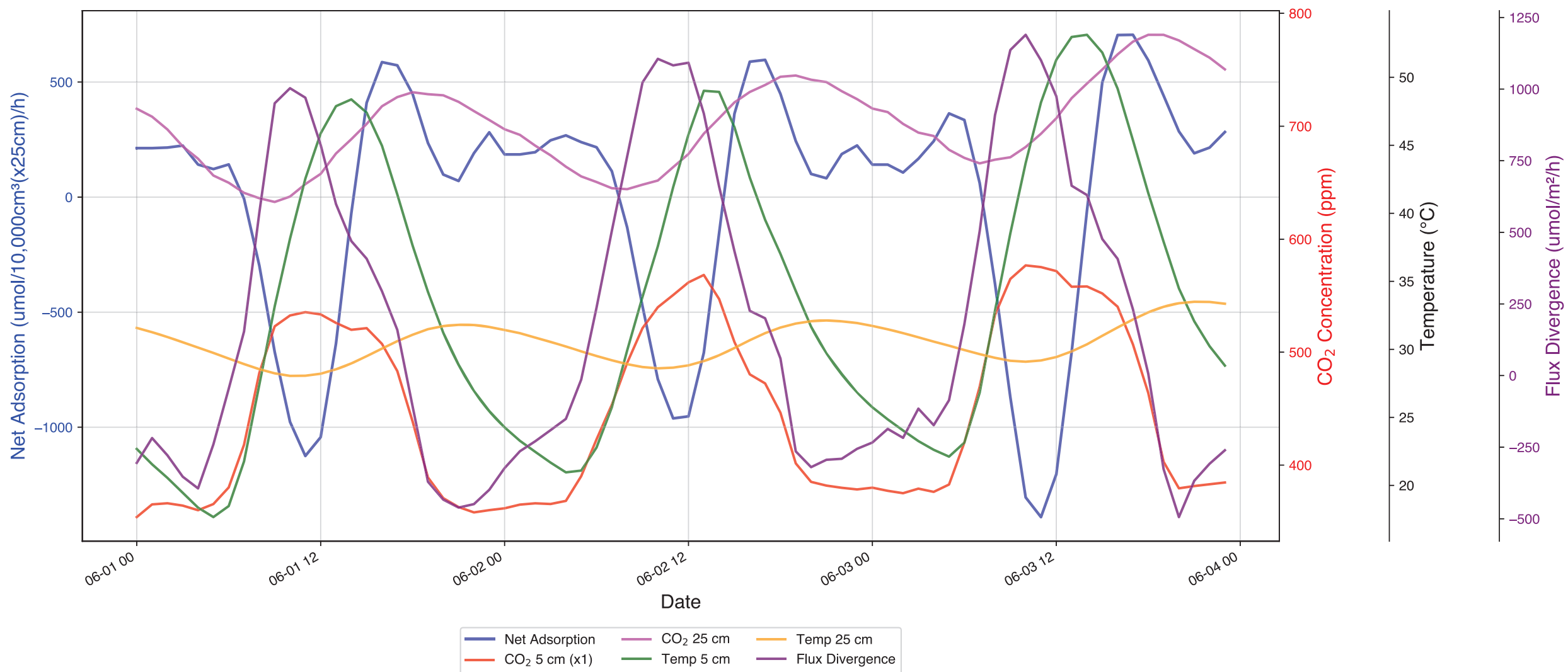

Fig. S15. Diurnal cycles of net adsorption, CO_2 concentrations at 5cm and 25 cm soil depths, soil temperature at 5cm and 25 cm soil depths, and flux divergence. The diurnal cycle shows a clear phase shift between air and soil temperatures at various depths, as well as between CO_2 concentrations at different depths.

A.

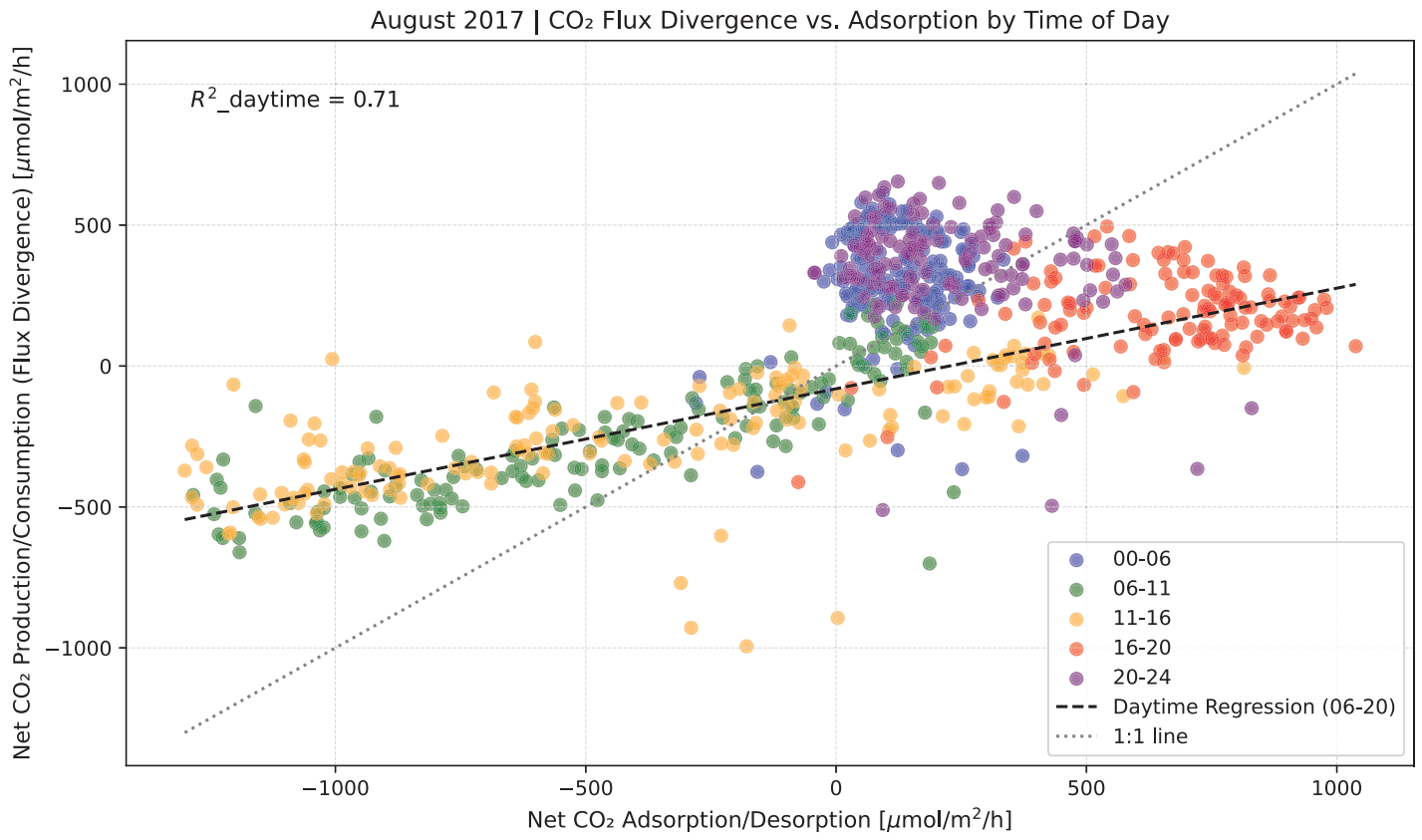

B.

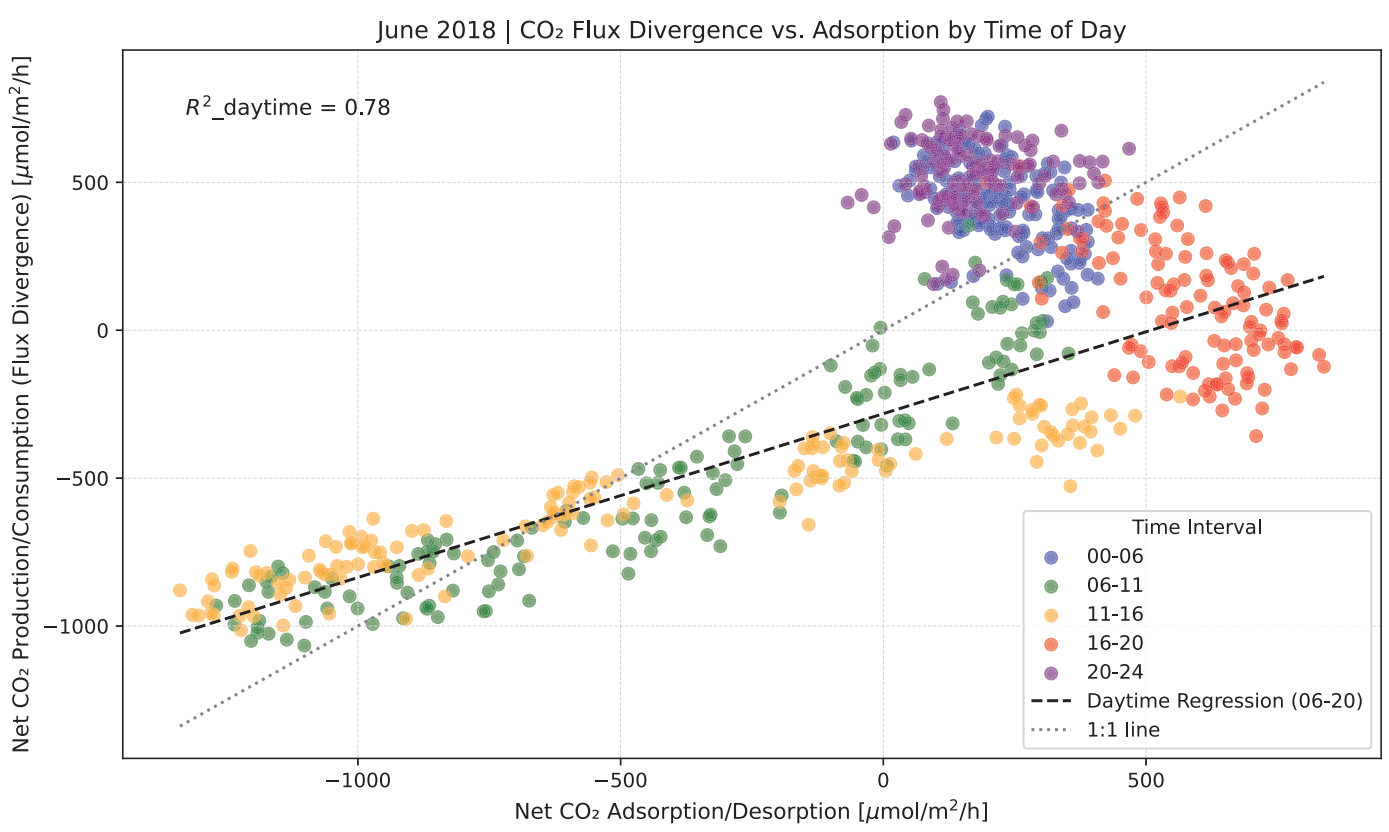

C.

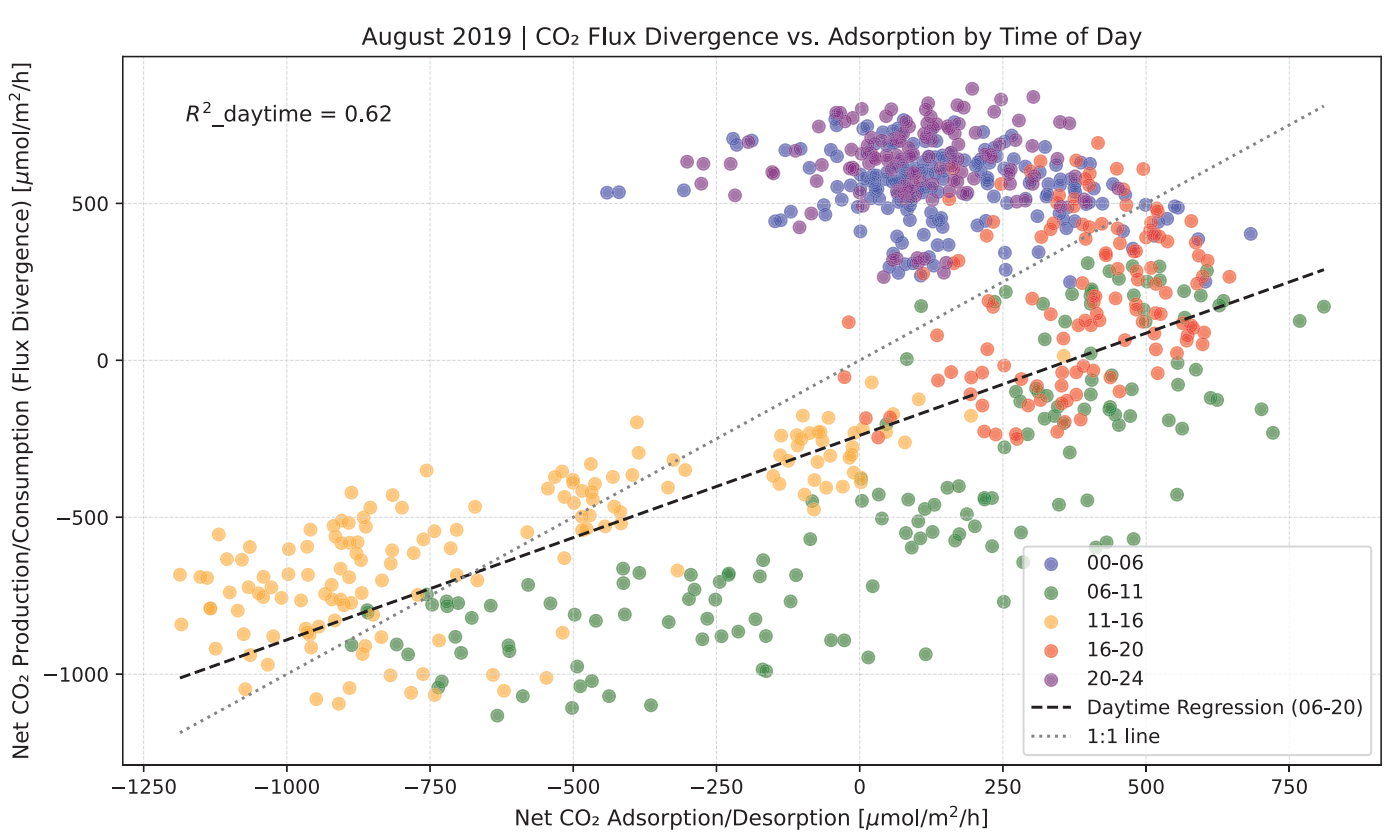

Fig. S16. The relationship between net CO_2 adsorption and desorption, along with net CO_2 production and consumption over a diurnal cycle, for the 0 - 25 cm soil depth interval in **(A)** August, 2017; **(B)** June 2018; and **(C)** August, 2019, with data points colored by time of day (blue: 0 - 6 h, green: 6 - 11h, yellow: 11-16 h, red: 16-20 h, purple: 20 - 24 h).

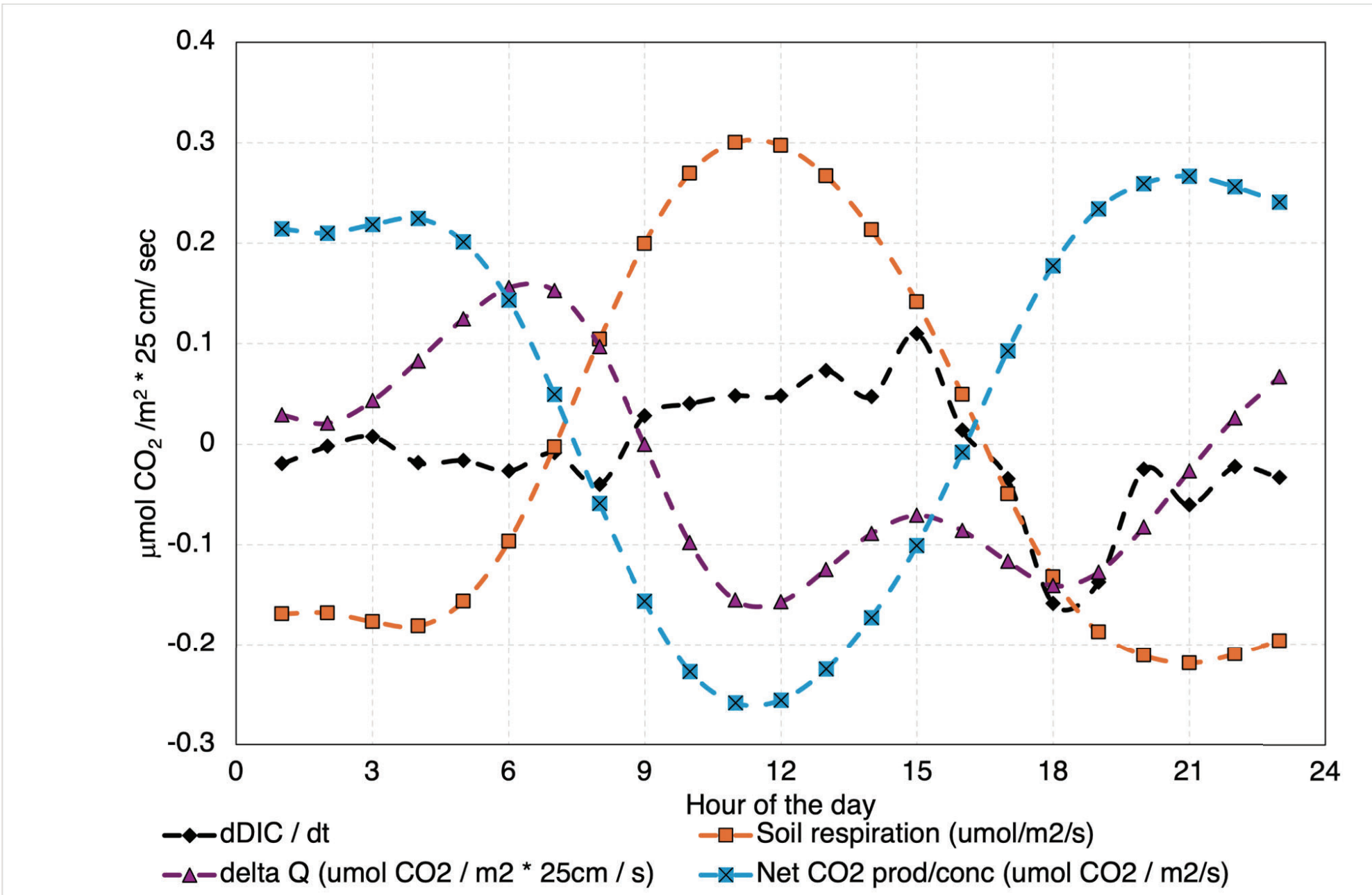

Fig. S17. Diurnal cycle of CO_2 dissolved in soil moisture. Diurnal cycle of inorganic carbon (black) in the studied Mojave Desert soil on October 1, 2019, with the comparison to net CO_2 fluxes in the soil atmosphere (blue), soil respiration (orange), and net adsorption in the thermally active layer (purple). The rate of change in dissolved inorganic carbon (*dDIC/dt*) remains near zero throughout the night, indicating minimal contribution of carbonate and CO_2 dissolution to soil respiration. To interrogate the potential contribution of CO_2 consumption driven by CO_2 uptake into soil water due to temperature-dependent carbonic acid system speciation and potential calcite dissolution, we estimated the amount of CO_2 dissolved in the soil over a 24-hour diurnal cycle on October 1, 2019 - a date when pronounced nighttime CO_2 consumption (-0.16 μmol/m^2/s) was recorded. On that day, volumetric water content remained low ($\theta \leq 3\%$) throughout the 0 - 25 cm depth. Soil temperature at 5 cm depth ranged from 11°C at night to 35°C during the day, while temperature at 25 cm depth remained relatively stable between 23 - 25°C. Measured soil pH in this profile increased with depth, from 7.5 at 5 cm to 8.6 at 25 cm. The soils have relatively young landscape ages and, consequently, exhibited only modest calcite accumulation (0.39-1.14 wt%) at depths of 53-55 cm and below. No carbonates were detected in the upper soil horizons, where negative CO_2 fluxes at night were observed at night. Under these conditions, we estimated that CO_2 dissolved in soil moisture at 5 cm soil depth ranged from approximately 40 (μmol CO_2/m^2 × 1 cm) during the night to 60 (μmol CO_2/m^2 × 1 cm) during the day. At 25 cm depth, the total amount of inorganic carbon was higher (due to higher CO_2 concentrations), about 600 (μmol CO_2/m^2 × 1 cm) during the night and 700 (μmol CO_2/m^2 × 1 cm) during the day. We then interpolated these values to calculate the hourly change in dissolved inorganic carbon (ΔDIC) across the 25 cm soil depth. A positive ΔDIC indicates an increase in dissolved CO_2 in soil water, while a negative ΔDIC suggests net CO_2 release into the soil atmosphere. During the diurnal cycle, ΔDIC (black line) reached up to 0.03 μmol CO_2/m^2/s in the thermally active soil layer (0.25 m^3) at night - approximately an order of magnitude lower than the observed negative CO_2 flux at the surface (orange line), which is around 0.1 (μmol CO_2/m^2/s).

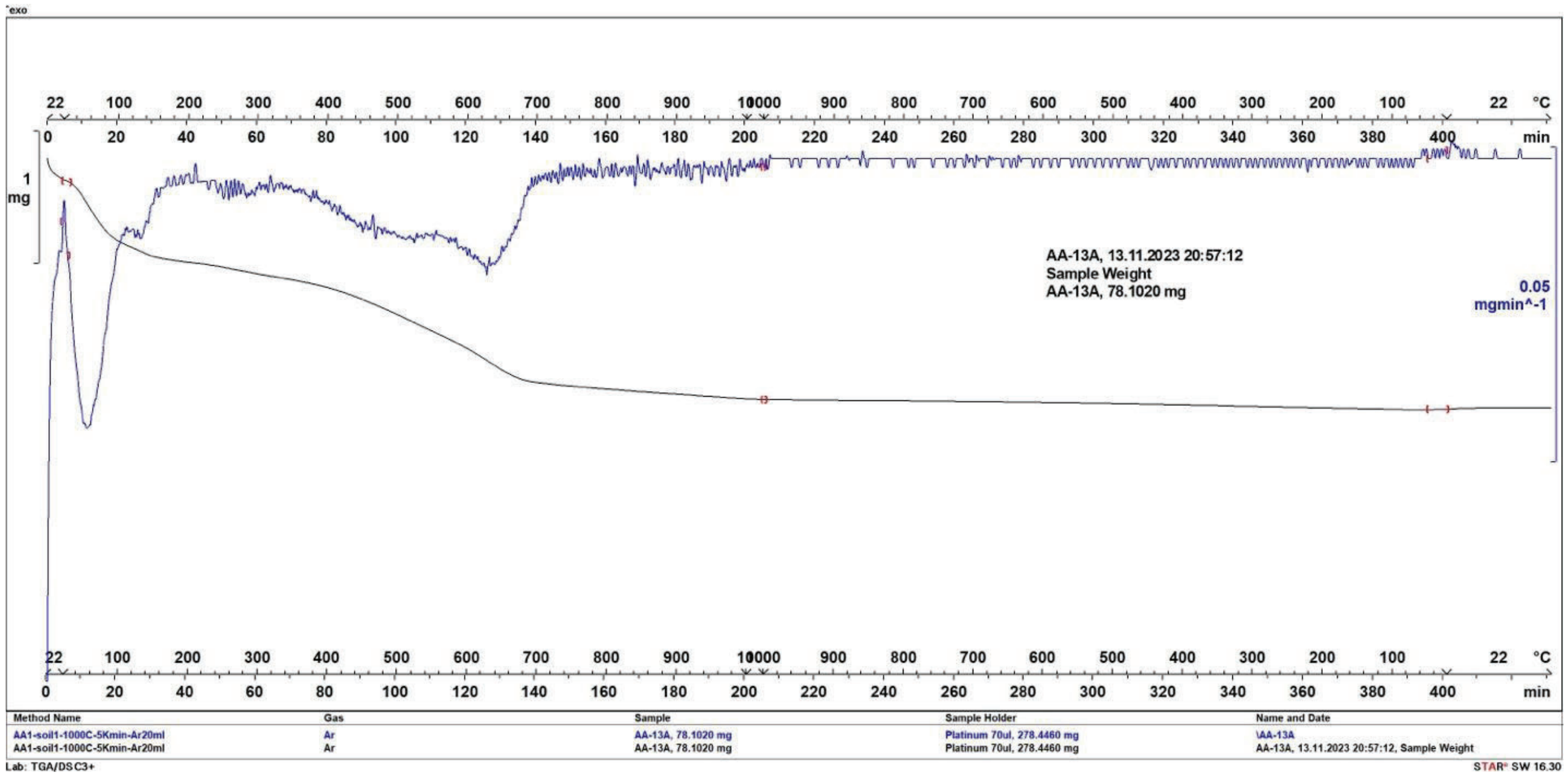

Fig. S18. Thermogravimetric analysis (TGA) measurements to detect thermal transformations in soil samples and to validate appropriate degassing temperatures. The results indicate minimal overall weight loss (~2mg) upon heating the samples up to 1000°C. The most substantial weight change occurs between 22°C and 100°C, corresponding to the evaporation of water molecules. A second, more gradual weight loss is observed between 400°C and 700°C, likely associated with the combustion of organic carbon. However, the total mass loss remains small, reflecting the low organic carbon content in the soil (0.17 C%).

A.

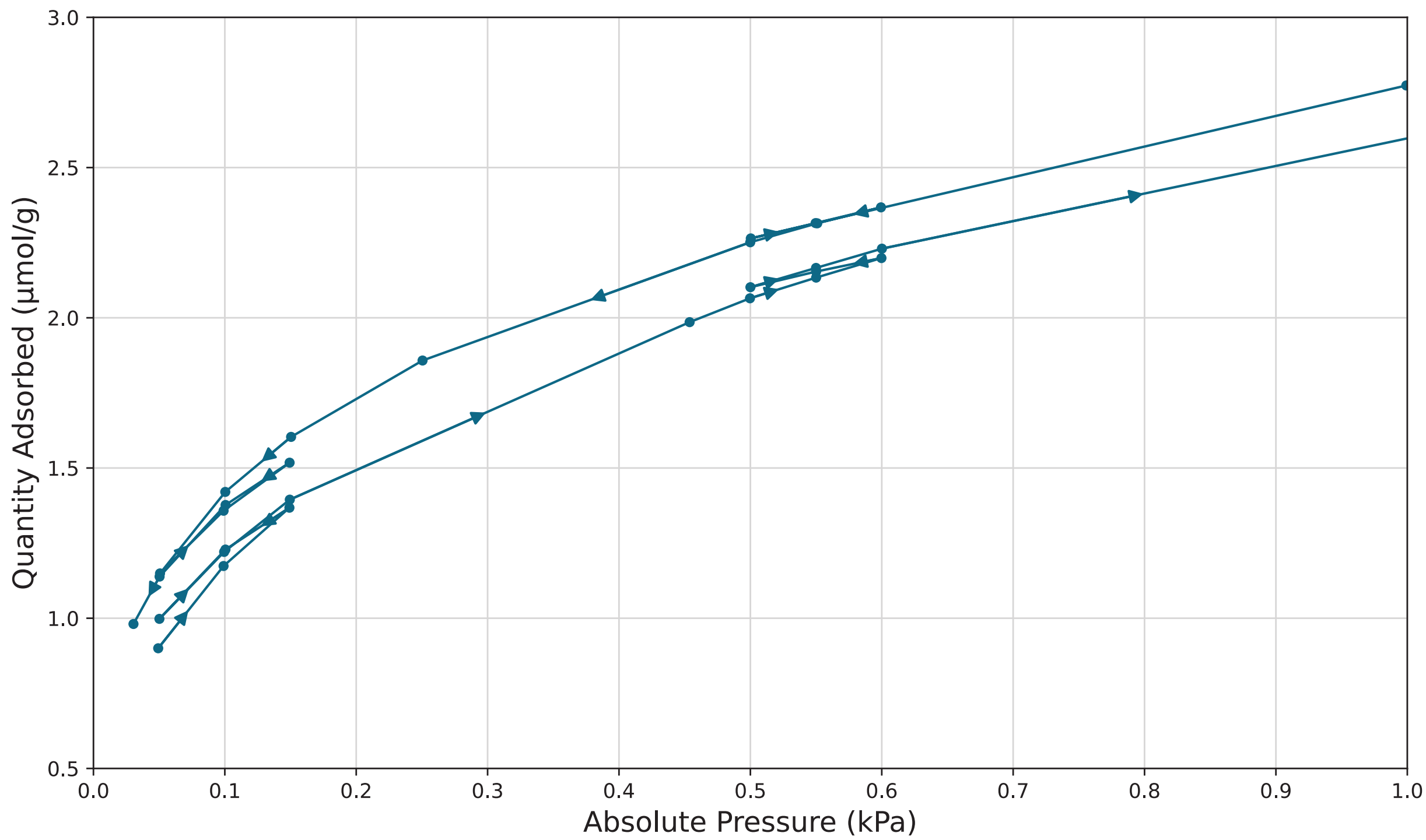

B.

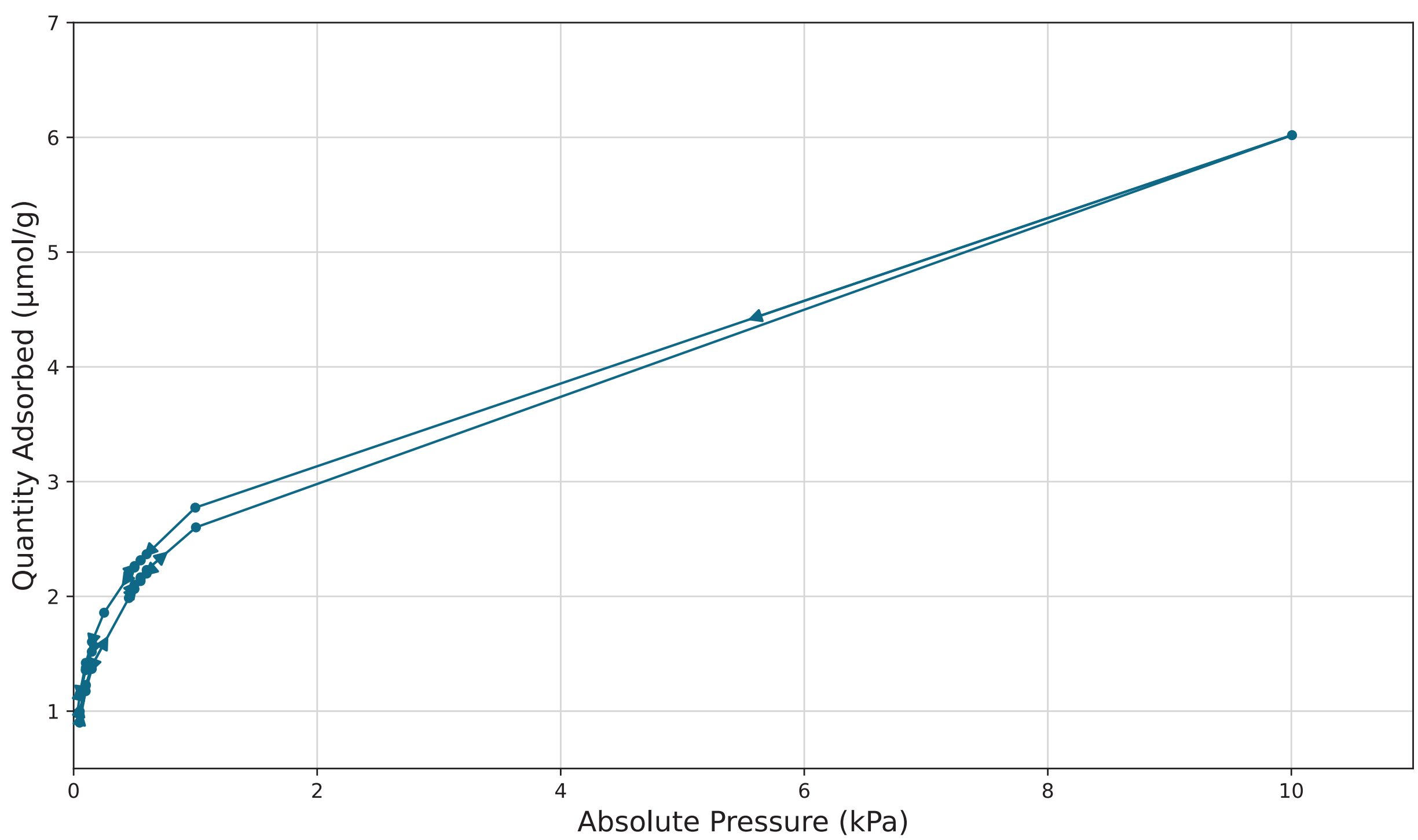

Figure S19. CO_2 adsorption-desorption isotherm that includes hysteresis loops: (A) for soil-relevant conditions of 500-1500 ppm (0.05-0.15 kPa) and 5000-6500 ppm (0.5-0.6 kPa); (B) the adsorption-desorption hysteresis loop that occurs after reaching 10 kPa CO_2 absolute pressure, a pressure far exceeding those observed in the studied soils.

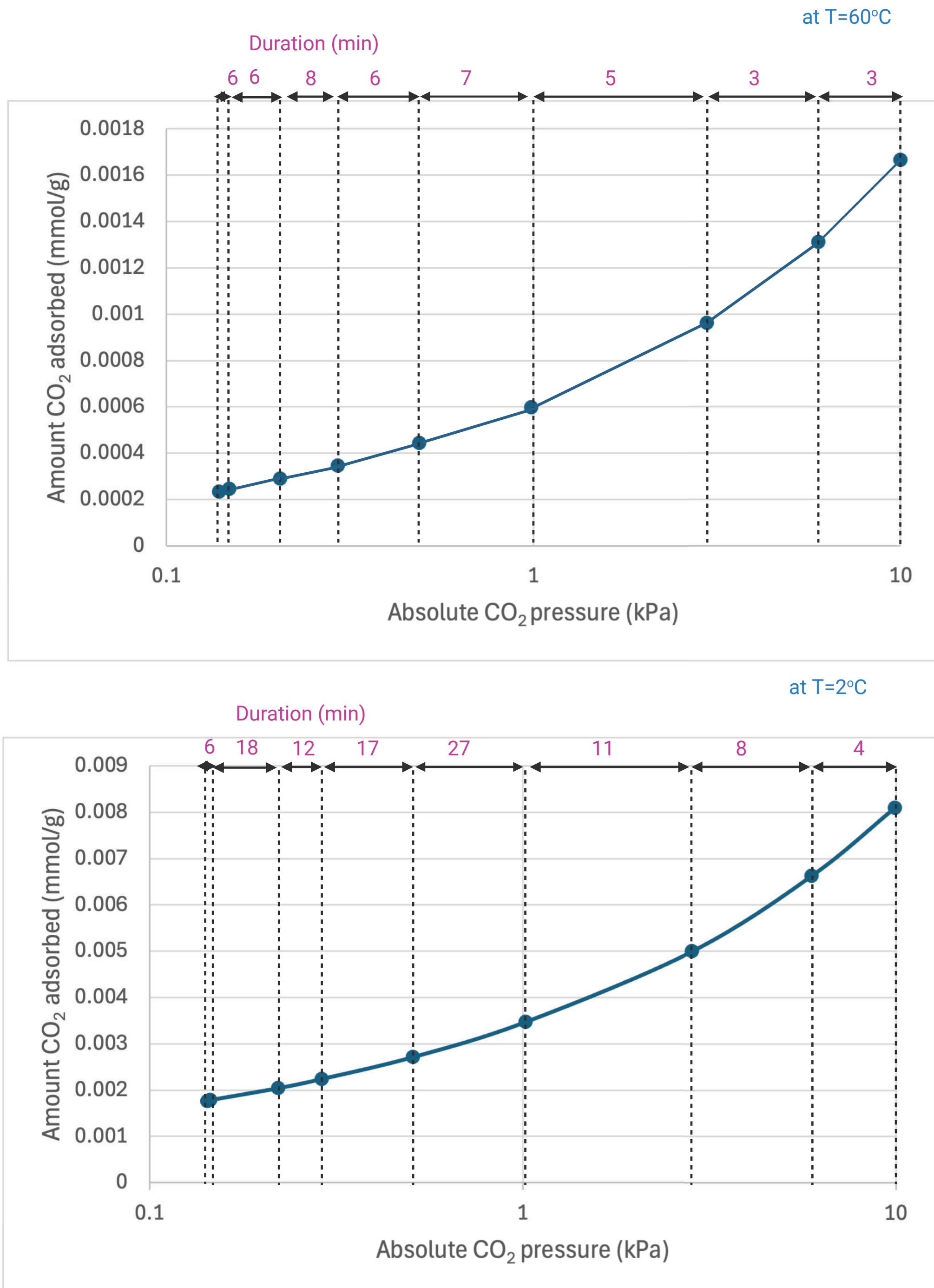

Fig. S20. An example showing the time taken to obtain each adsorption measurement with pressure increases at T=2 °C and 60 °C. The numbers at the top indicate the duration (in minutes) of each measurement step. Certain components were created in BioRender. A, A. (2026) https://BioRender.com/f0x7fu6

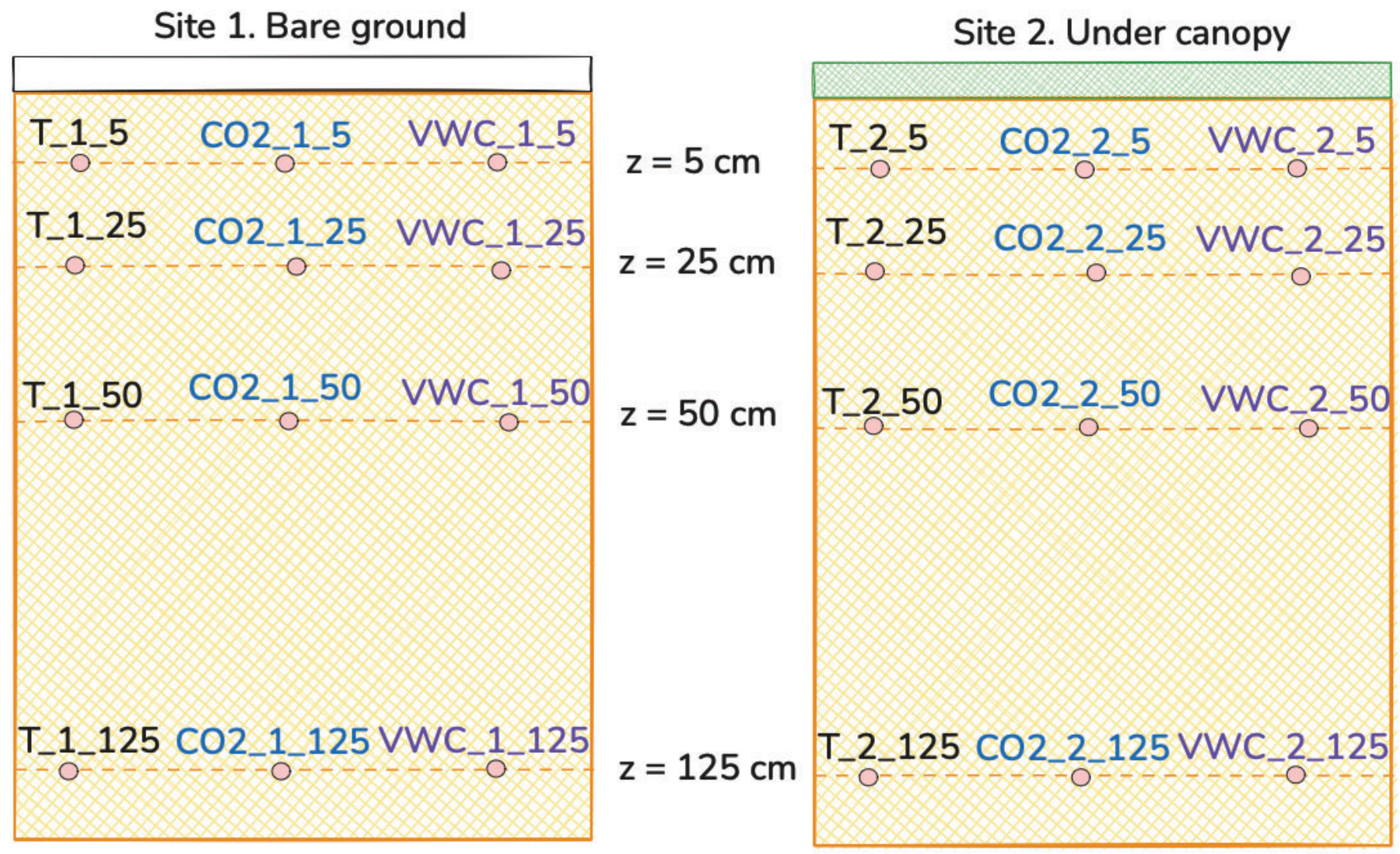

Fig. S21. Schematic representation of the two soil profiles: one is under bare ground (site 1), and the other is under the canopy of a Creosote bush (site 2). The schematic shows the soil depths (z) at which soil field measurements of temperature (T), carbon dioxide concentrations (CO_2), and volumetric water content (VWC) were recorded. On the figures, the dots and associated labels indicate the measured parameter (T, CO_2, or VWC), the study site (1 or 2), and the sampling depth (5, 25, 50, or 125 cm), all of which are separated by an underscore sign.

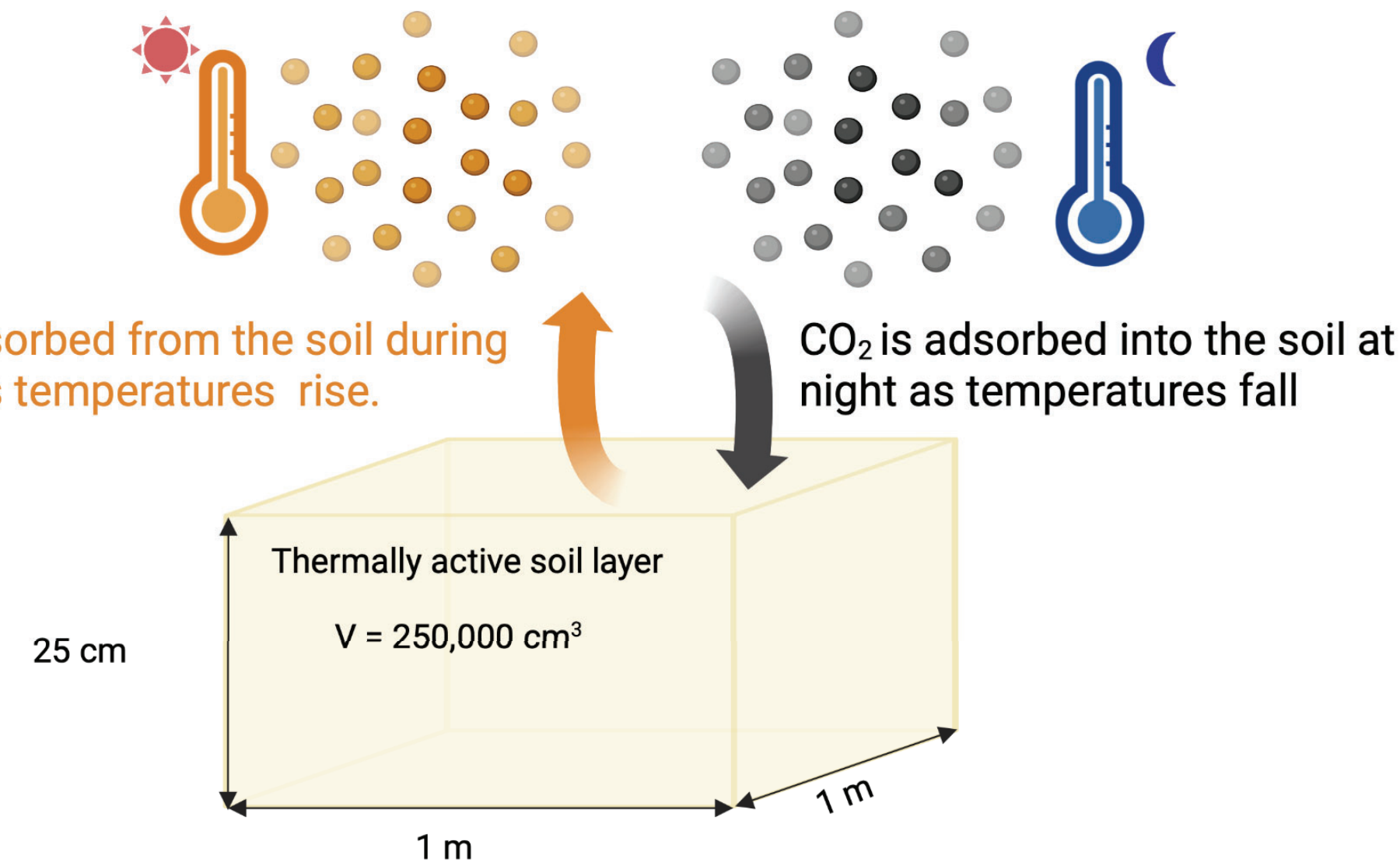

Fig. S22. Schematic representation of the diurnal thermally driven dynamics of CO_2 adsorption in the thermally active layer, upper 25 cm of the soil. During the daytime, as temperatures rise, CO_2 is desorbed from the soil into the atmosphere. In contrast, during the nighttime, when temperatures fall, CO_2 is adsorbed into the soil from the atmosphere. Created in BioRender. A, A. (2026) https://BioRender.com/nobxxg3

Table S1. Negative CO_2 Flux (μmol/m^2/ s) in the Mojave Desert Soils: Monthly Summary Statistics.

Month	Count of Days	Monthly Average	St. Dev.
2017-05	3	-0.052	0.052
2017-06	13	-0.017	0.015
2017-07	20	-0.027	0.022
2017-08	**31**	-0.044	0.028
2017-09	20	-0.038	0.024
2017-10	31	-0.056	0.034
2017-11	30	-0.056	0.032
2017-12	31	-0.065	0.034
2018-01	18	-0.045	0.029
2018-02	18	-0.048	0.032
2018-03	12	-0.030	0.023
2018-04	20	-0.030	0.020
2018-05	21	-0.031	0.024
2018-06	**30**	**-0.086**	0.039
2018-07	12	-0.097	0.055
2018-08	13	-0.045	0.026
2018-09	21	-0.040	0.026
2018-10	26	-0.046	0.030
2018-11	30	-0.060	0.030
2018-12	31	-0.049	0.027
2019-01	29	-0.050	0.030
2019-02	26	-0.081	0.055
2019-03	26	-0.034	0.027
2019-04	13	-0.012	0.009
2019-05	10	-0.049	0.047
2019-06	18	-0.060	0.038
2019-07	31	-0.074	0.035
2019-08	**31**	**-0.102**	0.041
2019-09	30	-0.102	0.045
2019-10	29	-0.122	0.049

Data S1. Reported negative nighttime soil surface CO_2 fluxes across global sites.

Data S2. Soil properties of the Mojave National Preserve, Southern California.

Data S3. Surface matrix of temperature, CO_2 partial pressure, and experimentally obtained amounts of CO_2 adsorbed.

Data S4. Calculation of the dissolution of CO_2 into soil water

Data S5. Summary statistics for CO_2 adsorption isotherms

Data S6. Calculation of CO_2 adsorption-desorption hysteresis loops

REFERENCES

1. B. Bond-Lamberty, A. Ballantyne, E. Berryman, E. Fluet-Chouinard, J. Jian, K. A. Morris, A. Rey, R. Vargas, Twenty years of progress, challenges, and opportunities in measuring and understanding soil respiration. *J.Geophys.Res. Biogeosci.* **129**, e2023JG007637 (2024).

2. S. Hashimoto, A. Ito, K. Nishina, Divergent data-driven estimates of global soil respiration. *Commun. Earth Environ.* **4**, 460 (2023).

3. W. Song, S. Chen, Y. Zhou, B. Wu, Y. Zhu, Q. Lu, G. Lin, Contrasting diel hysteresis between soil autotrophic and heterotrophic respiration in a desert ecosystem under different rainfall scenarios. *Sci. Rep.* **5**, 16779 (2015).

4. Y. Dusza, E. P. Sanchez-Cañete, J.-F. L. Galliard, R. Ferrière, S. Chollet, F. Massol, A. Hansart, S. Juarez, K. Dontsova, J. van Haren, Biotic soil-plant interaction processes explain most of hysteretic soil CO_2 efflux response to temperature in cross-factorial mesocosm experiment. *Sci. Rep.* **10**, 905 (2020).

5. Q. Zhang, G. G. Katul, R. Oren, E. Daly, S. Manzoni, D. Yang, The hysteresis response of soil CO_2 concentration and soil respiration to soil temperature. *J. Geophys. Res. Biogeo.* **120**, 1605–1618 (2015).

6. G. Wohlfahrt, L. F. Fenstermaker, J. A. Arnone Iii, Large annual net ecosystem CO_2 uptake of a Mojave Desert ecosystem. *Glob. Chang. Biol.* **14**, 1475–1487 (2008).

7. E. P. Hamerlynck, R. L. Scott, E. P. Sánchez-Cañete, G. A. Barron-Gafford, Nocturnal soil CO_2 uptake and its relationship to subsurface soil and ecosystem carbon fluxes in a Chihuahuan Desert shrubland. *J. Geophys. Res. Biogeo.* **118**, 1593–1603 (2013).

8. A. Cueva, T. H. M. Volkmann, J. van Haren, P. A. Troch, L. K. Meredith, Reconciling negative soil CO_2 fluxes: Insights from a large-scale experimental hillslope. *Soil Syst.* **3**, 10 (2019).

9. I. Langmuir, The adsorption of gases on plane surfaces of glass, mica and platinum. *J. Am. Chem. Soc.* **40**, 1361–1403 (1918).

10. K. Kaiser, G. Guggenberger, Mineral surfaces and soil organic matter. *Eur. J. Soil Sci.* **54**, 219–236 (2003).

11. I. C. Bourg, L. E. Beckingham, D. J. DePaolo, The nanoscale basis of CO_2 trapping for geologic storage. *Environ. Sci. Technol.* **49**, 10265–10284 (2015).

12. G. R. Davidson, A. Phillips-Housley, M. T. Stevens, Soil-zone adsorption of atmospheric CO_2 as a terrestrial carbon sink. *Geochim. Cosmochim. Acta* **106**, 44–50 (2013).

13. M. Kim, C. Lopez-Canfin, R. Lázaro, E. P. Sánchez-Cañete, B. Weber, Unravelling the main mechanism responsible for nocturnal CO_2 uptake by dryland soils. *Sci. Total Environ.* **926**, 171751 (2024).

14. C. Lopez-Canfin, R. Lázaro, E. P. Sánchez-Cañete, Disparate responses of soil-atmosphere CO_2 exchange to biophysical and geochemical factors over a biocrust ecological succession in the Tabernas Desert. *Geoderma* **425**, 116067 (2022).

15. C. Lopez-Canfin, R. Lázaro, E. P. Sánchez-Cañete, Water vapor adsorption by dry soils: A potential link between the water and carbon cycles. *Sci. Total Environ.* **824**, 153746 (2022).

16. N. Sagi, M. Zaguri, D. Hawlena, Soil CO_2 influx in drylands: A conceptual framework and empirical examination. *Soil Biol. Biochem.* **156**, 108209 (2021).

17. J. W. Raich, W. H. Schlesinger, The global carbon dioxide flux in soil respiration and its relationship to vegetation and climate. *Tellus B* **44**, 81–99 (1992).

18. C. León-Sobrino, J.-B. Ramond, G. Maggs-Kölling, D. A. Cowan, Nutrient acquisition, rather than stress response over diel cycles, drives microbial transcription in a hyper-arid Namib Desert soil. *Front. Microbiol.* **10**, 1054 (2019).

19. M. Spohn, K. Müller, C. Höschen, C. W. Mueller, S. Marhan, Dark microbial CO_2 fixation in temperate forest soils increases with CO_2 concentration. *Glob. Chang. Biol.* **26**, 1926–1935 (2020).

20. Z. Liu, Y. Sun, Y. Zhang, W. Feng, Z. Lai, S. Qin, Soil microbes transform inorganic carbon into organic carbon by dark fixation pathways in desert soil. *JGR Biogeosci.* **126**, e2020JG006047 (2021).

21. A. N. Parsons, J. E. Barrett, D. H. Wall, R. A. Virginia, Soil carbon dioxide flux in Antarctic dry valley ecosystems. *Ecosystems* **7**, 286–295 (2004).

22. J. Xie, Y. Li, C. Zhai, C. Li, Z. Lan, CO_2 absorption by alkaline soils and its implication to the global carbon cycle. *Environ. Geol.* **56**, 953–961 (2009).

23. E. L. Yates, A. M. Detweiler, L. T. Iraci, B. M. Bebout, C. P. McKay, K. Schiro, E. J. Sheffner, C. A. Kelley, J. M. Tadić, M. Loewenstein, Assessing the role of alkaline soils on the carbon cycle at a playa site. *Environ. Earth Sci.* **70**, 1047–1056 (2013).

24. J. Ma, Z.-Y. Wang, B. A. Stevenson, X.-J. Zheng, Y. Li, An inorganic CO_2 diffusion and dissolution process explains negative CO_2 fluxes in saline/alkaline soils. *Sci. Rep.* **3**, 2025 (2013).

25. K. Fa, Z. Liu, Y. Zhang, S. Qin, B. Wu, J. Liu, Abiotic carbonate dissolution traps carbon in a semiarid desert. *Sci. Rep.* **6**, 23570 (2016).

26. Z.-Y. Wang, J.-B. Xie, Y.-G. Wang, Y. Li, Biotic and abiotic contribution to diurnal soil CO_2 fluxes from saline/alkaline soils. *Sci. Rep.* **10**, 5396 (2020).

27. G. E. Maurer, L. N. Lammers, R. G. Amundson, MojaveCarbon Climosequence, HYDROSHARE (2019); www.hydroshare.org/resource/d01662d827f34170a5fd3589e468d06b.

28. A. Abramova, M. Getenet, G. Benjamin, R. Amundson, Gas Adsorption Isotherms and BET Surface Area Data for the Mojave Desert Soils and Clays Minerals, Zenodo (2026); https://doi.org/10.5281/zenodo.19226405.

29. F. E. Moyano, S. Manzoni, C. Chenu, Responses of soil heterotrophic respiration to moisture availability: An exploration of processes and models. *Soil Biol. Biochem.* **59**, 72–85 (2013).

30. C. T. Johnston, T. S. Zwier, T. A. Ho, G. Xu, Y. Wang, S. B. Rempe, N. Dasgupta, M. Mills, R. Welsh, D. L. Bish, Competitive sorption of H_2O and CO_2 in clay mineral interlayers. *J. Phys. Chem. C* **129**, 7832–7845 (2025).

31. A.-Y. Song, J. Young, J. Wang, S. N. Fricke, K. Piscina, R. Giovine, S. Garcia, M. van der Spek, J. A. Reimer, Discerning molecular-level CO_2 adsorption behavior in amine-modified sorbents within a controlled CO_2/H_2O environment towards direct air capture. *J. Mater. Chem. A* **12**, 25875–25886 (2024).

32. C. Guan, N. Chen, L. Qiao, C. Zhao, Photosynthesis regulates the diel hysteresis pattern between soil respiration and soil temperature in a steppe grassland. *Geoderma* **408**, 115561 (2022).

33. Y. Gao, Z. Zhao, Y. Zhang, J. Liu, Response of abiotic soil CO_2 flux to the difference in air-soil temperature in a desert. *Sci. Total Environ.* **785**, 147377 (2021).

34. H. Kim, S. R. Rao, E. A. Kapustin, L. Zhao, S. Yang, O. M. Yaghi, E. N. Wang, Adsorption-based atmospheric water harvesting device for arid climates. *Nat. Commun.* **9**, 1191 (2018).

35. W. H. Schlesinger, An evaluation of abiotic carbon sinks in deserts. *Glob. Chang. Biol.* **23**, 25–27 (2017).

36. Y. Chen, H. Lu, H. Wu, J. Wang, N. Lyu, Global desert variation under climatic impact during 1982–2020. *Sci. China Earth Sci.* **66**, 1062–1071 (2023).

37. P. R. Shukla, J. Skeg, E. C. Buendia, V. Masson-Delmotte, H.-O. Pörtner, D. C. Roberts, P. Zhai, R. Slade, S. Connors, S. Van Diemen, "Climate Change and Land: An IPCC special report on climate change, desertification, land degradation, sustainable land management, food security, and greenhouse gas fluxes in terrestrial ecosystems" (IPCC, 2019).

38. A. J. Ringsby, C. M. Ross, K. Maher, Sorption of soil carbon dioxide by biochar and engineered porous carbons. *Environ. Sci. Technol.* **58**, 8313–8325 (2024).

39. X. Gui, X. Xu, Z. Zhang, L. Hu, W. Huang, L. Zhao, X. Cao, Biochar-amended soil can further sorb atmospheric CO_2 for more carbon sequestration. *Commun. Earth Environ.* **6**, 5 (2025).

40. S. Helmrich, A. J. Ringsby, K. Maher, Reactive transport simulation of organic and inorganic carbon cycling following carbon dioxide sorption onto soil amendments in drylands. *Front. Clim.* **7**, 1505472 (2025).

41. R. Hesterberg, U. Siegenthaler, Production and stable isotopic composition of CO_2 in a soil near Bern, Switzerland. *Tellus B* **43**, 197–205 (1991).

42. G. D. Farquhar, J. Lloyd, J. A. Taylor, L. B. Flanagan, J. P. Syvertsen, K. T. Hubick, S. C. Wong, J. R. Ehleringer, Vegetation effects on the isotope composition of oxygen in atmospheric CO_2. *Nature* **363**, 439–443 (1993).

43. T. Rahn, J. M. Eiler, Experimental constraints on the fractionation of $^{13}C/^{12}C$ and $^{18}O/^{16}O$ ratios due to adsorption of CO_2 on mineral substrates at conditions relevant to the surface of Mars. *Geochim. Cosmochim. Acta* **65**, 839–846 (2001).

44. T. E. Larson, D. O. Breecker, Adsorption isotope effects for carbon dioxide from illite- and quartz-packed column experiments. *Chem. Geol.* **370**, 58–68 (2014).

45. F. P. Fanale, W. A. Cannon, Adsorption on the Martian regolith. *Nature* **230**, 502–504 (1971).

46. B. M. Jakosky, C. S. Edwards, Inventory of CO_2 available for terraforming Mars. *Nat Astron* **2**, 634–639 (2018).

47. P. B. Buhler, S. Piqueux, Obliquity-driven CO_2 exchange between Mars' atmosphere, regolith, and polar cap. *J. Geophys. Res. Planets* **126**, e2020JE006759 (2021).

48. L. Greenspan, Humidity fixed points of binary saturated aqueous solutions. *J. Res. Natl. Bur. Stand. A Phys. Chem.* **81A**, 89–96 (1977).

49. T. Wang, A. Hamann, D. Spittlehouse, C. Carroll, Locally downscaled and spatially customizable climate data for historical and future periods for North America. *PLOS ONE* **11**, e0156720 (2016).

50. J. V. Mills, Controls on Inorganic Calcite Growth From the Nano to Field Scale, Proquest (2021); www.proquest.com/openview/e1477467f0fa3a7ccf263e0fb5cc1c21/1?pq-origsite=gscholar&cbl=18750&diss=y.

51. B. F. Cox, J. W. Hillhouse, L. A. Owen, "Pliocene and Pleistocene Evolution of the Mojave River, and Associated Tectonic Development of the Transverse Ranges and Mojave Desert, Based on Borehole Stratigraphy Studies and Mapping of Landforms and Sediments near Victorville, California" in *Paleoenvironments and Paleohydrology of the Mojave and Southern Great Basin Deserts*, Y. Enzel, S. G. Wells, N. Lancaster, Eds. (Geological Society of America, 2003), pp. 1–42.

52. R. J. Pruett, H. L. Webb, Sampling and analysis of KGa-1B well-crystallized kaolin source clay. *Clays Clay Miner.* **41**, 514–519 (1993).

53. R. G. Amundson, O. A. Chadwick, J. M. Sowers, H. E. Doner, Soil evolution along an altitudinal transect in the eastern Mojave Desert of Nevada, USA. *Geoderma* **43**, 349–371 (1989).

54. J. W. Harden, *Soils Developed in Granitic Alluvium near Merced, California* (Government Printing Office, 1987).

55. T. Saito, H. Fujimaki, H. Yasuda, M. Inoue, Empirical temperature calibration of capacitance probes to measure soil water. *Soil Sci. Soc. Am. J.* **73**, 1931–1937 (2009).

56. G. R. Blake, K. H. Hartge, "Bulk density" in *Methods of Soil Analysis*, A. Klute, Ed. (John Wiley & Sons, Ltd, 1986), pp. 363–375.

57. B. H. Sheldrick, C. Wang, "Particle size distribution" in *Soil Sampling and Methods of Analysis*, M. R. Carter, Ed. (CRC Press, 1993), pp. 499–511.

58. J. Tang, L. Misson, A. Gershenson, W. Cheng, A. H. Goldstein, Continuous measurements of soil respiration with and without roots in a ponderosa pine plantation in the Sierra Nevada Mountains. *Agric. For. Meteorol.* **132**, 212–227 (2005).

59. R. Vargas, M. F. Allen, Dynamics of fine root, fungal rhizomorphs, and soil respiration in a mixed temperate forest: integrating sensors and observations. *Vadose Zone J.* **7**, 1055–1064 (2008).

60. P. Moldrup, T. Olesen, T. Yamaguchi, P. Schjønning, D. E. Rolston, Modeling diffusion and reaction in soils: IX. The Buckingham-Burdine-Campbell equation for gas diffusivity in undisturbed soil. *Soil Sci.* **164**, 542–551 (1999).

61. R. Vargas, M. Detto, D. D. Baldocchi, M. F. Allen, Multiscale analysis of temporal variability of soil CO_2 production as influenced by weather and vegetation. *Glob. Chang. Biol.* **16**, 1589–1605 (2010).

62. T. J. Kessler, C. F. Harvey, The global flux of carbon dioxide into groundwater. *Geophys. Res. Lett.* **28**, 279–282 (2001).

63. L. N. Plummer, E. Busenberg, The solubilities of calcite, aragonite and vaterite in CO_2-H_2O solutions between 0 and 90°C, and an evaluation of the aqueous model for the system $CaCO_3$-CO_2-H_2O. *Geochim. Cosmochim. Acta* **46**, 1011–1040 (1982).

64. B. A. Ball, R. A. Virginia, J. E. Barrett, A. N. Parsons, D. H. Wall, Interactions between physical and biotic factors influence CO_2 flux in Antarctic dry valley soils. *Soil Biol. Biochem.* **41**, 1510–1517 (2009).

65. B. A. Ball, R. A. Virginia, Controls on diel soil CO_2 flux across moisture gradients in a polar desert. *Antarct. Sci.* **27**, 527–534 (2015).

66. N. Bekin, N. Agam, Rethinking the deployment of static chambers for CO_2 flux measurement in dry desert soils. *Biogeosciences* **20**, 3791–3802 (2023).

67. A.-M. Doucet, F. Jones, K. E. Raymond, G. Dipple, T. A. Black, B. Ladd, K. U. Mayer, Quantitative analysis of diurnal CO_2 flux variations above an alkaline playa. *Vadose Zone J.* **22**, e20292 (2023).

68. K. Fa, Y. Zhang, G. Lei, B. Wu, S. Qin, J. Liu, W. Feng, Z. Lai, Underestimation of soil respiration in a desert ecosystem. *Catena* **162**, 23–28 (2018).

69. X. Li, D. Guo, C. Zhang, D. Niu, H. Fu, C. Wan, Contribution of root respiration to total soil respiration in a semi-arid grassland on the Loess Plateau, China. *Sci. Total Environ.* **627**, 1209–1217 (2018).

70. J. Liu, W. Feng, Y. Zhang, X. Jia, B. Wu, S. Qin, K. Fa, Z. Lai, Abiotic CO_2 exchange between soil and atmosphere and its response to temperature. *Environ. Earth Sci.* **73**, 2463–2471 (2015).

71. J. Ma, R. Liu, Y. Li, Abiotic contribution to total soil CO_2 flux across a broad range of land-cover types in a desert region. *J. Arid. Land* **9**, 13–26 (2017).

72. F. L. Shanhun, P. C. Almond, T. J. Clough, C. M. S. Smith, Abiotic processes dominate CO_2 fluxes in Antarctic soils. *Soil Biol. Biochem.* **53**, 99–111 (2012).

73. A. Thomazini, M. R. Francelino, A. B. Pereira, A. L. Schünemann, E. S. Mendonça, P. H. A. Almeida, C. E. G. R. Schaefer, Geospatial variability of soil CO_2–C exchange in the main terrestrial ecosystems of Keller Peninsula, Maritime Antarctica. *Sci. Total Environ.* **562**, 802–811 (2016).

74. F. Yang, M. Ali, X. Zheng, Q. He, X. Yang, W. Huo, F. Liang, S. Wang, Diurnal dynamics of soil respiration and the influencing factors for three land-cover types in the hinterland of the Taklimakan Desert, China. *J. Arid Land* **9**, 568–579 (2017).

75. F. Yang, J. Huang, Q. He, X. Zheng, C. Zhou, H. Pan, W. Huo, H. Yu, X. Liu, L. Meng, Impact of differences in soil temperature on the desert carbon sink. *Geoderma* **379**, 114636 (2020).

76. F. Yang, J. Huang, C. Zhou, X. Yang, A. Mamtimin, X. Zheng, W. Huo, F. Ji, D. Han, L. Meng, J. Gao, M. Song, Y. Wang, C. Zhu, Desert abiotic carbon sequestration weakening by precipitation. *Environ. Sci. Technol.* **57**, 7174–7184 (2023).

77. X. Yu, X. Li, L. Xu, M. Wang, J. Zhang, M. Jiang, G. Wang, Diurnal variation of soil CO_2 efflux and its optimal measuring time-window of temperate meadow steppes in western Songnen Plain, China. *Chin. Geogr. Sci.* **26**, 518–526 (2016).

78. T. Zhang, G. Wang, Y. Yang, T. Mao, X. Chen, Non-growing season soil CO_2 flux and its contribution to annual soil CO_2 emissions in two typical grasslands in the permafrost region of the Qinghai-Tibet Plateau. *Eur. J. Soil Biol.* **71**, 45–52 (2015).

79. X. Zhou, Y. Zhang, Seasonal pattern of soil respiration and gradual changing effects of nitrogen addition in a soil of the Gurbantunggut Desert, northwestern China. *Atmos. Environ.* **85**, 187–194 (2014).

80. N. Bekin, D. S. Ashilenje, A. Chehbouni, L. Bouchaou, D. Kool, N. Agam, Pioneering evidence of the dynamics of water vapor and CO_2 fluxes in Sahara Desert soils. *J. Geophys. Res. Biogeosci.* **130**, e2025JG009193 (2025).
